# Prediction models for diagnosis and prognosis of covid-19: systematic review and critical appraisal 

**DOI:** 10.1136/bmj.m1328

**Published:** 2020-04-07

**Authors:** Laure Wynants, Ben Van Calster, Gary S Collins, Richard D Riley, Georg Heinze, Ewoud Schuit, Elena Albu, Banafsheh Arshi, Vanesa Bellou, Marc M J Bonten, Darren L Dahly, Johanna A Damen, Thomas P A Debray, Valentijn M T de Jong, Maarten De Vos, Paula Dhiman, Joie Ensor, Shan Gao, Maria C Haller, Michael O Harhay, Liesbet Henckaerts, Pauline Heus, Jeroen Hoogland, Mohammed Hudda, Kevin Jenniskens, Michael Kammer, Nina Kreuzberger, Anna Lohmann, Brooke Levis, Kim Luijken, Jie Ma, Glen P Martin, David J McLernon, Constanza L Andaur Navarro, Johannes B Reitsma, Jamie C Sergeant, Chunhu Shi, Nicole Skoetz, Luc J M Smits, Kym I E Snell, Matthew Sperrin, René Spijker, Ewout W Steyerberg, Toshihiko Takada, Ioanna Tzoulaki, Sander M J van Kuijk, Bas C T van Bussel, Iwan C C van der Horst, Kelly Reeve, Florien S van Royen, Jan Y Verbakel, Christine Wallisch, Jack Wilkinson, Robert Wolff, Lotty Hooft, Karel G M Moons, Maarten van Smeden

**Affiliations:** 1 Department of Epidemiology, CAPHRI Care and Public Health Research Institute, Maastricht University, Maastricht, Netherlands; 2 Department of Development and Regeneration, KU Leuven, Leuven, Belgium; 3 Department of Biomedical Data Sciences, Leiden University Medical Centre, Leiden, Netherlands; 4 Centre for Statistics in Medicine, Nuffield Department of Orthopaedics, Musculoskeletal Sciences, University of Oxford, Oxford, UK; 5 NIHR Oxford Biomedical Research Centre, John Radcliffe Hospital, Oxford, UK; 6 Centre for Prognosis Research, School of Medicine, Keele University, Keele, UK; 7 Section for Clinical Biometrics, Centre for Medical Statistics, Informatics and Intelligent Systems, Medical University of Vienna, Vienna, Austria; 8 Julius Center for Health Sciences and Primary Care, University Medical Center Utrecht, Utrecht University, Utrecht, Netherlands; 9 Cochrane Netherlands, University Medical Center Utrecht, Utrecht University, Utrecht, Netherlands; 10 Department of Hygiene and Epidemiology, University of Ioannina Medical School, Ioannina, Greece; 11 Department of Medical Microbiology, University Medical Centre Utrecht, Utrecht, Netherlands; 12 HRB Clinical Research Facility, Cork, Ireland; 13 School of Public Health, University College Cork, Cork, Ireland; 14 Smart Data Analysis and Statistics BV, Utrecht, Netherlands; 15 Department of Electrical Engineering, ESAT Stadius, KU Leuven, Leuven, Belgium; 16 Ordensklinikum Linz, Hospital Elisabethinen, Department of Nephrology, Linz, Austria; 17 Department of Biostatistics, Epidemiology and Informatics, Perelman School of Medicine, University of Pennsylvania, Philadelphia, PA, USA; 18 Palliative and Advanced Illness Research Center and Division of Pulmonary and Critical Care Medicine, Department of Medicine, Perelman School of Medicine, University of Pennsylvania, Philadelphia, PA, USA; 19 Department of Microbiology, Immunology and Transplantation, KU Leuven-University of Leuven, Leuven, Belgium; 20 Department of General Internal Medicine, KU Leuven-University Hospitals Leuven, Leuven, Belgium; 21 Population Health Research Institute, St. George's University of London, Cranmer Terrace, London, UK; 22 Department of Nephrology, Medical University of Vienna, Vienna, Austria; 23 Evidence-Based Oncology, Department I of Internal Medicine and Centre for Integrated Oncology Aachen Bonn Cologne Dusseldorf, Faculty of Medicine and University Hospital Cologne, University of Cologne, Cologne, Germany; 24 Department of Clinical Epidemiology, Leiden University Medical Centre, Leiden, Netherlands; 25 Division of Informatics, Imaging and Data Science, Faculty of Biology, Medicine and Health, Manchester Academic Health Science Centre, University of Manchester, Manchester, UK; 26 Institute of Applied Health Sciences, University of Aberdeen, Aberdeen, UK; 27 Centre for Biostatistics, University of Manchester, Manchester Academic Health Science Centre, Manchester, UK; 28 Centre for Epidemiology Versus Arthritis, Centre for Musculoskeletal Research, University of Manchester, Manchester Academic Health Science Centre, Manchester, UK; 29 Division of Nursing, Midwifery and Social Work, School of Health Sciences, University of Manchester, Manchester, UK; 30 Faculty of Biology, Medicine and Health, University of Manchester, Manchester, UK; 31 Amsterdam UMC, University of Amsterdam, Amsterdam Public Health, Medical Library, Netherlands; 32 Department of General Medicine, Shirakawa Satellite for Teaching And Research, Fukushima Medical University, Fukushima, Japan; 33 Department of Epidemiology and Biostatistics, Imperial College London School of Public Health, London, UK; 34 Department of Clinical Epidemiology and Medical Technology Assessment, Maastricht University Medical Centre+, Maastricht, Netherlands; 35 Department of Intensive Care Medicine, Maastricht University Medical Centre+, Maastricht University, Maastricht, Netherlands; 36 Epidemiology, Biostatistics and Prevention Institute, University of Zurich, Zurich, CH; 37 EPI-Centre, Department of Public Health and Primary Care, KU Leuven, Leuven, Belgium; 38 NIHR Community Healthcare Medtech and IVD cooperative, Nuffield Department of Primary Care Health Sciences, University of Oxford, Oxford, UK; 39 Charité Universitätsmedizin Berlin, corporate member of Freie Universität Berlin, Humboldt-Universität zu Berlin, Berlin, Germany; 40 Berlin Institute of Health, Berlin, Germany; 41 Kleijnen Systematic Reviews, York, UK

## Abstract

**Objective:**

To review and appraise the validity and usefulness of published and preprint reports of prediction models for prognosis of patients with covid-19, and for detecting people in the general population at increased risk of covid-19 infection or being admitted to hospital or dying with the disease.

**Design:**

Living systematic review and critical appraisal by the covid-PRECISE (Precise Risk Estimation to optimise covid-19 Care for Infected or Suspected patients in diverse sEttings) group.

**Data sources:**

PubMed and Embase through Ovid, up to 17 February 2021, supplemented with arXiv, medRxiv, and bioRxiv up to 5 May 2020.

**Study selection:**

Studies that developed or validated a multivariable covid-19 related prediction model.

**Data extraction:**

At least two authors independently extracted data using the CHARMS (critical appraisal and data extraction for systematic reviews of prediction modelling studies) checklist; risk of bias was assessed using PROBAST (prediction model risk of bias assessment tool).

**Results:**

126 978 titles were screened, and 412 studies describing 731 new prediction models or validations were included. Of these 731, 125 were diagnostic models (including 75 based on medical imaging) and the remaining 606 were prognostic models for either identifying those at risk of covid-19 in the general population (13 models) or predicting diverse outcomes in those individuals with confirmed covid-19 (593 models). Owing to the widespread availability of diagnostic testing capacity after the summer of 2020, this living review has now focused on the prognostic models. Of these, 29 had low risk of bias, 32 had unclear risk of bias, and 545 had high risk of bias. The most common causes for high risk of bias were inadequate sample sizes (n=408, 67%) and inappropriate or incomplete evaluation of model performance (n=338, 56%). 381 models were newly developed, and 225 were external validations of existing models. The reported C indexes varied between 0.77 and 0.93 in development studies with low risk of bias, and between 0.56 and 0.78 in external validations with low risk of bias. The Qcovid models, the PRIEST score, Carr’s model, the ISARIC4C Deterioration model, and the Xie model showed adequate predictive performance in studies at low risk of bias. Details on all reviewed models are publicly available at https://www.covprecise.org/.

**Conclusion:**

Prediction models for covid-19 entered the academic literature to support medical decision making at unprecedented speed and in large numbers. Most published prediction model studies were poorly reported and at high risk of bias such that their reported predictive performances are probably optimistic. Models with low risk of bias should be validated before clinical implementation, preferably through collaborative efforts to also allow an investigation of the heterogeneity in their performance across various populations and settings. Methodological guidance, as provided in this paper, should be followed because unreliable predictions could cause more harm than benefit in guiding clinical decisions. Finally, prediction modellers should adhere to the TRIPOD (transparent reporting of a multivariable prediction model for individual prognosis or diagnosis) reporting guideline.

**Systematic review registration:**

Protocol https://osf.io/ehc47/, registration https://osf.io/wy245.

**Readers’ note:**

This article is the final version of a living systematic review that has been updated over the past two years to reflect emerging evidence. This version is update 4 of the original article published on 7 April 2020 (*BMJ* 2020;369:m1328). Previous updates can be found as data supplements (https://www.bmj.com/content/369/bmj.m1328/related#datasupp). When citing this paper please consider adding the update number and date of access for clarity.

## Introduction

The novel coronavirus disease 2019 (covid-19) presents an important threat to global health. Since the outbreak in early December 2019 in the Hubei province of the People’s Republic of China, the number of patients confirmed to have the disease has exceeded 500 million as the disease spread globally, and the number of people infected is probably much higher. More than 6 million people have died from covid-19 (up to 24 May 2022).^1^ Despite public health responses aimed at containing the disease and delaying the spread, countries have been confronted with repeated surges disrupting health services^2^ ^3^ ^4^ ^5^ ^6^ and society at large. More recent outbreaks of the omicron variant led to important increases in the demand for test capacity, hospital beds, and medical equipment, while medical staff members also increasingly became infected themselves.^6^ While many national governments have now put an end to covid-19 restrictions, scientists warn that endemic circulation of SARS-CoV-2, perhaps with seasonal epidemic peaks, is likely to have a continued important disease burden.^7^ In addition, virus mutations can be unpredictable, and lack of effective surveillance or adequate response could enable the emergence of new epidemic or pandemic covid-19 patterns.^7^ ^8^ To mitigate the burden on the healthcare system, while also providing the best possible care for patients, reliable prognosis of covid-19 remains important to inform decisions regarding shielding, vaccination, treatment, and hospital or intensive care unit (ICU) admission. Prediction models that combine several variables or features to estimate the risk of people being infected or experiencing a poor outcome from the infection could assist medical staff in triaging patients when allocating limited healthcare resources.

The outbreak of covid-19 was accompanied by a surge of scientific evidence.^9^ The speed with which evidence about covid-19 has accumulated is unprecedented. To provide an overview of available prediction models, a living systematic review, with periodic updates, was conducted by the international covid-PRECISE (Precise Risk Estimation to optimise covid-19 Care for Infected or Suspected patients in diverse sEttings; https://www.covprecise.org/) group in collaboration with the Cochrane Prognosis Methods Group. Initially, the review included diagnostic and prognostic models. Owing to the current availability of testing for covid-19 infections, we restricted the focus to prognostic models in this new update. Hence our aim was to systematically review and critically appraise available prognostic models for detecting people in the general population at increased risk of covid-19 infection or being admitted to hospital or dying with the disease, and models to predict the prognosis or course of infection in patients with a covid-19 diagnosis. We included all prognostic model development and external validation studies. 

## Methods

We searched the publicly available, continuously updated publication list of the covid-19 living systematic review.^10^ We validated whether the list is fit for purpose (online supplementary material) and further supplemented it with studies on covid-19 retrieved from arXiv. The online supplementary material presents the search strings. We included studies if they developed or validated a multivariable model or scoring system, based on individual participant level data, to predict any covid-19 related outcome. These models included prognostic models to predict the course of infection in patients with covid-19; and prediction models to identify people in the general population at risk of covid-19 infection or at risk of being admitted to hospital or dying with the disease. Diagnostic models to predict the presence or severity of covid-19 in patients with suspected infection were included up to update 3 only, which can be found in the data supplements.

We searched the database repeatedly up to 17 February 2021 (supplementary table 1). As of the third update (search date 1 July 2020), we only include peer reviewed articles (indexed in PubMed and Embase through Ovid). Preprints (from bioRxiv, medRxiv, and arXiv) that were already included in previous updates of the systematic review remained included in the analysis. Reassessment took place after publication of a preprint in a peer reviewed journal and replaced the original assessment. No restrictions were made on the setting (eg, inpatients, outpatients, or general population), prediction horizon (how far ahead the model predicts), included predictors, or outcomes. Epidemiological studies that aimed to model disease transmission or fatality rates, and predictor finding studies, were excluded. We only included studies published in English. Starting with the second update, retrieved records were initially screened by a text analysis tool developed using artificial intelligence to prioritise sensitivity (supplementary material). Titles, abstracts, and full texts were screened for eligibility in duplicate by independent reviewers (pairs from LW, BVC, MvS, and KGMM) using EPPI-Reviewer,^11^ and discrepancies were resolved through discussion. 

Data extraction of included articles was done by two independent reviewers (from LW, BVC, GSC, TPAD, MCH, GH, KGMM, RDR, ES, LJMS, EWS, KIES, CW, AL, JM, TT, JAD, KL, JBR, LH, CS, MS, MCH, NS, NK, SMJvK, JCS, PD, CLAN, RW, GPM, IT, JYV, DLD, JW, FSvR, PH, VMTdJ, BCTvB, ICCvdH, DJM, MK, BL, EA, SG, BA, JH, KJ, SG, KR, JE, MH, VB, and MvS). Reviewers used a standardised data extraction form based on the CHARMS (critical appraisal and data extraction for systematic reviews of prediction modelling studies) checklist^12^ and PROBAST (prediction model risk of bias assessment tool; https://www.probast.org/) for assessing the reported prediction models.^13^ ^14^ We sought to extract each model’s predictive performance by using whatever measures were presented. These measures included any summaries of discrimination (the extent to which predicted risks discriminate between participants with and without the outcome), and calibration (the extent to which predicted risks correspond to observed risks) as recommended in the TRIPOD (transparent reporting of a multivariable prediction model for individual prognosis or diagnosis; https://www.tripod-statement.org/) statement.^15^ ^16^ Discrimination is often quantified by the C index (C index=1 if the model discriminates perfectly; C index=0.5 if discrimination is no better than chance). Calibration is often assessed graphically using calibration plots or quantified by the calibration intercept (which is zero when the risks are not systematically overestimated or underestimated) and calibration slope (which is one if the predicted risks are not too extreme or too moderate).^17^ We focused on performance statistics as estimated from the strongest available form of validation (in order of strength: external (evaluation in an independent database), internal (bootstrap validation, cross validation, random training test splits, temporal splits), apparent (evaluation by using exactly the same data used for development)). Any discrepancies in data extraction were discussed between reviewers, and remaining conflicts were resolved by LW or MvS. The online supplementary material provides details on data extraction. Some studies investigated multiple models and some models were investigated in multiple studies (that is, in external validation studies). The unit of analysis was a model within a study, unless stated otherwise. We considered aspects of PRISMA (preferred reporting items for systematic reviews and meta-analyses)^18^ and TRIPOD^15^ ^16^ in reporting our study. Details on all reviewed studies and prediction models are publicly available at https://www.covprecise.org/. 

### Patient and public involvement

Severe covid-19 survivors and lay people participated by discussing their perspectives, providing advice, and acting as partners in writing a lay summary of the project’s aims and results (available at https://www.covprecise.org/project/), thereby taking part in the implementation of knowledge distribution. Owing to the initial emergency situation of the covid-19 pandemic, we did not involve patients or the public in the design and conduct of this living review in March 2020, but the study protocol and preliminary results were immediately publicly available on https://osf.io/ehc47/, medRxiv, and https://www.covprecise.org/living-review/.

## Results

**Fig 1:**
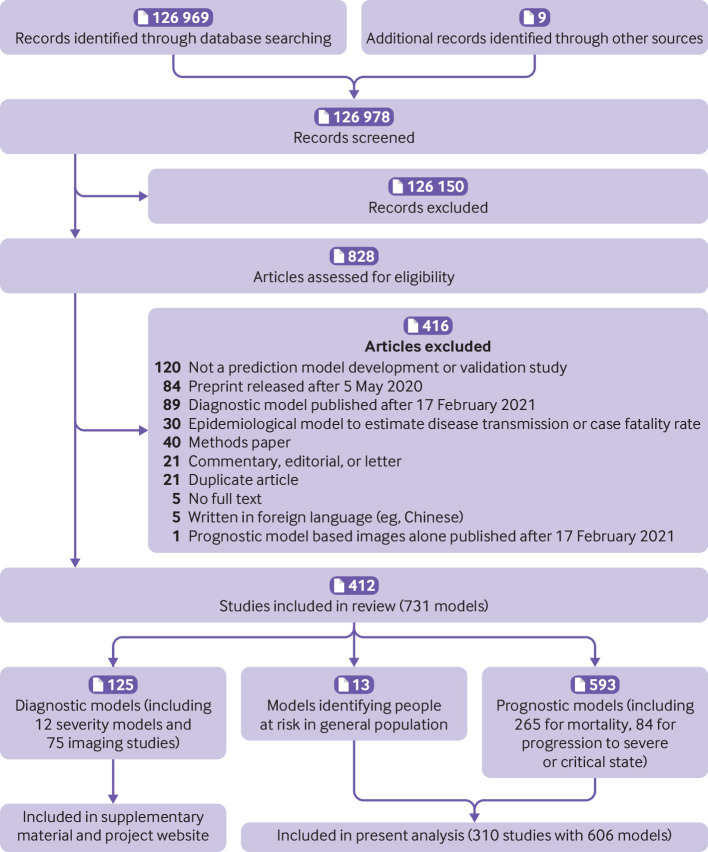
PRISMA (preferred reporting items for systematic reviews and meta-analyses) flowchart of study inclusions and exclusions

We identified 126 969 records through our systematic search, of which 89 566 were identified in the present search update (supplementary table 1, fig 1). We included a further nine studies that were publicly available but were not detected by our search. Of 126 978 titles, 828 studies were retained for abstract and full text screening. We included 412 studies describing 731 prediction models or validations, of which 243 studies with 499 models or validations were newly included in the present update (supplementary table 1).^19^ ^20^ ^21^ ^22^ ^23^ ^24^ ^25^ ^26^ ^27^ ^28^ ^29^ ^30^ ^31^ ^32^ ^33^ ^34^ ^35^ ^36^ ^37^ ^38^ ^39^ ^40^ ^41^ ^42^ ^43^ ^44^ ^45^ ^46^ ^47^ ^48^ ^49^ ^50^ ^51^ ^52^ ^53^ ^54^ ^55^ ^56^ ^57^ ^58^ ^59^ ^60^ ^61^ ^62^ ^63^ ^64^ ^65^ ^66^ ^67^ ^68^ ^69^ ^70^ ^71^ ^72^ ^73^ ^74^ ^75^ ^76^ ^77^ ^78^ ^79^ ^80^ ^81^ ^82^ ^83^ ^84^ ^85^ ^86^ ^87^ ^88^ ^89^ ^90^ ^91^ ^92^ ^93^ ^94^ ^95^ ^96^ ^97^ ^98^ ^99^ ^100^ ^101^ ^102^ ^103^ ^104^ ^105^ ^106^ ^107^ ^108^ ^109^ ^110^ ^111^ ^112^ ^113^ ^114^ ^115^ ^116^ ^117^ ^118^ ^119^ ^120^ ^121^ ^122^ ^123^ ^124^ ^125^ ^126^ ^127^ ^128^ ^129^ ^130^ ^131^ ^132^ ^133^ ^134^ ^135^ ^136^ ^137^ ^138^ ^139^ ^140^ ^141^ ^142^ ^143^ ^144^ ^145^ ^146^ ^147^ ^148^ ^149^ ^150^ ^151^ ^152^ ^153^ ^154^ ^155^ ^156^ ^157^ ^158^ ^159^ ^160^ ^161^ ^162^ ^163^ ^164^ ^165^ ^166^ ^167^ ^168^ ^169^ ^170^ ^171^ ^172^ ^173^ ^174^ ^175^ ^176^ ^177^ ^178^ ^179^ ^180^ ^181^ ^182^ ^183^ ^184^ ^185^ ^186^ ^187^ ^188^ ^189^ ^190^ ^191^ ^192^ ^193^ ^194^ ^195^ ^196^ ^197^ ^198^ ^199^ ^200^ ^201^ ^202^ ^203^ ^204^ ^205^ ^206^ ^207^ ^208^ ^209^ ^210^ ^211^ ^212^ ^213^ ^214^ ^215^ ^216^ ^217^ ^218^ ^219^ ^220^ ^221^ ^222^ ^223^ ^224^ ^225^ ^226^ ^227^ ^228^ ^229^ ^230^ ^231^ ^232^ ^233^ ^234^ ^235^ ^236^ ^237^ ^238^ ^239^ ^240^ ^241^ ^242^ ^243^ ^244^ ^245^ ^246^ ^247^ ^248^ ^249^ ^250^ ^251^ ^252^ ^253^ ^254^ ^255^ ^256^ ^257^ ^258^ ^259^ ^260^ ^261^ ^262^ ^263^ ^264^ ^265^ ^266^ ^267^ ^268^ ^269^ ^270^ ^271^ ^272^ ^273^ ^274^ ^275^ ^276^ ^277^ ^278^ ^279^ ^280^ ^281^ ^282^ ^283^ ^284^ ^285^ ^286^ ^287^ ^288^ ^289^ ^290^ ^291^ ^292^ ^293^ ^294^ ^295^ ^296^ ^297^ ^298^ ^299^ ^300^ ^301^ ^302^ ^303^ ^304^ ^305^ ^306^ ^307^ ^308^ ^309^ ^310^ ^311^ ^312^ ^313^ ^314^ ^315^ ^316^ ^317^ ^318^ ^319^ ^320^ ^321^ ^322^ ^323^ ^324^ ^325^ ^326^ ^327^ ^328^ ^329^ ^330^ ^331^ ^332^ ^333^ ^334^ ^335^ ^336^ ^337^ ^338^ ^339^ ^340^ ^341^ ^342^ ^343^ ^344^ ^345^ ^346^ ^347^ ^348^ ^349^ ^350^ ^351^ ^352^ ^353^ ^354^ ^355^ ^356^ ^357^ ^358^ ^359^ ^360^ ^361^ ^362^ ^363^ ^364^ ^365^ ^366^ ^367^ ^368^ ^369^ ^370^ ^371^ ^372^ ^373^ ^374^ ^375^ ^376^ ^377^ ^378^ ^379^ ^380^ ^381^ ^382^ ^383^ ^384^ ^385^ ^386^ ^387^ ^388^ ^389^ ^390^ ^391^ ^392^ ^393^ ^394^ ^395^ ^396^ ^397^ ^398^ ^399^ ^400^ ^401^ ^402^ ^403^ ^404^ ^405^ ^406^ ^407^ ^408^ ^409^ ^410^ ^411^ ^412^ ^413^ ^414^ ^415^ ^416^ ^417^ ^418^ ^419^ ^420^ ^421^ ^422^ ^423^ ^424^ ^425^ ^426^ ^427^ ^428^ ^429^ ^430^ Of these, 310 studies describing 606 prognostic models or validations of prognostic models are included in the current analysis: 13 prognostic models for developing covid-19 in the general population and 593 prognostic models for predicting outcomes in patients with covid-19 diagnoses. The results from previous updates, including diagnostic models, are available as supplementary material. A database with the description of each model and validation and its risk of bias assessment can be found on https://www.covprecise.org/.

 Of the 606 prognostic models, 381 were unique, newly developed models for covid-19, and 225 were external validations of existing models in a study other than the model development study. These external validations include external validations of newly developed covid models, as well as prognostic scores predating the covid-19 pandemic. Some models were validated more than once (in different studies, as described below). One hundred and fifty eight (41%) newly developed models were publicly available in a format for use in practice (box 1).

Box 1 **Availability of models in format for use in clinical practice**

Three hundred and eighty one unique prognostic models were developed in the included studies. Eighty (21%) of these models were presented as a model equation including intercept and regression coefficients. Thirty nine (10%) models were only partially presented (eg, intercept or baseline hazard were missing). The remaining 262 (69%) did not provide the underlying model equation.

One hundred and sixty one (42%) were available in a tool to facilitate use in clinical practice (in addition to or instead of a published equation). Sixty models (16%) were presented as a nomogram, 35 (9%) as a web calculator, 30 (8%) as a sum score, nine (2%) as a software object, five (1%) as a decision tree or set of predictions for subgroups, and 22 (6%) in other usable formats (6%).

All these presentation formats make predictions readily available for use in the clinic. However, because most of these prognostic models were at high or uncertain risk of bias, we do not recommend their routine use before they are externally validated, ideally by independent investigators in other data than used for their development.

### Primary datasets

Five hundred and fifty six (92%) developed or validated models used data from a single country (table 1), 39 (6%) used international data, and for 11 (2%) models it was unclear how many (and which) countries contributed data. Three (0.5%) models used simulated data and 21 (3%) used proxy data to estimate covid-19 related risks (eg, Medicare claims data from 2015 to 2016). Most models were intended for use in confirmed covid-19 cases (83%) and a hospital setting (82%). The average patient age ranged from 38 to 84 years, and the proportion of men ranged from 1% to 95%, although this information was often not reported.

**Table 1:** Characteristics of reviewed prediction models for prognosis of coronavirus disease 2019 (covid-19)

	No (%) of models* or median (interquartile range)
**Country†**	
Single country data	556 (92)
China	200 (33)
US	80 (13)
Italy	59 (10)
UK	53 (9)
Spain	40 (7)
South Korea	28 (5)
Mexico	21 (3)
Turkey	15 (2)
Norway	10 (2)
Other single country	50 (8)
International (combined) data	39 (6)
Unknown origin of data	11 (2)
**Type of data used**	
Pandemic data	582 (96)
Proxy (non-covid-19) data	21 (3)
Simulated data	3 (0.5)
**Target setting**	
Patients admitted to hospital	496 (82)
Patient at triage centre or fever clinic	6 (1)
Patients in general practice	3 (0.5)
Other	54 (9)
Unclear	47 (8)
**Target population**	
Confirmed covid-19	502 (83)
Suspected covid-19	46 (8)
Other	25 (4)
Unclear	33 (5)
**Type of model**	
Prognostic model to predict future risk of covid-19 in general population	13 (2)
Prognostic models for outcomes in patients with covid-19	593 (98)
**Analysis done in reviewed study**	
Development only	96 (16)
Development and internal validation	185 (31)
Development and external validation	100 (17)
External validation only	225 (37)
**Sample size**	
Sample size (development)	414 (172-1505)
No of events (development)	74 (36-207)
Sample size (external validation)	314 (127-516)
No of events (external validation)	42 (24-115)

* Analysis unit is a model within a study. Some studies investigated multiple models and some models were investigated in multiple studies (that is, in external validation studies).

† A study that uses development data from one country and validation data from another is classified as international.

Based on the studies that reported study dates, data were collected from December 2019 to October 2020. Some centres provided data to multiple studies and it was unclear how much these datasets overlapped across identified studies. 

The median sample size for model development was 414, with a median number of 74 individuals experiencing the event that was predicted. The mortality risk in patients admitted to hospital ranged from 8% to 46%. This wide variation is partly due to differences in length of follow-up between studies (which was often not reported), local and temporal variation in diagnostic criteria, admission criteria and treatment, as well as selection bias (eg, excluding participants who had neither recovered nor died at the end of the study period).

### Models to predict covid-19 related risks in the general population

We identified 13 newly developed models aiming to predict covid-19 related risks in the general population. Five models predicted hospital admission for covid-19, three predicted mortality, one predicted development of severe covid-19, and four predicted an insufficiently defined covid-19 outcome. Eight of these 13 general population models used proxy outcomes (eg, admission for non-tuberculosis pneumonia, influenza, acute bronchitis, or upper respiratory tract infections instead of hospital admission for covid-19).^20^ The 13 studies reported C indexes between 0.52 and 0.99. Calibration was assessed for only four models, all in one study, which found slight miscalibration.^231^

### Prognostic models for outcomes in patients with diagnosis of covid-19

We identified 593 prognostic models for predicting clinical outcomes in patients with covid-19 (368 developments, 225 external validations). These models were primarily for use in patients admitted to hospital with a proven diagnosis of covid-19 (n=496, 84%), but a specific intended use (ie, when exactly or at which moment in the investigation to use them, and for whom) was often not clearly described. Of these 593 prognostic models, 265 (45%) estimated mortality risk, 84 (14%) predicted progression to a severe or critical disease, and 53 (9%) predicted ICU admission. The remaining 191 studies used other outcomes (single or as part of a composite) including need for intubation, (duration of) mechanical ventilation, oxygen support, acute respiratory distress syndrome, septic shock, cardiovascular complications, (multiple) organ failure, and thrombotic complication, length of hospital stay, recovery, hospital admission or readmission, or length of isolation period. Prediction horizons varied between one day and 60 days but were often unspecified (n=387, 65%). Some studies (n=13, 2%) used proxy outcomes. For example, one study used data from 2015 to 2019 to predict mortality and prolonged assisted mechanical ventilation (as a non-covid-19 proxy outcome).^119^

The studies reported C indexes between 0.49 and 1, with a median of 0.81 (interquartile range 0.75-0.89). The median C index was 0.83 for the mortality models, 0.83 for progression models, and 0.77 for ICU admission models. Researchers showed calibration plots for only 152 of the 593 models (26%, of which 102 at external validation). The calibration results were mixed, with several studies indicating inaccurate risk predictions (examples in Xie et al,^19^ Barda et al,^73^ and Zhang et al^122^). Plots were sometimes constructed in an unclear way, hampering interpretation (examples in Guo et al,^89^ Gong et al,^125^ and Knight et al^147^).

### Risk of bias

**Fig 2:**
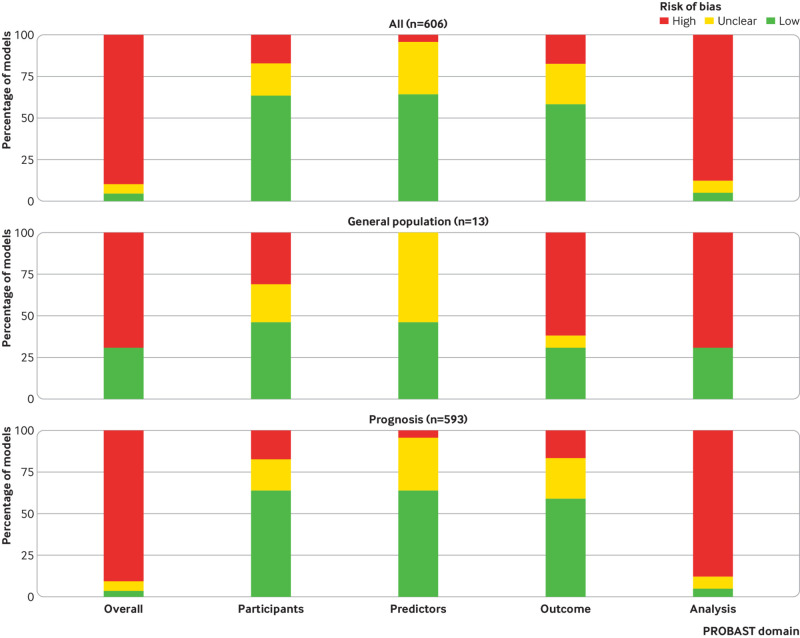
PROBAST (prediction model risk of bias assessment tool) risk of bias for all included models combined (n=606) and broken down per type of analysis

Seven newly developed prognostic models and 22 external validations of prognostic models were at low risk of bias (n=29, 5%). Most newly developed models and external validations were at unclear (n=32, 5%) or high (n=545, 90%) risk of bias according to assessment with PROBAST, which suggests that the predictive performance when used in practice is probably lower than what is reported (fig 2). Figure 2 and box 2 give details on common causes for risk of bias.

Box 2 **Common causes of risk of bias in the reported prediction models of covid-19**

The analysis domain was the most problematic domain: 87% (n=530) of newly developed models and validations were at high risk of bias, compared to 18% (n=107), 4% (n=27), and 17% (n=106) for the participant, predictor, and outcome domains. One hundred and fifty one (25%) models had low risk of bias on all domains except analysis, indicating adequate data collection and study design, but issues that could have been avoided by conducting a better statistical analysis. The most frequent problem was insufficient sample size (n=408, 67%). Small to modest sample sizes and numbers of events (table 1) led to an increased risk of overfitting, particularly if complex modelling strategies were used. Not properly accounting for overfitting or optimism was also common (n=250, 41%). Ninety six models (16%) were neither internally nor externally validated. If done, internal validation was sometimes not correctly executed (ie, not all modelling steps were repeated). Performance statistics from these models are likely optimistic. Moreover, evaluation of discrimination and calibration was often incomplete, or done with inappropriate statistics (n=338, 56%). Calibration was only assessed for 156 models using calibration plots (26%), of which 106 (17%) on external validation data. Inappropriate handling of missing data was common (n=290, 48%). One hundred and twenty seven conducted a complete case analysis (21%), 205 (34%) did not mention how missing data was handled.

**Models to predict covid-19 risk in general population versus prognostic models in patients with covid-19**

The 593 prognostic models for patients with covid-19 were more often at high risk of bias than the 13 general population models (90% (n=536) *v* 69% (n=9)). This difference was mainly due to the analysis domain (88% (n=521) *v* 69% (n=9) at high risk of bias). The median sample size for model development in patients with covid-19 was 397 (71 events), compared to >1.6 million (1867 events) for general population models. The median sample size for external validation was 299 (42 events), compared to >1 million (1303 events) for general population models. Hence, more models had an inadequate sample size for the chosen analysis strategy (69% (n=407) *v* 8% (n=1)), and more were at risk of overfitting and optimism (42% (n=248 *v* 15% (n=2)).

The outcome domain was more problematic for the general population models than for the models for patients with covid-19, with 62% (n=8) versus 17% (n=98) at high risk of bias in this domain. This difference was caused using proxy outcomes (n=8, 62%)—for example, hospital admission due to severe respiratory disease other than covid-19. For the participant and predictor domains, the risk of bias was comparable (fig 2).

**Development and external validation**

External validations were more often at low risk of bias than newly developed models (10%, (n=22/225) *v* 2% (n=7/381)). The statistical analysis domain was the most problematic domain for model development as well as for external validation studies, with 93% (n=353) and 79% (n=177) at high risk of bias for this domain, respectively. The most common causes of high risk of bias were the same for both types (small sample size, inappropriate evaluation of predictive performance, and inappropriate handling of missing data), except for overfitting and optimism, which is not a concern at external validation.

Three hundred and eighty four (63%) of the 606 models and validations had a low risk of bias for the participants domain. One hundred and seven models (18%) had a high risk of bias for the participants domain, which indicates that the participants enrolled in the studies might not be representative of the models’ targeted populations. Unclear reporting on the inclusion of participants led to an unclear risk of bias assessment in 115 models (19%). Three hundred and eighty six models (64%) had a low risk of bias for the predictor domain, while 193 (32%) had an unclear risk of bias and 27 had a high risk of bias (4%). High risk of bias for the predictor domain indicates that predictors were not available at the models’ intended time of use, not clearly defined, or influenced by the outcome measurement. Most studies used outcomes that are easy to assess (eg, all cause death), and hence 353 (58%) were rated at low risk of bias for the outcome domain. Nonetheless, there was cause for concern about bias induced by the outcome measurement in 106 models (17%), for example, due to the use of proxy outcomes (eg, hospital admission for non-covid-19 severe respiratory infections). One hundred and forty seven (24%) had an unclear risk of bias due to opaque or ambiguous reporting. In contrast to the participant, predictor, and outcome domains, the analysis domain was problematic for most of the 606 models and validations. Overall, 530 (87%) were at high risk of bias for the analysis domain, and the reporting was insufficiently clear to assess risk of bias in the analysis in 42 (7%). Only 34 (6%) were at low risk of bias for the analysis domain. 

### Newly developed models at low risk of bias

We found seven newly developed models at low risk of bias (table 2). All had good to excellent discrimination, but calibration varied, highlighting the need of local and temporal recalibration.

**Table 2:** Prediction models for covid-19 with low risk of bias

Study; setting; and outcome	Model	Sample size (total No of participants (No with outcome))	Predictive performance	
			Strongest type of validation reported	C index (95% CI)*
**General population models**				
Clift et al^231^; data from UK, men registered at GP; death with covid-19	Qcovid mortality (male)	Development 3 047 693 (1867); external validation 1 097 268 (744)	External validation, new centres, same country	0.93 (0.92 to 0.94)
Clift et al^231^; data from UK, women registered at GP; death with covid-19	Qcovid mortality (female)	Development 3 035 409 (2517); external validation 1 075 788 (978)	External validation, new centres, same country	0.93 (0.92 to 0.94)
Clift et al^231^; data from UK, men registered at GP; hospital admission for covid-19 or death with covid-19	Qcovid hospital admission (male)	Development 3 047 693 (5962); external validation 1 097 268 (2076)	External validation, new centres, same country	0.86 (0.85 to 0.87)
Clift et al^231^; data from UK, women registered at GP; hospital admission for covid-19 or death with covid-19	Qcovid hospital admission (female)	Development 3 035 409 (4814); external validation 1 075 788 (1627)	External validation, new centres, same country	0.85 (0.84 to 0.86)
**Models for patients with covid-19**				
Carr et al^81^; data from UK, China and Norway; patients admitted to hospital with confirmed covid-19; 14 day ICU admission or death	Carr model	Development 1 276 (389); external validation 6237 (1308)	External validation, new centres, different countries	0.79 (not reported)
Goodacre et al^262^; data from UK; patients with suspected symptoms of covid-19 at the emergency department; 30 day death or organ support	PRIEST score	Development 11 773 (2440); external validation 9118	External validation, new centres, same country	0.80 (0.79 to 0.81)
Gupta et al^268^; data from the UK; hospitalised symptomatic suspected or confirmed cases; ventilatory support, critical care, or in-hospital death	ISARIC4C Deterioration model	Development 66 705 (28 140); external validation 8 239 (3 784)	External validation, new centers, same country	0·77 (0·76 to 0·78)

GP=general practice; ICU=intensive care unit; PRIEST=Pandemic Respiratory Infection Emergency System Triage; ISARIC4C=International Severe Acute Respiratory and Emerging Infections Consortium Coronavirus Clinical Characterisation Consortium; CI= confidence interval.

* Performance from strongest type of validation reported.

The four Qcovid models predict hospital admission and death with covid-19 in the general population in the UK, separately for men and women.^231^ The models use age, ethnic group, deprivation, body mass index, and a range of comorbidities as predictors. The models showed underestimated risks for high risk patients at external validation, which was remedied by recalibrating the model.^231^

The PRIEST score^262^ predicts 30 day death or organ support in patients with suspected or confirmed covid-19 presenting at the emergency department. The triage score is based on NEWS2 (national early warning score 2 consisting of respiratory rate, oxygen saturation, heart rate, systolic blood pressure, temperature, consciousness, air, or supplemental oxygen), age, sex, and performance status (ranging from bed-bound to normal performance). Its external validation in UK emergency departments showed reasonable calibration, but potential heterogeneity in calibration across centres was not examined.

Carr’s model^81^ and the ISARIC 4C deterioration model^268^ predict deterioration in covid-19 patients admitted to hospital. The composite outcomes for both models included ICU admission and death, while the ISARIC 4C model also adds ventilatory support. Both models had comparable performance but included different predictors, all typically available at admission. Carr and colleagues supplemented NEWS2 with age, laboratory and physiological parameters (supplemental oxygen flow rate, urea, oxygen saturation, C reactive protein, estimated glomerular filtration rate, neutrophil count, neutrophil-lymphocyte ratio). Gupta and colleagues^268^ developed a model including age, sex, nosocomial infection, Glasgow coma scale score, peripheral oxygen saturation at admission, breathing room air or oxygen therapy, respiratory rate, urea concentration, C reactive protein concentration, lymphocyte count, and presence of radiographic chest infiltrates. Carr’s model was validated internationally^81^ and the ISARIC4C Deterioration model was validated regionally within the UK.^268^ For both models, calibration varied across settings.

### External validations at low risk of bias

We identified 225 external validations in dedicated (ie, not combined with the development of the model) external validation studies. Only 22 were low risk of bias, although all 22 validations came from the same study using single-centre UK data (table 3).^269^ This validation study included 411 patients, of which 180 experienced a deterioration in health, and 115 died. In this study, the Carr model and NEWS2 performed best to predict deterioration, while the Xie model and REMS performed best to predict mortality. Both the Carr model (a preprint version that differs slightly from the Carr model reported above) and the Xie model showed slight miscalibration.

**Table 3:** External validations with low risk of bias from Gupta et al^269^

Outcome	Model	C index (95% CI)
1 day deterioration	NEWS2^431^	0.78 (0.73 to 0.83)
10 day deterioration	Ji^94^	0.56 (0.50 to 0.62)
14 day deterioration	Carr (pre-print*)^432^	0.78 (0.74 to 0.82)
14 day deterioration	Carr (preprint threshold*)	0.76 (0.71 to 0.81)
14 day deterioration	Guo^89^	0.67 (0.61 to 0.73)
In-hospital deterioration	Zhang (poor†)^122^	0.74 (0.69 to 0.79)
In-hospital deterioration	Galloway^139^	0.72 (0.68 to 0.77)
In-hospital deterioration	TACTIC^433^	0.70 (0.65 to 0.75)
In-hospital deterioration	Colombi^85^	0.69 (0.63 to 0.74)
In-hospital deterioration	Huang^66^	0.67 (0.1 to 0.73)
In-hospital deterioration	Shi^43^	0.61 (0.56 to 0.66)
In-hospital deterioration	MEWS^434^	0.60 (0.54 to 0.65)
12 day mortality	Lu^26^	0.72 (0.67 to 0.76)
30 day mortality	CURB-65^435^	0.75 (0.70 to 0.80)
30 day mortality	Bello-Chavolla^76^	0.66 (0.60 to 0.72)
In-hospital mortality	REMS^436^	0.76 (0.71 to 0.81)
In-hospital mortality	Xie^19^	0.76 (0.69 to 0.82)
In-hospital mortality	Hu^91^	0.74 (0.68 to 0.79)
In-hospital mortality	Caramelo^25^	0.71 (0.66 to 0.76)
In-hospital mortality	Zhang (death†)^122^	0.70 (0.65 to 0.76)
In-hospital mortality	qSOFA^437^	0.60 (0.55 to 0.65)
In-hospital mortality	Yan^28^	0.58 (0.49 to 0.67)

NEWS2=national early warning score 2; TACTIC=therapeutic study in pre-ICU patients admitted with covid-19; MEWS=modified early warning score; REMS=rapid emergency medicine score; qSOFA=quick sequential (sepsis-related) organ failure assessment; CURB-65=confusion, urea, respiratory rate, blood pressure plus age of at least 65 years; CI=confidence interval.

* Preprint of the study by Carr et al^432^ contains a model with and without a threshold. Both were validated separately by Gupta et al.

† Preprint of the study by Zhang et al^122^ contains a model for poor outcomes (defined originally as developing ARDS, need for intubation or extracorporeal membrane oxygenation support, ICU admission and death), and a model for death. Both were validated separately.

NEWS2 and REMS were also validated in other dedicated validation studies. NEWS2 obtained C indexes between 0.65 and 0.90.^141^ ^203^ ^214^ ^233^ ^245^ ^280^ ^281^ ^303^ ^319^ ^340^ REMS obtained C indexes between 0.74 and 0.88.^91^ ^233^ ^319^ These studies were too heterogeneous and biased to meta-analyse: they used varying outcome definitions (mortality, ICU admission, various composites, with time horizons varying from 1 to 30 days), from different populations (Italy, UK, Norway, China), and were at high or unclear risk of bias.

## Discussion

In this systematic review of prognostic prediction models related to the covid-19 pandemic, we identified and critically appraised 606 models described in 310 studies. These prognostic models can be divided into models to predict the risk of developing covid-19 or having an adverse disease course in the general population (n=13), and models to support the prognosis of patients with covid-19 (n=593). Most studies reported moderate to excellent predictive performance, but only seven newly developed models and 22 external validations of existing models were at low risk of bias. From these, we identified eight models, all developed for prognosis of covid-19, with adequate performance and low risk of bias at model development (four Qcovid models,^231^ the PRIEST model,^262^ the ISARIC4C deterioration model,^268^ and Carr’s model^81^) or external validation (Xie’s model^19^ ^269^). We suggest that these models should be further validated within other datasets and settings, and ideally by independent investigators, to investigate which models maintain a robust performance over time and in varying settings.

Most of the 606 models were appraised to have high or uncertain risk of bias owing to a combination of poor reporting and poor methodological conduct. Often, the available sample sizes and number of events for the outcomes of interest were limited. This problem is well known when building prediction models and increases the risk of overfitting the model.^438^ Other common causes for bias were not adequately accounting for missing data, using techniques that do not account for optimism in performance estimates, ignoring model calibration, and inappropriate model validation. A high risk of bias implies that the performance of these models in new samples will probably be worse than that reported by the researchers. Therefore, the estimated C indexes, often indicating near perfect discrimination, are probably optimistic. For most of these models, no independent external validations with a low risk of bias were performed, even though most were publicly available in a format usable in clinical practice.

### Challenges and opportunities

The main aim of prediction models is to support medical decision making in individual patients. Therefore, it is vital to identify a target setting in which predictions serve a clinical need (eg, emergency department, intensive care unit, general practice, symptom monitoring app in the general population), and a representative dataset from that setting (preferably comprising consecutive patients) on which the prediction model can be developed and validated. This clinical setting and patient characteristics should be described in detail (including timing within the disease course, the severity of disease at the moment of prediction, and the comorbidity), so that readers and clinicians are able to understand if the proposed model is suited for their population. However, the studies included in our systematic review often lacked an adequate description of the target setting and study population, which leaves users of these models in doubt about the models’ applicability. Although we recognise that the earlier studies were done under severe time constraints, we recommend that researchers adhere to the TRIPOD reporting guideline^15^ ^16^ to improve the description of their study population and guide their modelling choices. TRIPOD translations (eg, in Chinese) are also available at https://www.tripod-statement.org. A better description of the study population could also help us understand the observed variability in the reported outcomes across studies, such as covid-19 related mortality. The variability in mortality could be related to differences in included patients (eg, age, comorbidities) but also in interventions for covid-19. 

In this living review, inadequate sample size to build a robust model or to obtain reliable performance statistics was one of the most prevalent shortcomings. We recommend researchers should make use of formulas and software that have been made available in recent years to calculate the required sample size to build or externally validate models.^439^ ^440^ ^441^ ^442^ The current review also identified that ignoring missing data and performing a complete case analysis is still very common. As this leads to reduced precision and can introduce bias in the estimated model, we recommend researchers address missing data using appropriate techniques before developing or validating a model.^443^ ^444^ When creating a new prediction model, we recommend building on previous literature and expert opinion to select predictors, rather than selecting predictors purely based on data.^17^ This recommendation is especially important for datasets with limited sample size.^445^ To temper optimism in estimated performance, several internal validation strategies can be used—for example, bootstrapping.^17^ ^446^ We also recommend that researchers should evaluate model performance in terms of correspondence between predicted and observed risk, preferably using flexible calibration plots^17^ ^447^ in addition to discrimination. 

Covid-19 prediction will often not present as a simple binary classification task. Complexities in the data should be handled appropriately. For example, a prediction horizon should be specified for prognostic outcomes (eg, 30 day mortality). If study participants neither recovered nor died within that period, their data should not be excluded from analysis, which some reviewed studies have done. Instead, an appropriate time-to-event analysis should be considered to allow for administrative censoring.^17^ Censoring for other reasons, for instance because of quick recovery and loss to follow-up of patients who are no longer at risk of death from covid-19, could necessitate analysis in a competing risk framework.^448^

A prediction model applied in a new healthcare setting or country often produces predictions that are miscalibrated^447^ and might need to be updated before it can safely be applied in that new setting.^17^ This requires data from patients with covid-19 to be available from that setting. In addition to updating predictions in their local setting, individual participant data from multiple countries and healthcare systems might allow better understanding of the generalisability and implementation of prediction models across different settings and populations. This approach could greatly improve the applicability and robustness of prediction models in routine care.^446^ ^449^ ^450^ ^451^ ^452^

The covid-19 pandemic has been characterised by an unprecedented speed of data accumulation worldwide. Unfortunately, much of the work done to analyse all these data has been ill informed and disjointed. As a result, we have hundreds of similar models, and very few independent validation studies comparing their performance on the same data. To leverage the full potential of prediction models in emerging pandemics and quickly identify useful models, international and interdisciplinary collaboration in terms of data acquisition, model building, model validation, and systematic review is crucial.

### Study limitations

With new publications on covid-19 related prediction models entering the medical literature in unprecedented numbers and at unprecedented speed, this systematic review cannot be viewed as an up-to-date list of all currently available covid-19 related prediction models. It does provide a comprehensive overview of all prognostic model developments and validations from the first year of the pandemic up to 17 February 2021. Also, 69 of the studies we reviewed were only available as preprints. Some of these studies might enter the official medical literature in an improved version, after peer review. We reassessed peer reviewed publications of preprints included in previous updates that have been published before the current update. We also found other prediction models have been used in clinical practice without scientific publications,^453^ and web risk calculators launched for use while the scientific manuscript is still under review (and unavailable on request).^454^ These unpublished models naturally fall outside the scope of this review of the literature. As we have argued extensively elsewhere,^455^ transparent reporting that enables validation by independent researchers is key for predictive analytics, and clinical guidelines should only recommend publicly available and verifiable algorithms.

### Implications for practice

This living review has identified a handful of models developed specifically for covid-19 prognosis with good predictive performance at external validation, and with model development or external validation at low risk of bias. The Qcovid models^231^ were built to prognosticate hospital admission and mortality risks in the general population. The PRIEST model was proposed to triage patients at the emergency department.^262^ The ISARIC4C Deterioration model,^268^ Carr model,^81^ and Xie model^19^ ^269^ were developed to predict adverse outcomes in hospitalised patients (ventilatory support, critical care or death, ICU admission or death, and death, respectively). Since the search date, these models have been validated temporally and geographically, which demonstrated that care should be taken when using these models in policy or clinical practice.^231^ ^268^ ^456^ ^457^ ^458^ ^459^ ^460^ Differences between healthcare systems, fluctuations in infection rates, virus mutations, differences in vaccination status, varying testing criteria, and changes in patient management and treatment can lead to miscalibration in more recent or local data. Hence, future studies should focus on validating and comparing these prediction models with low risk of bias.^17^ External validations should not only assess discrimination, but also calibration and clinical usefulness (net benefit),^447^ ^452^ ^461^ in large studies^439^ ^440^ ^442^ ^462^ ^463^ using an appropriate design.

Many prognostic models have been developed for prognostication in a hospital setting. Updating an available model to accommodate temporal or regional differences or extending an existing model with new predictors requires less data and provides generally more robust predictions than developing a new prognostic model.^17^ New variants could vary in contagiousness and severity, and vaccination and waning immunity might alter individual risks. Consequently, even updated models could become outdated. These changes would primarily affect calibration (ie, absolute risk estimates might be too high or too low), while the discrimination between low and high risk patients could be less affected. Miscalibration is especially concerning for general population models. Models that focus on patients seeking care and adjust risk estimates for symptoms and severity markers might be more robust, but this hypothesis remains to be confirmed empirically.

Although many models exist to predict outcomes at the emergency department or at hospital admission, few are suited for patients with symptoms attending primary care, or for patients admitted to the ICU. In addition, the models reviewed so far focus on the covid-19 diagnosis or assess the risk of mortality or deterioration, whereas long term morbidity and functional outcomes remain understudied and could be a target outcome of interest in future studies developing prediction models.^464^ ^465^

This review of prediction models developed in the first year of the covid-19 pandemic found most models at unclear or high risk of bias. Whereas many external validations were done, most were at high risk of bias and most models developed specifically for covid-19 were not validated independently. This oversupply of insufficiently validated models is not useful for clinical practice. Moreover, the urgency of diagnostic and prognostic models to assist in quick and efficient triage of patients in an emergent pandemic might encourage clinicians and policymakers to prematurely implement prediction models without sufficient documentation and validation. Inaccurate models could even cause more harm than good.^461^ By pointing to the most important methodological challenges and issues in design and reporting, we hope to have provided a useful starting point for future studies and future epidemics. 

### Conclusion

Several prognostic models for covid-19 are currently available and most report moderate to excellent discrimination. However, many of these models are at high or unclear risk of bias, mainly because of model overfitting, inappropriate model evaluation (eg, calibration ignored), and inappropriate handling of missing data. Therefore, their performance estimates are probably optimistic and might not be representative for the target population. We found that the Qcovid models can be used for risk stratification in the general population, while the PRIEST model, ISARIC4C Deterioration model, Carr’s model, and Xie’s model are suitable for prognostication in a hospital setting. The performance of these models is likely to vary over time and differ between regions, necessitating further validation and potentially updating before implementation. For details of the reviewed models, see https://www.covprecise.org/. Sharing data and expertise for the validation and updating of covid-19 related prediction models is still needed.

**What is already known on this topic**

Recurrent peaks in covid-19 incidence have put a strain on healthcare systems worldwide; a need exists for efficient early risk stratification in the general population, and for prognosis of covid-19 in patients with confirmed disease
Viral nucleic acid testing, chest computed tomography imaging, and antigen tests are standard methods for diagnosing covid-19, and their availability has made covid-19 diagnostic models less relevant
Earlier updates of this living review could not find models at low risk of bias

**What this study adds**

Of models with a low risk of bias, four identify patients at risk in the general population; one assists in patient triage at the emergency department; and three estimate prognosis in patients admitted to hospital with covid-19
Calibration of these models is likely to vary over time and across settings
There is an oversupply of models and external validations at high risk of bias, raising concern that predictions could be unreliable when these models are applied in dailly practice

## Data Availability

The study protocol is available online at https://osf.io/ehc47/. Detailed extracted data on all included studies are available on https://www.covprecise.org/.

## Web extra

Extra material supplied by authors

Web appendix: Supplementary material

## Notes

Contributors: LW conceived the study. LW and MvS designed the study. LW, MvS, and BVC screened titles and abstracts for inclusion. LW, BVC, GSC, TPAD, MCH, GH, KGMM, RDR, ES, LJMS, EWS, KIES, CW, AL, JM, TT, JAD, KL, JBR, LH, CS, MS, MCH, NS, NK, SMJvK, JCS, PD, CLAN, RW, GPM, IT, JYV, DLD, JW, FSvR, PH, VMTdJ, BCTvB, ICCvdH, DJM, MK, BL, EA, SG, BA, JH, KJ, SG, KR, JE, MH, VB, and MvS extracted and analysed data. MDV helped interpret the findings on deep learning studies and MMJB, LH, and MCH assisted in the interpretation from a clinical viewpoint. RS and FSvR offered technical and administrative support. LW wrote the first draft, which all authors revised for critical content. All authors approved the final manuscript. LW and MvS are the guarantors. The guarantors had full access to all the data in the study, take responsibility for the integrity of the data and the accuracy of the data analysis, and had final responsibility for the decision to submit for publication. The corresponding author attests that all listed authors meet authorship criteria and that no others meeting the criteria have been omitted.

The lead authors affirm that the manuscript is an honest, accurate, and transparent account of the study being reported; that no important aspects of the study have been omitted; and that any discrepancies from the study as planned have been explained.

Dissemination to participants and related patient and public communities: The authors and patient partners will distribute this information through their institutions and on social media to provide an opportunity for public dialogue and as an example of how what we learn as the result of a new disease changes and improves over time. The study protocol is available online at https://osf.io/ehc47/.

Provenance and peer review: Not commissioned; externally peer reviewed.

## References

[1] Dong E Du H Gardner L . An interactive web-based dashboard to track covid-19 in real time. Lancet Infect Dis 2020:S1473-3099(20)30120-1. 10.1016/S1473-3099(20)30120-1 32087114 PMC7159018

[2] Arabi YM Murthy S Webb S . covid-19: a novel coronavirus and a novel challenge for critical care. Intensive Care Med 2020. 10.1007/s00134-020-05955-1. 32125458 PMC7080134

[3] Grasselli G Pesenti A Cecconi M . Critical care utilization for the covid-19 outbreak in Lombardy, Italy: early experience and forecast during an emergency response. JAMA 2020. 10.1001/jama.2020.4031 32167538

[4] Xie J Tong Z Guan X Du B Qiu H Slutsky AS . Critical care crisis and some recommendations during the covid-19 epidemic in China. Intensive Care Med 2020. 10.1007/s00134-020-05979-7 32123994 PMC7080165

[5] World Health Organization. Essential health services face continued disruption during COVID-19 pandemic 2022. https://www.who.int/news/item/07-02-2022-essential-health-services-face-continued-disruption-during-covid-19-pandemic.

[6] Ritchie H, Mathieu E, Rodés-Guirao L, et al. Coronavirus Pandemic (COVID-19) 2020. https://ourworldindata.org/coronavirus accessed 07/03/2022.

[7] Telenti A Arvin A Corey L . After the pandemic: perspectives on the future trajectory of COVID-19. Nature 2021;596:495-504. 10.1038/s41586-021-03792-w. 34237771

[8] Katzourakis A . COVID-19: endemic doesn’t mean harmless. Nature 2022;601:485. 10.1038/d41586-022-00155-x. 35075305

[9] Ipekci AM Buitrago-Garcia D Meili KW . Outbreaks of publications about emerging infectious diseases: the case of SARS-CoV-2 and Zika virus. BMC Med Res Methodol 2021;21:50. 10.1186/s12874-021-01244-7. 33706715 PMC7948668

[10] Institute of Social and Preventive Medicine. Living evidence on covid-19 2020. https://ispmbern.github.io/covid-19/living-review/index.html.

[11] Thomas J, Brunton J, Graziosi S. EPPI-Reviewer 4.0: software for research synthesis [program]. EPPI-Centre Software. London: Social Science Research Unit, Institute of Education, University of London, 2010.

[12] Moons KG de Groot JA Bouwmeester W . Critical appraisal and data extraction for systematic reviews of prediction modelling studies: the CHARMS checklist. PLoS Med 2014;11:e1001744. 10.1371/journal.pmed.1001744 25314315 PMC4196729

[13] Moons KGM Wolff RF Riley RD . PROBAST: a tool to assess risk of bias and applicability of prediction model studies: explanation and elaboration. Ann Intern Med 2019;170:W1-33. 10.7326/M18-1377 30596876

[14] Wolff RF Moons KGM Riley RD PROBAST Group† . PROBAST: A Tool to Assess the Risk of Bias and Applicability of Prediction Model Studies. Ann Intern Med 2019;170:51-8. 10.7326/M18-1376. 30596875

[15] Moons KGM Altman DG Reitsma JB . Transparent Reporting of a multivariable prediction model for Individual Prognosis or Diagnosis (TRIPOD): explanation and elaboration. Ann Intern Med 2015;162:W1-73. 10.7326/M14-0698 25560730

[16] Collins GS Reitsma JB Altman DG Moons KG . Transparent reporting of a multivariable prediction model for individual prognosis or diagnosis (TRIPOD): the TRIPOD statement. BMJ 2015;350:g7594. 10.1136/bmj.g7594. 25569120

[17] Steyerberg EW . Clinical prediction models: a practical approach to development, validation, and updating. Springer US, 2019 10.1007/978-3-030-16399-0 .

[18] Liberati A Altman DG Tetzlaff J . The PRISMA statement for reporting systematic reviews and meta-analyses of studies that evaluate health care interventions: explanation and elaboration. PLoS Med 2009;6:e1000100. 10.1371/journal.pmed.1000100 19621070 PMC2707010

[19] Xie J, Hungerford D, Chen H, et al. Development and external validation of a prognostic multivariable model on admission for hospitalized patients with covid-19. medRxiv [Preprint] 2020. 10.1101/2020.03.28.20045997

[20] DeCaprio D Gartner J McCall CJ . Building a covid-19 vulnerability index. J Med Artificial Intel 2020;3. 10.21037/jmai-20-47.

[21] Fang C, Bai S, Chen Q, et al. Predicting covid-19 malignant progression with AI techniques. medRxiv [Preprint] 2020. 10.1101/2020.03.20.20037325

[22] Feng C, Huang Z, Wang L, et al. A novel triage tool of artificial intelligence assisted diagnosis aid system for suspected covid-19 pneumonia in fever clinics. medRxiv [Preprint] 2020. 10.1101/2020.03.19.20039099 PMC7940949 33708828

[23] Jin C, Chen W, Cao Y, et al. Development and evaluation of an AI system for covid-19 diagnosis. medRxiv [Preprint] 2020. 10.1101/2020.03.20.20039834 PMC7547659 33037212

[24] Meng Z, Wang M, Song H, et al. Development and utilization of an intelligent application for aiding covid-19 diagnosis. medRxiv [Preprint] 2020. 10.1101/2020.03.18.20035816

[25] Caramelo F, Ferreira N, Oliveiros B. Estimation of risk factors for covid-19 mortality - preliminary results. medRxiv [Preprint] 2020. 10.1101/2020.02.24.20027268

[26] Lu J, Hu S, Fan R, et al. ACP risk grade: a simple mortality index for patients with confirmed or suspected severe acute respiratory syndrome coronavirus 2 disease (covid-19) during the early stage of outbreak in Wuhan, China. medRxiv [Preprint] 2020. 10.1101/2020.02.20.20025510

[27] Qi X, Jiang Z, YU Q, et al. Machine learning-based CT radiomics model for predicting hospital stay in patients with pneumonia associated with SARS-CoV-2 infection: a multicenter study. medRxiv [Preprint] 2020. 10.1101/2020.02.29.20029603 PMC7396749 32793703

[28] Yan L, Zhang H-T, Xiao Y, et al. Prediction of criticality in patients with severe Covid-19 infection using three clinical features: a machine learning-based prognostic model with clinical data in Wuhan. medRxiv [Preprint] 2020. 10.1101/2020.02.27.20028027

[29] Yuan M Yin W Tao Z Tan W Hu Y . Association of radiologic findings with mortality of patients infected with 2019 novel coronavirus in Wuhan, China. PLoS One 2020;15:e0230548. 10.1371/journal.pone.0230548 32191764 PMC7082074

[30] Ying S, Zheng S, Li L, et al. Deep learning enables accurate diagnosis of novel coronavirus (covid-19) with CT images. medRxiv [Preprint] 2020. 10.1101/2020.02.23.20026930 PMC8851430 33705321

[31] Yu H, Shao J, Guo Y, et al. Data-driven discovery of clinical routes for severity detection in covid-19 pediatric cases. medRxiv [Preprint] 2020. 10.1101/2020.03.09.20032219

[32] Gozes O, Frid-Adar M, Greenspan H, et al. Rapid AI development cycle for the coronavirus (covid-19) pandemic: initial results for automated detection & patient monitoring using deep learning CT image analysis. arXiv e-prints [Preprint] 2020. https://ui.adsabs.harvard.edu/abs/2020arXiv200305037G

[33] Chen J, Wu L, Zhang J, et al. Deep learning-based model for detecting 2019 novel coronavirus pneumonia on high-resolution computed tomography: a prospective study. medRxiv [Preprint] 2020. 10.1101/2020.02.25.20021568 PMC7645624 33154542

[34] Xu X, Jiang X, Ma C, et al. Deep learning system to screen coronavirus disease 2019 pneumonia. arXiv e-prints [Preprint] 2020. https://ui.adsabs.harvard.edu/abs/2020arXiv200209334X

[35] Shan F, Gao Y, Wang J, et al. Lung infection quantification of covid-19 in CT images with deep learning. arXiv e-prints 2020. https://ui.adsabs.harvard.edu/abs/2020arXiv200304655S

[36] Wang S, Kang B, Ma J, et al. A deep learning algorithm using CT images to screen for corona virus disease (covid-19). medRxiv [Preprint] 2020. 10.1101/2020.02.14.20023028 PMC7904034 33629156

[37] Song C-Y, Xu J, He J-Q, et al. covid-19 early warning score: a multi-parameter screening tool to identify highly suspected patients. medRxiv [Preprint] 2020. 10.1101/2020.03.05.20031906

[38] Barstugan M, Ozkaya U, Ozturk S. Coronavirus (covid-19) classification using CT images by machine learning methods. arXiv e-prints [Preprint] 2020. https://ui.adsabs.harvard.edu/abs/2020arXiv200309424B

[39] Jin S, Wang B, Xu H, et al. AI-assisted CT imaging analysis for covid-19 screening: building and deploying a medical AI system in four weeks. medRxiv [Preprint] 2020. 10.1101/2020.03.19.20039354 PMC7654325 33199977

[40] Li L Qin L Xu Z . Artificial intelligence distinguishes covid-19 from community acquired pneumonia on chest CT. Radiology 2020:200905. 32191588

[41] Lopez-Rincon A, Tonda A, Mendoza-Maldonado L, et al. Accurate identification of SARS-CoV-2 from viral genome sequences using deep learning. bioRxiv [Preprint] 2020. 10.1101/2020.03.13.990242

[42] Shi F, Xia L, Shan F, et al. Large-scale screening of covid-19 from community acquired pneumonia using infection size-aware classification. arXiv [Preprint] 2020. https://arxiv.org/abs/2003.09860 10.1088/1361-6560/abe838 33729998

[43] Shi Y Yu X Zhao H Wang H Zhao R Sheng J . Host susceptibility to severe covid-19 and establishment of a host risk score: findings of 487 cases outside Wuhan. Crit Care 2020;24:108. 10.1186/s13054-020-2833-7 32188484 PMC7081524

[44] Zheng C, Deng X, Fu Q, et al. Deep learning-based detection for covid-19 from chest CT using weak label. medRxiv [Preprint] 2020. 10.1101/2020.03.12.20027185

[45] Chowdhury MEH, Rahman T, Khandakar A, et al. Can AI help in screening Viral and covid-19 pneumonia? arXiv e-prints [Preprint] 2020. https://ui.adsabs.harvard.edu/abs/2020arXiv200313145C.

[46] Sun Y Koh V Marimuthu K . Epidemiological and clinical predictors of covid-19. Clin Infect Dis 2020;ciaa322. 32211755 10.1093/cid/ciaa322 PMC7542554

[47] Martin A, Nateqi J, Gruarin S, et al. An artificial intelligence-based first-line defence against covid-19: digitally screening citizens for risks via a chatbot. bioRxiv [Preprint] 2020. 10.1101/2020.03.25.008805 PMC7643065 33149198

[48] Wang S, Zha Y, Li W, et al. A fully automatic deep learning system for covid-19 diagnostic and prognostic analysis. medRxiv [Preprint] 2020. 10.1101/2020.03.24.20042317 PMC7243395 32444412

[49] Wang Z, Weng J, Li Z, et al. Development and validation of a diagnostic nomogram to predict covid-19 pneumonia. medRxiv [Preprint] 2020. 10.1101/2020.04.03.20052068

[50] Sarkar J, Chakrabarti P. A machine learning model reveals older age and delayed hospitalization as predictors of mortality in patients with covid-19. medRxiv [Preprint] 2020. 10.1101/2020.03.25.20043331

[51] Wu J, Zhang P, Zhang L, et al. Rapid and accurate identification of covid-19 infection through machine learning based on clinical available blood test results. medRxiv [Preprint] 2020. 10.1101/2020.04.02.20051136

[52] Zhou Y, Yang Z, Guo Y, et al. A new predictor of disease severity in patients with covid-19 in Wuhan, China. medRxiv [Preprint] 2020. 10.1101/2020.03.24.20042119

[53] Abbas A, Abdelsamea M, Gaber M. Classification of covid-19 in chest x-ray images using DeTraC deep convolutional neural network. medRxiv [Preprint] 2020. 10.1101/2020.03.30.20047456 PMC7474514 34764548

[54] Apostolopoulos ID Mpesiana TA . Covid-19: automatic detection from X-ray images utilizing transfer learning with convolutional neural networks. Physical and Engineering Sciences in Medicine, 2020. 10.1007/s13246-020-00865-4. PMC7118364 32524445

[55] Bukhari SUK, Bukhari SSK, Syed A, et al. The diagnostic evaluation of Convolutional Neural Network (CNN) for the assessment of chest X-ray of patients infected with covid-19. medRxiv [Preprint] 2020. 10.1101/2020.03.26.20044610

[56] Chaganti S, Balachandran A, Chabin G, et al. Quantification of tomographic patterns associated with covid-19 from chest CT. arXiv e-prints [Preprint] 2020. https://ui.adsabs.harvard.edu/abs/2020arXiv200401279C. 10.1148/ryai.2020200048 PMC7392373 33928255

[57] Fu M, Yi S-L, Zeng Y, et al. Deep learning-based recognizing covid-19 and other common infectious diseases of the lung by chest CT scan images. medRxiv [Preprint] 2020. 10.1101/2020.03.28.20046045

[58] Gozes O, Frid-Adar M, Sagie N, et al. Coronavirus detection and analysis on chest CT with deep learning. arXiv e-prints [Preprint] 2020. https://ui.adsabs.harvard.edu/abs/2020arXiv200402640G.

[59] Imran A, Posokhova I, Qureshi HN, et al. AI4covid-19: AI enabled preliminary diagnosis for covid-19 from cough samples via an app. arXiv e-prints [Preprint] 2020. https://ui.adsabs.harvard.edu/abs/2020arXiv200401275I. 10.1016/j.imu.2020.100378 PMC7318970 32839734

[60] Li K Fang Y Li W . CT image visual quantitative evaluation and clinical classification of coronavirus disease (covid-19). Eur Radiol 2020; 10.1007/s00330-020-06817-6 32215691 PMC7095246

[61] Li X, Li C, Zhu D. covid-MobileXpert: on-device covid-19 screening using snapshots of chest x-ray. arXiv e-prints [Preprint] 2020. https://ui.adsabs.harvard.edu/abs/2020arXiv200403042L.

[62] Mahdy LN, Ezzat KA, Elmousalami HH, et al. Automatic x-ray covid-19 lung image classification system based on multi-level thresholding and support vector machine. medRxiv [Preprint] 2020. 10.1101/2020.03.30.20047787

[63] Tang Z, Zhao W, Xie X, et al. Severity assessment of coronavirus disease 2019 (covid-19) using quantitative features from chest CT images. arXiv e-prints [Preprint] 2020. https://ui.adsabs.harvard.edu/abs/2020arXiv200311988T.

[64] Zhang J, Xie Y, Li Y, et al. covid-19 Screening on Chest X-ray Images Using Deep Learning based Anomaly Detection. arXiv e-prints 2020. https://ui.adsabs.harvard.edu/abs/2020arXiv200312338Z.

[65] Zhou M, Chen Y, Wang D, et al. Improved deep learning model for differentiating novel coronavirus pneumonia and influenza pneumonia. medRxiv [Preprint] 2020. 10.1101/2020.03.24.20043117

[66] Huang H Cai S Li Y . Prognostic Factors for COVID-19 Pneumonia Progression to Severe Symptoms Based on Earlier Clinical Features: A Retrospective Analysis. Front Med (Lausanne) 2020;7:557453. 10.3389/fmed.2020.557453. 33123541 PMC7571455

[67] Pourhomayoun M Shakibi M . Predicting mortality risk in patients with COVID-19 using machine learning to help medical decision-making. Smart Health (Amst) 2021;20:100178. 10.1016/j.smhl.2020.100178. 33521226 PMC7832156

[68] Zeng L, Li J, Liao M, et al. Risk assessment of progression to severe conditions for patients with covid-19 pneumonia: a single-center retrospective study. medRxiv [Preprint] 2020. 10.1101/2020.03.25.20043166

[69] Al-Najjar H Al-Rousan N . A classifier prediction model to predict the status of coronavirus covid-19 patients in South Korea. Eur Rev Med Pharmacol Sci 2020;24:3400-3. 10.26355/eurrev_202003_20709 32271458

[70] Angelov P, Soares E. Explainable-by-design approach for covid-19 classification via CT-scan. medRxiv [Preprint] 2020. 10.1101/2020.04.24.20078584.

[71] Arpan M, Surya K, Harish R, et al. CovidAID: covid-19 Detection Using Chest X-Ray. ArXiv e-prints [Preprint] 2020

[72] Bai HX Wang R Xiong Z . AI augmentation of radiologist performance in distinguishing covid-19 from pneumonia of other etiology on chest CT. Radiology 2020;201491. 32339081 10.1148/radiol.2020201491 PMC7233483

[73] Barda N Riesel D Akriv A . Developing a COVID-19 mortality risk prediction model when individual-level data are not available. Nat Commun 2020;11:4439. 10.1038/s41467-020-18297-9. 32895375 PMC7477233

[74] Bassi PRAS, Attux R. A deep convolutional neural network for covid-19 detection using chest x-rays. ArXiv e-prints [Preprint] 2020

[75] Batista AfdM. Miraglia JL, Donato THR, et al. covid-19 diagnosis prediction in emergency care patients: a machine learning approach. medRxiv [Preprint] 2020 10.1101/2020.04.04.20052092.

[76] Bello-Chavolla OY Bahena-López JP Antonio-Villa NE . Predicting mortality due to SARS-CoV-2: A mechanistic score relating obesity and diabetes to covid-19 outcomes in Mexico. J Clin Endocrinol Metab 2020;105:dgaa346. 10.1210/clinem/dgaa346 32474598 PMC7313944

[77] Benchoufi M, Bokobza J, Anthony C, et al. Lung injury in patients with or suspected covid-19: a comparison between lung ultrasound and chest CT-scanner severity assessments, an observational study. MedRxiv [Preprint] 2020 10.1101/2020.04.24.20069633.

[78] Borghesi A Maroldi R . covid-19 outbreak in Italy: experimental chest X-ray scoring system for quantifying and monitoring disease progression. Radiol Med 2020;125:509-13. 10.1007/s11547-020-01200-3 32358689 PMC7194501

[79] Born J, Brandle G, Cossio M, et al. Pocovid-Net: Automatic detection of covid-19 from a new lung ultrasound imaging dataset (POCUS). ArXiv e-prints [Preprint] 2020.

[80] Brinati D Campagner A Ferrari D Locatelli M Banfi G Cabitza F . Detection of COVID-19 Infection from Routine Blood Exams with Machine Learning: A Feasibility Study. J Med Syst 2020;44:135. 10.1007/s10916-020-01597-4. 32607737 PMC7326624

[81] Carr E Bendayan R Bean D . Evaluation and improvement of the National Early Warning Score (NEWS2) for COVID-19: a multi-hospital study. BMC Med 2021;19:23. 10.1186/s12916-020-01893-3. 33472631 PMC7817348

[82] Castiglioni I, Ippolito D, Interlenghi M, et al. Artificial intelligence applied on chest X-ray can aid in the diagnosis of covid-19 infection: a first experience from Lombardy. medRxiv [Preprint] 2020. 10.1101/2020.04.08.20040907.

[83] Chassagnon G Vakalopoulou M Battistella E . AI-driven quantification, staging and outcome prediction of COVID-19 pneumonia. Med Image Anal 2021;67:101860. 10.1016/j.media.2020.101860. 33171345 PMC7558247

[84] Chen X Tang Y Mo Y . A diagnostic model for coronavirus disease 2019 (covid-19) based on radiological semantic and clinical features: a multi-center study. Eur Radiol 2020. 10.1007/s00330-020-06829-2 32300971 PMC7160614

[85] Colombi D Bodini FC Petrini M . Well-aerated lung on admitting chest CT to predict adverse outcome in covid-19 pneumonia. Radiology 2020;201433. 10.1148/radiol.2020201433 32301647 PMC7233411

[86] Das AK Mishra S Saraswathy Gopalan S . Predicting CoVID-19 community mortality risk using machine learning and development of an online prognostic tool. PeerJ 2020;8:e10083. 10.7717/peerj.10083. 33062451 PMC7528809

[87] Diaz-Quijano FA, Silva JMNd, Ganem F, et al. A model to predict SARS-CoV-2 infection based on the first three-month surveillance data in Brazil. medRxiv [Preprint] 2020. 10.1101/2020.04.05.20047944. PMC7436218 32790891

[88] Guiot J, Vaidyanathan A, Deprez L, et al. Development and validation of an automated radiomic CT signature for detecting covid-19. medRxiv [Preprint] 2020. 10.1101/2020.04.28.20082966. PMC7823620 33396587

[89] Guo Y, Liu Y, Lu J, et al. Development and validation of an early warning score (EWAS) for predicting clinical deterioration in patients with coronavirus disease 2019. medRxiv [Preprint] 2020. 10.1101/2020.04.17.20064691

[90] Hu C Liu Z Jiang Y . Early prediction of mortality risk among patients with severe COVID-19, using machine learning. Int J Epidemiol 2021;49:1918-29. 10.1093/ije/dyaa171. 32997743 PMC7543461

[91] Hu H Yao N Qiu Y . Comparing rapid scoring systems in mortality prediction of critical ill patients with novel coronavirus disease. Acad Emerg Med 2020;27:461-8. 10.1111/acem.13992 32311790 PMC7264631

[92] Hu R Ruan G Xiang S . Automated diagnosis of covid-19 using deep learning and data augmentation on chest CT. medRxiv [Preprint] 2020. 10.1101/2020.04.24.20078998.

[93] Islam MT, Fleischer JW. Distinguishing L and H phenotypes of covid-19 using a single x-ray image. medRxiv [Preprint] 2020. 10.1101/2020.04.27.20081984.

[94] Ji D Zhang D Xu J . Prediction for progression risk in patients with covid-19 pneumonia: the CALL score. Clin Infect Dis 2020;ciaa414. 10.1093/cid/ciaa414 32271369 PMC7184473

[95] Jiang X Coffee M Bari A . Towards an artificial intelligence framework for data-driven prediction of coronavirus clinical severity. Computers. Materials & Continua 2020;63:537-51 10.32604/cmc.2020.010691.

[96] Jiang Z, Hu M, Fan L, et al. Combining visible light and infrared imaging for efficient detection of respiratory infections such as covid-19 on portable device. ArXiv e-prints [Preprint] 2020.

[97] Kana GEB, Kana ZMG, Kana DAF, et al. A web-based diagnostic tool for covid-19 using machine learning on chest radiographs (CXR). medRxiv [Preprint] 2020. 10.1101/2020.04.21.20063263.

[98] Rezaul KM, Döhmen T, Rebholz-Schuhmann D, et al. DeepcovidExplainer: explainable covid-19 predictions based on chest x-ray images. ArXiv e-prints [Preprint] 2020.

[99] Khan AI Shah JL Bhat MM . CoroNet: A deep neural network for detection and diagnosis of covid-19 from chest x-ray images. Comput Methods Programs Biomed 2020;196:105581. 10.1016/j.cmpb.2020.105581 32534344 PMC7274128

[100] Kumar R, Arora R, Bansal V, et al. Accurate prediction of covid-19 using chest x-ray images through deep feature learning model with SMOTE and machine learning classifiers. medRxiv [Preprint] 2020. 10.1101/2020.04.13.20063461.

[101] Kurstjens S, van der Horst A, Herpers R, et al. Rapid identification of SARS-CoV-2-infected patients at the emergency department using routine testing. medRxiv [Preprint] 2020. 10.1101/2020.04.20.20067512 32598302

[102] Levy TJ, Richardson S, Coppa K, et al. Estimating survival of hospitalized covid-19 patients from admission information. medRxiv [Preprint] 2020. 10.1101/2020.04.22.20075416.

[103] Li Z, Zhong Z, Li Y, et al. From community acquired pneumonia to covid-19: a deep learning based method for quantitative analysis of covid-19 on thick-section CT scans. medRxiv [Preprint] 2020. 10.1101/2020.04.17.20070219. PMC7368602 32683550

[104] Liu Q Fang X Tokuno S . A web visualization tool using T cell subsets as the predictor to evaluate COVID-19 patient’s severity. PLoS One 2020;15:e0239695. 10.1371/journal.pone.0239695. 32970753 PMC7514096

[105] Lyu P Liu X Zhang R Shi L Gao J . The performance of chest CT in evaluating the clinical severity of covid-19 pneumonia: identifying critical cases based on CT characteristics. Invest Radiol 2020;55:412-21. 10.1097/RLI.0000000000000689 32304402 PMC7173027

[106] McRae MP Simmons GW Christodoulides NJ . Clinical decision support tool and rapid point-of-care platform for determining disease severity in patients with covid-19. Lab Chip 2020;20:2075-85. 10.1039/D0LC00373E 32490853 PMC7360344

[107] Mei X Lee HC Diao KY . Artificial intelligence-enabled rapid diagnosis of patients with covid-19. Nat Med 2020;26:1224-8. 10.1038/s41591-020-0931-3 32427924 PMC7446729

[108] Menni C Valdes AM Freidin MB . Real-time tracking of self-reported symptoms to predict potential covid-19. Nat Med 2020;26:1037-40. 10.1038/s41591-020-0916-2 32393804 PMC7751267

[109] Moutounet-Cartan PGB. Deep convolutional neural networks to diagnose covid-19 and other pneumonia diseases from posteroanterior chest x-rays. ArXiv e-prints [Preprint] 2020

[110] Ozturk T Talo M Yildirim EA Baloglu UB Yildirim O Rajendra Acharya U . Automated detection of covid-19 cases using deep neural networks with X-ray images. Comput Biol Med 2020;121:103792. 10.1016/j.compbiomed.2020.103792 32568675 PMC7187882

[111] Rahimzadeh M Attar A . A modified deep convolutional neural network for detecting covid-19 and pneumonia from chest X-ray images based on the concatenation of Xception and ResNet50V2. Inform Med Unlocked 2020;19:100360. 10.1016/j.imu.2020.100360 32501424 PMC7255267

[112] Rehman A, Naz S, Khan A, et al. Improving coronavirus (covid-19) diagnosis using deep transfer learning. medRxiv [Preprint] 2020. 10.1101/2020.04.11.20054643.

[113] Singh D Kumar V Vaishali NA Kaur M . Classification of covid-19 patients from chest CT images using multi-objective differential evolution-based convolutional neural networks. Eur J Clin Microbiol Infect Dis 2020;39:1379-89. 10.1007/s10096-020-03901-z 32337662 PMC7183816

[114] Singh K Valley TS Tang S . Evaluating a Widely Implemented Proprietary Deterioration Index Model among Hospitalized Patients with COVID-19. Ann Am Thorac Soc 2021;18:1129-37. 10.1513/AnnalsATS.202006-698OC. 33357088 PMC8328366

[115] Soares F, Villavicencio A, Anzanello MJ, et al. A novel high specificity covid-19 screening method based on simple blood exams and artificial intelligence. medRxiv [Preprint] 2020. 10.1101/2020.04.10.20061036.

[116] Tordjman M, Mekki A, Mali RD, et al. Pre-test probability for SARS-Cov-2-related Infection Score: the PARIS score. medRxiv [Preprint] 2020. 10.1101/2020.04.28.20081687. PMC7745977 33332360

[117] Ucar F Korkmaz D . covidiagnosis-Net: Deep Bayes-SqueezeNet based diagnosis of the coronavirus disease 2019 (covid-19) from X-ray images. Med Hypotheses 2020;140:109761. 10.1016/j.mehy.2020.109761 32344309 PMC7179515

[118] Vaid A Somani S Russak AJ . Machine Learning to Predict Mortality and Critical Events in a Cohort of Patients With COVID-19 in New York City: Model Development and Validation. J Med Internet Res 2020;22:e24018. 10.2196/24018. 33027032 PMC7652593

[119] Vazquez Guillamet C, Vazquez Guillamet R, Kramer AA, et al. Toward a covid-19 score-risk assessments and registry. medRxiv [Preprint] 2020. 10.1101/2020.04.15.20066860.

[120] Wang c, Deng R, Gou L, et al. Preliminary study to identify severe from moderate cases of covid-19 using NLR&RDW-SD combination parameter. medRxiv [Preprint] 2020. 10.1101/2020.04.09.20058594. PMC7290538 32566620

[121] Wu Y-H, Gao S-H, Mei J, et al. JCS: an explainable covid-19 diagnosis system by joint classification and segmentation. ArXiv e-prints [Preprint] 2020. 10.1109/TIP.2021.3058783 33600316

[122] Zhang H, Shi T, Wu X, et al. Risk prediction for poor outcome and death in hospital in-patients with covid-19: derivation in Wuhan, China and external validation in London. medRxiv [Preprint] 2020. 10.1101/2020.04.28.20082222.

[123] Zhao B, Wei Y, Sun W, et al. Distinguish coronavirus disease 2019 patients in general surgery emergency by CIAAD scale: development and validation of a prediction model based on 822 cases in China. medRxiv [Preprint] 2020. 10.1101/2020.04.18.20071019. PMC8119634 33996842

[124] Zhu Z Cai T Fan L . Clinical value of immune-inflammatory parameters to assess the severity of coronavirus disease 2019. Int J Infect Dis 2020;95:332-9. 10.1016/j.ijid.2020.04.041 32334118 PMC7195003

[125] Gong J Ou J Qiu X . A tool to early predict severe corona virus disease 2019 (covid-19) : a multicenter study using the risk nomogram in Wuhan and Guangdong, China. Clin Infect Dis 2020;ciaa443. 32296824 10.1093/cid/ciaa443 PMC7184338

[126] Apostolopoulos ID Aznaouridis SI Tzani MA . Extracting possibly representative covid-19 biomarkers from x-ray images with deep learning approach and image data related to pulmonary diseases. J Med Biol Eng 2020;40:462-9. 10.1007/s40846-020-00529-4. 32412551 PMC7221329

[127] Ardakani AA Kanafi AR Acharya UR Khadem N Mohammadi A . Application of deep learning technique to manage covid-19 in routine clinical practice using CT images: results of 10 convolutional neural networks. Comput Biol Med 2020;121:103795. 10.1016/j.compbiomed.2020.103795 32568676 PMC7190523

[128] Bar S Lecourtois A Diouf M . The association of lung ultrasound images with covid-19 infection in an emergency room cohort. Anaesthesia 2020;75:1620-5. 10.1111/anae.15175 32520406 PMC7300460

[129] Bi X Su Z Yan H . Prediction of severe illness due to covid-19 based on an analysis of initial fibrinogen to albumin ratio and platelet count. Platelets 2020;31:674-9. 10.1080/09537104.2020.1760230 32367765 PMC7212543

[130] Borghesi A Zigliani A Golemi S . Chest x-ray severity index as a predictor of in-hospital mortality in coronavirus disease 2019: a study of 302 patients from Italy. Int J Infect Dis 2020;96:291-3. 10.1016/j.ijid.2020.05.021 32437939 PMC7207134

[131] Burian E Jungmann F Kaissis GA . Intensive care risk estimation in covid-19 pneumonia based on clinical and imaging parameters: experiences from the Munich cohort. J Clin Med 2020;9:E1514. 10.3390/jcm9051514 32443442 PMC7291055

[132] Cecconi M Piovani D Brunetta E . Early predictors of clinical deterioration in a cohort of 239 patients hospitalized for covid-19 infection in Lombardy, Italy. J Clin Med 2020;9:E1548. 10.3390/jcm9051548 32443899 PMC7290833

[133] Cheng FY Joshi H Tandon P . Using machine learning to predict ICU transfer in hospitalized covid-19 patients. J Clin Med 2020;9:E1668. 10.3390/jcm9061668 32492874 PMC7356638

[134] Choi MH Ahn H Ryu HS . Clinical characteristics and disease progression in early-stage covid-19 patients in South Korea. J Clin Med 2020;9:E1959. 10.3390/jcm9061959 32585855 PMC7355553

[135] Clemency BM Varughese R Scheafer DK . Symptom criteria for covid-19 testing of heath care workers. Acad Emerg Med 2020;27:469-74. 10.1111/acem.14009 32396670 PMC7272901

[136] Dong Y Zhou H Li M . A novel simple scoring model for predicting severity of patients with SARS-CoV-2 infection. Transbound Emerg Dis 2020;67:2823-9. 10.1111/tbed.13651 32469137 PMC7283685

[137] El Asnaoui K Chawki Y . Using X-ray images and deep learning for automated detection of coronavirus disease. J Biomol Struct Dyn 2020;1-12. 32397844 10.1080/07391102.2020.1767212 PMC7256347

[138] Fu L Li Y Cheng A Pang P Shu Z . A novel machine learning-derived radiomic signature of the whole lung differentiates stable from progressive covid-19 infection: a retrospective cohort study. J Thorac Imaging 2020. 10.1097/RTI.0000000000000544 32555006 PMC7682797

[139] Galloway JB Norton S Barker RD . A clinical risk score to identify patients with covid-19 at high risk of critical care admission or death: An observational cohort study. J Infect 2020;81:282-8. 10.1016/j.jinf.2020.05.064 32479771 PMC7258846

[140] Gezer NS Ergan B Barış MM . covid-19 S: A new proposal for diagnosis and structured reporting of covid-19 on computed tomography imaging. Diagn Interv Radiol 2020;26:315-22. 10.5152/dir.2020.20351 32558646 PMC7360076

[141] Gidari A De Socio GV Sabbatini S Francisci D . Predictive value of National Early Warning Score 2 (NEWS2) for intensive care unit admission in patients with SARS-CoV-2 infection. Infect Dis (Lond) 2020;52:698-704. 10.1080/23744235.2020.1784457 32584161

[142] Hong Y Wu X Qu J Gao Y Chen H Zhang Z . Clinical characteristics of Coronavirus Disease 2019 and development of a prediction model for prolonged hospital length of stay. Ann Transl Med 2020;8:443. 10.21037/atm.2020.03.147 32395487 PMC7210129

[143] Huang D Wang T Chen Z Yang H Yao R Liang Z . A novel risk score to predict diagnosis with coronavirus disease 2019 (covid-19) in suspected patients: a retrospective, multicenter, and observational study. J Med Virol 2020;92:2709-17. 10.1002/jmv.26143 32510164 PMC7300577

[144] Huang J Cheng A Lin S Zhu Y Chen G . Individualized prediction nomograms for disease progression in mild covid-19. J Med Virol 2020;92:2074-80. 10.1002/jmv.25969 32369205 PMC7267495

[145] Jehi L Ji X Milinovich A . Individualizing risk prediction for positive coronavirus disease 2019 testing: results from 11,672 patients. Chest 2020;158:1364-75. 10.1016/j.chest.2020.05.580 32533957 PMC7286244

[146] Joshi RP Pejaver V Hammarlund NE . A predictive tool for identification of SARS-CoV-2 PCR-negative emergency department patients using routine test results. J Clin Virol 2020;129:104502. 10.1016/j.jcv.2020.104502 32544861 PMC7286235

[147] Knight SR Ho A Pius R ISARIC4C investigators . Risk stratification of patients admitted to hospital with covid-19 using the ISARIC WHO Clinical Characterisation Protocol: development and validation of the 4C Mortality Score. BMJ 2020;370:m3339. 10.1136/bmj.m3339 32907855 PMC7116472

[148] Ko H Chung H Kang WS . covid-19 pneumonia diagnosis using a simple 2D deep learning framework with a single chest CT image: model development and validation. J Med Internet Res 2020;22:e19569. 10.2196/19569 32568730 PMC7332254

[149] Li Q Zhang J Ling Y . A simple algorithm helps early identification of SARS-CoV-2 infection patients with severe progression tendency. Infection 2020;48:577-84. 10.1007/s15010-020-01446-z 32440918 PMC7240242

[150] Li Y Yang Z Ai T Wu S Xia L . Association of “initial CT” findings with mortality in older patients with coronavirus disease 2019 (covid-19). Eur Radiol 2020;30:6186-93. 10.1007/s00330-020-06969-5 32524220 PMC7283986

[151] Li Z Zeng B Lei P . Differentiating pneumonia with and without covid-19 using chest CT images: from qualitative to quantitative. J Xray Sci Technol 2020;28:583-9. 10.3233/XST-200689 32568167 PMC7505000

[152] Liang W Liang H Ou L China Medical Treatment Expert Group for covid-19 . Development and Validation of a Clinical Risk Score to Predict the Occurrence of Critical Illness in Hospitalized Patients With covid-19. JAMA Intern Med 2020;180:1081-9. 10.1001/jamainternmed.2020.2033 32396163 PMC7218676

[153] Liu F Zhang Q Huang C . CT quantification of pneumonia lesions in early days predicts progression to severe illness in a cohort of covid-19 patients. Theranostics 2020;10:5613-22. 10.7150/thno.45985 32373235 PMC7196293

[154] Liu X Shi S Xiao J . Prediction of the severity of coronavirus disease 2019 and its adverse clinical outcomes. Jpn J Infect Dis 2020;73:404-10. 10.7883/yoken.JJID.2020.194 32475880

[155] Liu Y Wang Z Ren J . A covid-19 risk assessment decision support system for general practitioners: design and development study. J Med Internet Res 2020;22:e19786. 10.2196/19786 32540845 PMC7332157

[156] Liu YP Li GM He J . Combined use of the neutrophil-to-lymphocyte ratio and CRP to predict 7-day disease severity in 84 hospitalized patients with covid-19 pneumonia: a retrospective cohort study. Ann Transl Med 2020;8:635. 10.21037/atm-20-2372 32566572 PMC7290615

[157] Lorente-Ros A Monteagudo Ruiz JM Rincón LM . Myocardial injury determination improves risk stratification and predicts mortality in covid-19 patients. Cardiol J 2020;27:489-96. 10.5603/CJ.a2020.0089 32589258 PMC8078990

[158] Luo L Luo Z Jia Y . CT differential diagnosis of covid-19 and non-covid-19 in symptomatic suspects: a practical scoring method. BMC Pulm Med 2020;20:129. 10.1186/s12890-020-1170-6 32381057 PMC7203713

[159] Luo M Liu J Jiang W Yue S Liu H Wei S . IL-6 and CD8+ T cell counts combined are an early predictor of in-hospital mortality of patients with covid-19. JCI Insight 2020;5:139024. 10.1172/jci.insight.139024 32544099 PMC7406244

[160] Luo Y Yuan X Xue Y . Using a diagnostic model based on routine laboratory tests to distinguish patients infected with SARS-CoV-2 from those infected with influenza virus. Int J Infect Dis 2020;95:436-40. 10.1016/j.ijid.2020.04.078 32371192 PMC7194039

[161] Matos J Paparo F Mussetto I . Evaluation of novel coronavirus disease (covid-19) using quantitative lung CT and clinical data: prediction of short-term outcome. Eur Radiol Exp 2020;4:39. 10.1186/s41747-020-00167-0 32592118 PMC7318726

[162] Mazzaccaro D Giacomazzi F Giannetta M . Non-overt coagulopathy in non-ICU patients with mild to moderate covid-19 pneumonia. J Clin Med 2020;9:E1781. 10.3390/jcm9061781 32521707 PMC7355651

[163] Murphy K Smits H Knoops AJG . Covid-19 on the chest radiograph: a multireader evaluation of an artificial intelligence system. Radiology 2020;296:E166-72. 10.1148/radiol.2020201874 32384019 PMC7437494

[164] Obeid JS Davis M Turner M Obeid JS Davis M Turner M . An artificial intelligence approach to covid-19 infection risk assessment in virtual visits: A case report. J Am Med Inform Assoc 2020;27:1321-5. 10.1093/jamia/ocaa105 32449766 PMC7313981

[165] Pu J Leader J Bandos A . Any unique image biomarkers associated with covid-19? Eur Radiol 2020;30:6221-7. 10.1007/s00330-020-06956-w 32462445 PMC7253230

[166] Rajaraman S Antani S . Weakly labeled data augmentation for deep learning: a study on covid-19 detection in chest x-rays. Diagnostics (Basel) 2020;10:E358. 10.3390/diagnostics10060358 32486140 PMC7345787

[167] Roland LT Gurrola JG 2nd Loftus PA Cheung SW Chang JL . Smell and taste symptom-based predictive model for covid-19 diagnosis. Int Forum Allergy Rhinol 2020;10:832-8. 10.1002/alr.22602 32363809 PMC7267242

[168] Satici C Demirkol MA Sargin Altunok E . Performance of pneumonia severity index and CURB-65 in predicting 30-day mortality in patients with covid-19. Int J Infect Dis 2020;98:84-9. 10.1016/j.ijid.2020.06.038 32553714 PMC7293841

[169] Song J Wang H Liu Y . End-to-end automatic differentiation of the coronavirus disease 2019 (covid-19) from viral pneumonia based on chest CT. Eur J Nucl Med Mol Imaging 2020;47:2516-24. 10.1007/s00259-020-04929-1 32567006 PMC7306401

[170] Sun L Song F Shi N . Combination of four clinical indicators predicts the severe/critical symptom of patients infected covid-19. J Clin Virol 2020;128:104431. 10.1016/j.jcv.2020.104431 32442756 PMC7219384

[171] Toraih EA Elshazli RM Hussein MH . Association of cardiac biomarkers and comorbidities with increased mortality, severity, and cardiac injury in covid-19 patients: A meta-regression and decision tree analysis. J Med Virol 2020;92:2473-88. 10.1002/jmv.26166 32530509 PMC7307124

[172] Tuncer T Dogan S Ozyurt F . An automated residual exemplar local binary pattern and iterative relieff based covid-19 detection method using chest x-ray image. Chemometr Intell Lab Syst 2020;203:104054. 10.1016/j.chemolab.2020.104054 32427226 PMC7233238

[173] Vaid S Kalantar R Bhandari M . Deep learning covid-19 detection bias: accuracy through artificial intelligence. Int Orthop 2020;44:1539-42. 10.1007/s00264-020-04609-7 32462314 PMC7251557

[174] Vultaggio A Vivarelli E Virgili G . Prompt predicting of early clinical deterioration of moderate-to-severe covid-19 patients: usefulness of a combined score using IL-6 in a preliminary study. J Allergy Clin Immunol Pract 2020;8:2575-2581.e2. 10.1016/j.jaip.2020.06.013 32565226 PMC7303032

[175] Wang F Hou H Wang T . Establishing a model for predicting the outcome of covid-19 based on combination of laboratory tests. Travel Med Infect Dis 2020;36:101782. 10.1016/j.tmaid.2020.101782 32526372 PMC7836898

[176] Wang K Zuo P Liu Y . Clinical and laboratory predictors of in-hospital mortality in patients with coronavirus disease-2019: a cohort study in Wuhan, China. Clin Infect Dis 2020;71:2079-88. 10.1093/cid/ciaa538 32361723 PMC7197616

[177] Wang L Liu Y Zhang T . Differentiating between 2019 novel coronavirus pneumonia and influenza using a nonspecific laboratory marker-based dynamic nomogram. Open Forum Infect Dis 2020;7:a169. 10.1093/ofid/ofaa169 32490031 PMC7239104

[178] Wu S Du Z Shen S . Identification and validation of a novel clinical signature to predict the prognosis in confirmed covid-19 patients. Clin Infect Dis 2020;ciaa793. 32556293 10.1093/cid/ciaa793 PMC7337707

[179] Wu X Hui H Niu M . Deep learning-based multi-view fusion model for screening 2019 novel coronavirus pneumonia: a multicentre study. Eur J Radiol 2020;128:109041. 10.1016/j.ejrad.2020.109041 32408222 PMC7198437

[180] Yang P Wang P Song Y Zhang A Yuan G Cui Y . A retrospective study on the epidemiological characteristics and establishment of an early warning system of severe covid-19 patients. J Med Virol 2020;92:2173-80. 10.1002/jmv.26022 32410285 PMC7272979

[181] Yang Y Shen C Li J . Plasma IP-10 and MCP-3 levels are highly associated with disease severity and predict the progression of covid-19. J Allergy Clin Immunol 2020;146:119-127.e4. 10.1016/j.jaci.2020.04.027 32360286 PMC7189843

[182] Yu C Lei Q Li W . Clinical characteristics, associated factors, and predicting covid-19 mortality risk: a retrospective study in Wuhan, China. Am J Prev Med 2020;59:168-75. 10.1016/j.amepre.2020.05.002 32564974 PMC7250782

[183] Zhang C Qin L Li K . A novel scoring system for prediction of disease severity in covid-19. Front Cell Infect Microbiol 2020;10:318. 10.3389/fcimb.2020.00318 32582575 PMC7292148

[184] Zhang K Liu X Shen J . Clinically applicable AI System for accurate diagnosis, quantitative measurements, and prognosis of covid-19 pneumonia using computed tomography. Cell 2020;181:1423-1433.e11. 10.1016/j.cell.2020.04.045 32416069 PMC7196900

[185] Zheng QN Xu MY Zheng YL Wang XY Zhao H . Prediction of the rehabilitation duration and risk management for mild-moderate covid-19. Disaster Med Public Health Prep 2020;14:652-7. 10.1017/dmp.2020.214 32576328 PMC7369334

[186] Zhou Y He Y Yang H . Development and validation a nomogram for predicting the risk of severe covid-19: a multi-center study in Sichuan, China. PLoS One 2020;15:e0233328. 10.1371/journal.pone.0233328 32421703 PMC7233581

[187] Zou X Li S Fang M . Acute physiology and chronic health evaluation II score as a predictor of hospital mortality in patients of coronavirus disease 2019. Crit Care Med 2020;48:e657-65. 10.1097/CCM.0000000000004411 32697506 PMC7217128

[188] Hu H Du H Li J . Early prediction and identification for severe patients during the pandemic of COVID-19: A severe COVID-19 risk model constructed by multivariate logistic regression analysis. J Glob Health 2020;10:020510. 10.7189/jogh.10.020510. 33110593 PMC7567445

[189] Abdulaal A Patel A Charani E . Comparison of deep learning with regression analysis in creating predictive models for SARS-CoV-2 outcomes. BMC Med Inform Decis Mak 2020;20:299. 10.1186/s12911-020-01316-6. 33213435 PMC7676403

[190] Abdulaal A Patel A Charani E Denny S Mughal N Moore L . Prognostic Modeling of COVID-19 Using Artificial Intelligence in the United Kingdom: Model Development and Validation. J Med Internet Res 2020;22:e20259. 10.2196/20259. 32735549 PMC7451108

[191] Acar HC Can G Karaali R . An easy-to-use nomogram for predicting in-hospital mortality risk in COVID-19: a retrospective cohort study in a university hospital. BMC Infect Dis 2021;21:148. 10.1186/s12879-021-05845-x. 33546639 PMC7862983

[192] Al Hassan H Cocks E Jesani L Lewis S Szakmany T . Clinical Risk Prediction Scores in Coronavirus Disease 2019: Beware of Low Validity and Clinical Utility. Crit Care Explor 2020;2:e0253. 10.1097/CCE.0000000000000253. 33134944 PMC7581153

[193] Alafif T Alotaibi R Albassam A Almudhayyani A . On the prediction of isolation, release, and decease states for COVID-19 patients: A case study in South Korea. ISA Trans 2022;124:191-6. 10.1016/j.isatra.2020.12.053. 33451801 PMC7785285

[194] Aliberti MJR Covinsky KE Garcez FB . A fuller picture of COVID-19 prognosis: the added value of vulnerability measures to predict mortality in hospitalised older adults. Age Ageing 2021;50:32-9. 10.1093/ageing/afaa240. 33068099 PMC7665299

[195] Allenbach Y Saadoun D Maalouf G DIMICOVID . Development of a multivariate prediction model of intensive care unit transfer or death: A French prospective cohort study of hospitalized COVID-19 patients. PLoS One 2020;15:e0240711. 10.1371/journal.pone.0240711. 33075088 PMC7571674

[196] Altschul DJ Unda SR Benton J . A novel severity score to predict inpatient mortality in COVID-19 patients. Sci Rep 2020;10:16726. 10.1038/s41598-020-73962-9. 33028914 PMC7542454

[197] Álvarez-Mon M Ortega MA Gasulla Ó . A Predictive Model and Risk Factors for Case Fatality of COVID-19. J Pers Med 2021;11:36. 10.3390/jpm11010036. 33430129 PMC7827846

[198] An C Lim H Kim DW Chang JH Choi YJ Kim SW . Machine learning prediction for mortality of patients diagnosed with COVID-19: a nationwide Korean cohort study. Sci Rep 2020;10:18716. 10.1038/s41598-020-75767-2. 33127965 PMC7599238

[199] Anurag A Preetam M . Validation of PSI/PORT, CURB-65 and SCAP scoring system in COVID-19 pneumonia for prediction of disease severity and 14-day mortality. Clin Respir J 2021;15:467-71. 10.1111/crj.13326. 33417280

[200] Artero A Madrazo M Fernández-Garcés M SEMI-COVID-19 Network . Severity Scores in COVID-19 Pneumonia: a Multicenter, Retrospective, Cohort Study. J Gen Intern Med 2021;36:1338-45. 10.1007/s11606-021-06626-7. 33575909 PMC7878165

[201] Arvind V Kim JS Cho BH Geng E Cho SK . Development of a machine learning algorithm to predict intubation among hospitalized patients with COVID-19. J Crit Care 2021;62:25-30. 10.1016/j.jcrc.2020.10.033. 33238219 PMC7669246

[202] Assaf D Gutman Y Neuman Y . Utilization of machine-learning models to accurately predict the risk for critical COVID-19. Intern Emerg Med 2020;15:1435-43. 10.1007/s11739-020-02475-0. 32812204 PMC7433773

[203] Baker KF Hanrath AT Schim van der Loeff I Kay LJ Back J Duncan CJ . National Early Warning Score 2 (NEWS2) to identify inpatient COVID-19 deterioration: a retrospective analysis. Clin Med (Lond) 2021;21:84-9. 10.7861/clinmed.2020-0688. 33547065 PMC8002770

[204] Bartoletti M Giannella M Scudeller L PREDICO study group . Development and validation of a prediction model for severe respiratory failure in hospitalized patients with SARS-CoV-2 infection: a multicentre cohort study (PREDI-CO study). Clin Microbiol Infect 2020;26:1545-53. 10.1016/j.cmi.2020.08.003. 32781244 PMC7414420

[205] Bello-Chavolla OY Antonio-Villa NE Ortiz-Brizuela E . Validation and repurposing of the MSL-COVID-19 score for prediction of severe COVID-19 using simple clinical predictors in a triage setting: The Nutri-CoV score. PLoS One 2020;15:e0244051. 10.1371/journal.pone.0244051. 33326502 PMC7743927

[206] Bellos I Lourida P Argyraki A . Development of a novel risk score for the prediction of critical illness amongst COVID-19 patients. Int J Clin Pract 2021;75:e13915. 10.1111/ijcp.13915. 33969593 PMC7883033

[207] Bennouar S Bachir Cherif A Kessira A Bennouar DE Abdi S . Development and validation of a laboratory risk score for the early prediction of COVID-19 severity and in-hospital mortality. Intensive Crit Care Nurs 2021;64:103012. 10.1016/j.iccn.2021.103012. 33487518 PMC7834685

[208] Bernabeu-Wittel M Ternero-Vega JE Díaz-Jiménez P . Death risk stratification in elderly patients with covid-19. A comparative cohort study in nursing homes outbreaks. Arch Gerontol Geriatr 2020;91:104240. 10.1016/j.archger.2020.104240. 32877792 PMC7446617

[209] Bertsimas D Lukin G Mingardi L Hellenic COVID-19 Study Group . COVID-19 mortality risk assessment: An international multi-center study. PLoS One 2020;15:e0243262. 10.1371/journal.pone.0243262. 33296405 PMC7725386

[210] Berzuini C Hannan C King A . Value of dynamic clinical and biomarker data for mortality risk prediction in COVID-19: a multicentre retrospective cohort study. BMJ Open 2020;10:e041983. 10.1136/bmjopen-2020-041983. 32967887 PMC7513423

[211] Boero E Rovida S Schreiber A . The COVID-19 Worsening Score (COWS)-a predictive bedside tool for critical illness. Echocardiography 2021;38:207-16. 10.1111/echo.14962. 33491261 PMC8013873

[212] Bolourani S Brenner M Wang P Northwell COVID-19 Research Consortium . A Machine Learning Prediction Model of Respiratory Failure Within 48 Hours of Patient Admission for COVID-19: Model Development and Validation. J Med Internet Res 2021;23:e24246. 10.2196/24246. 33476281 PMC7879728

[213] Booth AL Abels E McCaffrey P . Development of a prognostic model for mortality in COVID-19 infection using machine learning. Mod Pathol 2021;34:522-31. 10.1038/s41379-020-00700-x. 33067522 PMC7567420

[214] Bradley P Frost F Tharmaratnam K Wootton DG NW Collaborative Organisation for Respiratory Research . Utility of established prognostic scores in COVID-19 hospital admissions: multicentre prospective evaluation of CURB-65, NEWS2 and qSOFA. BMJ Open Respir Res 2020;7:e000729. 10.1136/bmjresp-2020-000729. 33293361 PMC7722817

[215] Burdick H Lam C Mataraso S . Prediction of respiratory decompensation in Covid-19 patients using machine learning: The READY trial. Comput Biol Med 2020;124:103949. 10.1016/j.compbiomed.2020.103949. 32798922 PMC7410013

[216] Burke H Freeman A Cellura DC REACT COVID investigators . Inflammatory phenotyping predicts clinical outcome in COVID-19. Respir Res 2020;21:245. 10.1186/s12931-020-01511-z. 32962703 PMC7506817

[217] Cao G Li P Chen Y . A Risk Prediction Model for Evaluating the Disease Progression of COVID-19 Pneumonia. Front Med (Lausanne) 2020;7:556886. 10.3389/fmed.2020.556886. 33251226 PMC7675774

[218] Cao L Zhang S Wang E . The CB index predicts prognosis of critically ill COVID-19 patients. Ann Transl Med 2020;8:1654. 10.21037/atm-20-7447. 33490166 PMC7812180

[219] Caro-Codón J Lip GYH Rey JR . Prediction of thromboembolic events and mortality by the CHADS2 and the CHA2DS2-VASc in COVID-19. Europace 2021;23:937-47. 10.1093/europace/euab015. 33564822 PMC7928912

[220] Cetinkal G Kocas BB Ser OS . Assessment of the Modified CHA2DS2VASc Risk Score in Predicting Mortality in Patients Hospitalized With COVID-19. Am J Cardiol 2020;135:143-9. 10.1016/j.amjcard.2020.08.040. 32861734 PMC7453224

[221] Chao H Fang X Zhang J . Integrative analysis for COVID-19 patient outcome prediction. Med Image Anal 2021;67:101844. 10.1016/j.media.2020.101844. 33091743 PMC7553063

[222] Chen H Chen R Yang H . Development and validation of a nomogram using on admission routine laboratory parameters to predict in-hospital survival of patients with COVID-19. J Med Virol 2021;93:2332-9. 10.1002/jmv.26713. 33289142

[223] Chen H Zeng M Wang X . A CT-based radiomics nomogram for predicting prognosis of coronavirus disease 2019 (COVID-19) radiomics nomogram predicting COVID-19. Br J Radiol 2021;94:20200634. 10.1259/bjr.20200634. 33296222 PMC7774709

[224] Chen X Peng F Zhou X . Predicting severe or critical symptoms in hospitalized patients with COVID-19 from Yichang, China. Aging (Albany NY) 2020;13:1608-19. 10.18632/aging.202261. 33318316 PMC7880337

[225] Chen Y Linli Z Lei Y . Risk factors for mortality in critically ill patients with COVID-19 in Huanggang, China: A single-center multivariate pattern analysis. J Med Virol 2021;93:2046-55. 10.1002/jmv.26572. 32997344 PMC7537509

[226] Cheng A Hu L Wang Y . Diagnostic performance of initial blood urea nitrogen combined with D-dimer levels for predicting in-hospital mortality in COVID-19 patients. Int J Antimicrob Agents 2020;56:106110. 10.1016/j.ijantimicag.2020.106110. 32712332 PMC7377803

[227] Cheng P Wu H Yang J . Pneumonia scoring systems for severe COVID-19: which one is better. Virol J 2021;18:33. 10.1186/s12985-021-01502-6. 33568204 PMC7874994

[228] Cho SY Park SS Song MK Bae YY Lee DG Kim DW . Prognosis Score System to Predict Survival for COVID-19 Cases: a Korean Nationwide Cohort Study. J Med Internet Res 2021;23:e26257. 10.2196/26257. 33539312 PMC7901599

[229] Chow DS Glavis-Bloom J Soun JE . Development and external validation of a prognostic tool for COVID-19 critical disease. PLoS One 2020;15:e0242953. 10.1371/journal.pone.0242953. 33296357 PMC7725393

[230] Çınar T Hayıroğlu Mİ Çiçek V . Is prognostic nutritional index a predictive marker for estimating all-cause in-hospital mortality in COVID-19 patients with cardiovascular risk factors? Heart Lung 2021;50:307-12. 10.1016/j.hrtlng.2021.01.006. 33482433 PMC7832700

[231] Clift AK Coupland CAC Keogh RH . Living risk prediction algorithm (QCOVID) for risk of hospital admission and mortality from coronavirus 19 in adults: national derivation and validation cohort study. BMJ 2020;371:m3731. 10.1136/bmj.m3731. 33082154 PMC7574532

[232] Covino M De Matteis G Burzo ML GEMELLI AGAINST COVID-19 Group . Predicting in-hospital mortality in covid-19 older patients with specifically developed scores. J Am Geriatr Soc 2021;69:37-43. 10.1111/jgs.16956. 33197278 PMC7753731

[233] Covino M Sandroni C Santoro M . Predicting intensive care unit admission and death for COVID-19 patients in the emergency department using early warning scores. Resuscitation 2020;156:84-91. 10.1016/j.resuscitation.2020.08.124. 32918985 PMC7480278

[234] Dai Z Zeng D Cui D . Prediction of COVID-19 Patients at High Risk of Progression to Severe Disease. Front Public Health 2020;8:574915. 10.3389/fpubh.2020.574915. 33330318 PMC7732480

[235] De Giorgi A Fabbian F Greco S OUTcome and COMorbidity Evaluation of INTernal MEDicine COVID19 (OUTCOME-INTMED-COV19) Study Collaborators . Prediction of in-hospital mortality of patients with SARS-CoV-2 infection by comorbidity indexes: an Italian internal medicine single center study. Eur Rev Med Pharmacol Sci 2020;24:10258-66. 10.26355/eurrev_202010_23250. 33090437

[236] Ding ZY Li GX Chen L Tongji Multidisciplinary Team for Treating COVID-19 (TTTC) . Association of liver abnormalities with in-hospital mortality in patients with COVID-19. J Hepatol 2021;74:1295-302. 10.1016/j.jhep.2020.12.012. 33347952 PMC7749734

[237] Doganci S Ince ME Ors N . A new COVID-19 prediction scoring model for in-hospital mortality: experiences from Turkey, single center retrospective cohort analysis. Eur Rev Med Pharmacol Sci 2020;24:10247-57. 10.26355/eurrev_202010_23249. 33090436

[238] Dong YM Sun J Li YX . Development and Validation of a Nomogram for Assessing Survival in Patients With COVID-19 Pneumonia. Clin Infect Dis 2021;72:652-60. 10.1093/cid/ciaa963. 32649738 PMC7454485

[239] Douville NJ Douville CB Mentz G . Clinically applicable approach for predicting mechanical ventilation in patients with COVID-19. Br J Anaesth 2021;126:578-89. 10.1016/j.bja.2020.11.034. 33454051 PMC7833820

[240] Dujardin RWG Hilderink BN Haksteen WE . Biomarkers for the prediction of venous thromboembolism in critically ill COVID-19 patients. Thromb Res 2020;196:308-12. 10.1016/j.thromres.2020.09.017. 32977128 PMC7491463

[241] Ebrahimian S Homayounieh F Rockenbach MABC . Artificial intelligence matches subjective severity assessment of pneumonia for prediction of patient outcome and need for mechanical ventilation: a cohort study. Sci Rep 2021;11:858. 10.1038/s41598-020-79470-0. 33441578 PMC7807029

[242] El-Solh AA Lawson Y Carter M El-Solh DA Mergenhagen KA . Comparison of in-hospital mortality risk prediction models from COVID-19. PLoS One 2020;15:e0244629. 10.1371/journal.pone.0244629. 33370409 PMC7769558

[243] España PP Bilbao A García-Gutiérrez S COVID-19-Osakidetza Working group . Predictors of mortality of COVID-19 in the general population and nursing homes. Intern Emerg Med 2021;16:1487-96. 10.1007/s11739-020-02594-8. 33400164 PMC7783294

[244] Esposito A Palmisano A Toselli M . Chest CT-derived pulmonary artery enlargement at the admission predicts overall survival in COVID-19 patients: insight from 1461 consecutive patients in Italy. Eur Radiol 2021;31:4031-41. 10.1007/s00330-020-07622-x. 33355697 PMC7755582

[245] Fan G Tu C Zhou F . Comparison of severity scores for COVID-19 patients with pneumonia: a retrospective study. Eur Respir J 2020;56:2002113. 10.1183/13993003.02113-2020. 32675205 PMC7366179

[246] Fan Q Zhu H Zhao J . Risk factors for myocardial injury in patients with coronavirus disease 2019 in China. ESC Heart Fail 2020. 10.1002/ehf2.13022. 33006440 PMC7537185

[247] Fan T Hao B Yang S . Nomogram for Predicting COVID-19 Disease Progression Based on Single-Center Data: Observational Study and Model Development. JMIR Med Inform 2020;8:e19588. 10.2196/19588. 32866109 PMC7485996

[248] Feng Z Yu Q Yao S . Early prediction of disease progression in COVID-19 pneumonia patients with chest CT and clinical characteristics. Nat Commun 2020;11:4968. 10.1038/s41467-020-18786-x. 33009413 PMC7532528

[249] Fernandes FT de Oliveira TA Teixeira CE Batista AFM Dalla Costa G Chiavegatto Filho ADP . A multipurpose machine learning approach to predict COVID-19 negative prognosis in São Paulo, Brazil. Sci Rep 2021;11:3343. 10.1038/s41598-021-82885-y. 33558602 PMC7870665

[250] Fernandez A Obiechina N Koh J Hong A Nandi A Reynolds TM . Survival prediction algorithms for COVID-19 patients admitted to a UK district general hospital. Int J Clin Pract 2021;75:e13974. 10.1111/ijcp.13974. 33368796 PMC7883072

[251] Ferrari D Milic J Tonelli R . Machine learning in predicting respiratory failure in patients with COVID-19 pneumonia-Challenges, strengths, and opportunities in a global health emergency. PLoS One 2020;15:e0239172. 10.1371/journal.pone.0239172. 33180787 PMC7660476

[252] Fisman DN Greer AL Hillmer M Tuite R . Derivation and Validation of Clinical Prediction Rules for COVID-19 Mortality in Ontario, Canada. Open Forum Infect Dis 2020;7:a463. 10.1093/ofid/ofaa463 33204755 PMC7650986

[253] Flores M Dayan I Roth H . Federated Learning used for predicting outcomes in SARS-COV-2 patients. Res Sq 2021. 10.21203/rs.3.rs-126892/v1

[254] Foieni F Sala G Mognarelli JG . Derivation and validation of the clinical prediction model for COVID-19. Intern Emerg Med 2020;15:1409-14. 10.1007/s11739-020-02480-3. 32930963 PMC7490315

[255] Fumagalli C Rozzini R Vannini M . Clinical risk score to predict in-hospital mortality in COVID-19 patients: a retrospective cohort study. BMJ Open 2020;10:e040729. 10.1136/bmjopen-2020-040729. 32978207 PMC7520809

[256] Gao Y Cai GY Fang W . Machine learning based early warning system enables accurate mortality risk prediction for COVID-19. Nat Commun 2020;11:5033. 10.1038/s41467-020-18684-2. 33024092 PMC7538910

[257] García CMM Herrero H . Assessment of risk scores in covid-19. Int J Clin Pract 2020;e13705. 10.1111/ijcp.13705. 32931634

[258] Garibaldi BT Fiksel J Muschelli J . Patient Trajectories Among Persons Hospitalized for COVID-19 : A Cohort Study. Ann Intern Med 2021;174:33-41. 10.7326/M20-3905. 32960645 PMC7530643

[259] Gavelli F Castello LM Bellan M . Clinical stability and in-hospital mortality prediction in COVID-19 patients presenting to the Emergency Department. Minerva Med 2021;112:118-23. 10.23736/S0026-4806.20.07074-3. 33104301

[260] Gerotziafas GT Sergentanis TN Voiriot G . Derivation and Validation of a Predictive Score for Disease Worsening in Patients with COVID-19. Thromb Haemost 2020;120:1680-90. 10.1055/s-0040-1716544. 32961572 PMC7869041

[261] Gonçalves LC Baggio S Weber M . COVID-19 Inmate Risk Appraisal (CIRA): development and validation of a screening tool to assess COVID-19 vulnerability in prisons. Swiss Med Wkly 2021;151:w20471. 10.4414/smw.2021.20471. 33580705

[262] Goodacre S Thomas B Sutton L . Derivation and validation of a clinical severity score for acutely ill adults with suspected COVID-19: The PRIEST observational cohort study. PLoS One 2021;16:e0245840. 10.1371/journal.pone.0245840. 33481930 PMC7822515

[263] Guan X Zhang B Fu M . Clinical and inflammatory features based machine learning model for fatal risk prediction of hospitalized COVID-19 patients: results from a retrospective cohort study. Ann Med 2021;53:257-66. 10.1080/07853890.2020.1868564. 33410720 PMC7799376

[264] Gude F Riveiro V Rodríguez-Núñez N . Development and validation of a clinical score to estimate progression to severe or critical state in COVID-19 pneumonia hospitalized patients. Sci Rep 2020;10:19794. 10.1038/s41598-020-75651-z. 33188225 PMC7666132

[265] Gude-Sampedro F Fernández-Merino C Ferreiro L . Development and validation of a prognostic model based on comorbidities to predict COVID-19 severity: a population-based study. Int J Epidemiol 2021;50:64-74. 10.1093/ije/dyaa209. 33349845 PMC7799114

[266] Gue YX Tennyson M Gao J Ren S Kanji R Gorog DA . Development of a novel risk score to predict mortality in patients admitted to hospital with COVID-19. Sci Rep 2020;10:21379. 10.1038/s41598-020-78505-w. 33288840 PMC7721695

[267] Guo L Xiong W Liu D . The mncp-spi score predicting risk of severe covid-19 among mild-pneumonia patients on admission. Infect Drug Resist 2020;13:3593-600. 10.2147/IDR.S263157. 33116679 PMC7569081

[268] Gupta RK Harrison EM Ho A ISARIC4C Investigators . Development and validation of the ISARIC 4C Deterioration model for adults hospitalised with COVID-19: a prospective cohort study. Lancet Respir Med 2021;9:349-59. 10.1016/S2213-2600(20)30559-2. 33444539 PMC7832571

[269] Gupta RK Marks M Samuels THA UCLH COVID-19 Reporting Group . Systematic evaluation and external validation of 22 prognostic models among hospitalised adults with COVID-19: an observational cohort study. Eur Respir J 2020;56:2003498. 10.1183/13993003.03498-2020. 32978307 PMC7518075

[270] Hachim MY Hachim IY Naeem KB Hannawi H Salmi IA Hannawi S . D-dimer, Troponin, and Urea Level at Presentation With COVID-19 can Predict ICU Admission: A Single Centered Study. Front Med 2020;7:585003. 10.3389/fmed.2020.585003. 33363185 PMC7756124

[271] Haimovich AD Ravindra NG Stoytchev S . Development and Validation of the Quick COVID-19 Severity Index: A Prognostic Tool for Early Clinical Decompensation. Ann Emerg Med 2020;76:442-53. 10.1016/j.annemergmed.2020.07.022. 33012378 PMC7373004

[272] Hajifathalian K Sharaiha RZ Kumar S . Development and external validation of a prediction risk model for short-term mortality among hospitalized U.S. COVID-19 patients: A proposal for the COVID-AID risk tool. PLoS One 2020;15:e0239536. 10.1371/journal.pone.0239536. 32997700 PMC7526907

[273] Halalau A Imam Z Karabon P . External validation of a clinical risk score to predict hospital admission and in-hospital mortality in COVID-19 patients. Ann Med 2021;53:78-86. 10.1080/07853890.2020.1828616. 32997542 PMC7877986

[274] Hao B Sotudian S Wang T . Early prediction of level-of-care requirements in patients with COVID-19. Elife 2020;9:e60519. 10.7554/eLife.60519. 33044170 PMC7595731

[275] He L Zhang Q Li Z . Incorporation of urinary neutrophil gelatinase-Associated lipocalin and computed tomography quantification to predict acute kidney injury and in-hospital death in covid-19 patients. Kidney Dis) 2021;7:120-30. 10.1159/000511403. 33824868 PMC7573910

[276] Hectors SJ Riyahi S Dev H Krishnan K Margolis DJA Prince MR . Multivariate analysis of CT imaging, laboratory, and demographical features for prediction of acute kidney injury in COVID-19 patients: a Bi-centric analysis. Abdom Radiol 2021;46:1651-8. 10.1007/s00261-020-02823-w. 33098478 PMC7584857

[277] Heo J Han D Kim HJ . Prediction of patients requiring intensive care for COVID-19: development and validation of an integer-based score using data from Centers for Disease Control and Prevention of South Korea. J Intensive Care 2021;9:16. 10.1186/s40560-021-00527-x. 33514443 PMC7844778

[278] Heo J Park JA Han D . A COVID-19 Outcome Prediction and Monitoring Solution for Military Hospitals in South Korea: Development and Evaluation of a Platform. J Med Internet Res 2020;22:e22131. 10.2196/22131. 33048824 PMC7644266

[279] Ho TT Park J Kim T . Deep Learning Models for Predicting Severe Progression in COVID-19-Infected Patients: Retrospective Study. JMIR Med Inform 2021;9:e24973. 10.2196/24973. 33455900 PMC7850779

[280] Holten AR Nore KG Tveiten CEVWK Olasveengen TM Tonby K . Predicting severe COVID-19 in the Emergency Department. Resusc Plus 2020;4:100042. 10.1016/j.resplu.2020.100042. 33403367 PMC7577659

[281] Hu H Yao N Qiu Y . Predictive Value of 5 Early Warning Scores for Critical COVID-19 Patients. Disaster Med Public Health Prep 2022;16:232-9. 10.1017/dmp.2020.324. 32900406 PMC7596567

[282] Hu X Deng H Wang Y Chen L Gu X Wang X . Predictive value of the prognostic nutritional index for the severity of coronavirus disease 2019. Nutrition 2021;84:111123. 10.1016/j.nut.2020.111123. 33476998 PMC7747677

[283] Huang D Yang H Yu H Wang T Yao R Liang Z . A novel risk score to predict cardiovascular complications in patients with coronavirus disease 2019 (COVID-19): A retrospective, multicenter, observational study. Immun Inflamm Dis 2020;8:638-49. 10.1002/iid3.353. 32969605 PMC7537545

[284] Huang J Zheng L Li Z . Kinetics of SARS-CoV-2 positivity of infected and recovered patients from a single center. Sci Rep 2020;10:18629. 10.1038/s41598-020-75629-x. 33122706 PMC7596704

[285] Iijima Y Okamoto T Shirai T . MuLBSTA score is a useful tool for predicting COVID-19 disease behavior. J Infect Chemother 2021;27:284-90. 10.1016/j.jiac.2020.10.013. 33129694 PMC7552979

[286] Ikemura K Bellin E Yagi Y . Using Automated Machine Learning to Predict the Mortality of Patients With COVID-19: Prediction Model Development Study. J Med Internet Res 2021;23:e23458. 10.2196/23458. 33539308 PMC7919846

[287] Jain AC Kansal S Sardana R Bali RK Kar S Chawla R . A retrospective observational study to determine the early predictors of in-hospital mortality at admission with covid-19. Indian J Crit Care Med 2020;24:1174-9. 10.5005/jp-journals-10071-23683. 33446968 PMC7775949

[288] Jamal MH Doi SA AlYouha S . A biomarker based severity progression indicator for COVID-19: the Kuwait prognosis indicator score. Biomarkers 2020;25:641-8. 10.1080/1354750X.2020.1841296. 33090050 PMC7711740

[289] Jang JG Hur J Hong KS Lee W Ahn JH . Prognostic Accuracy of the SIRS, qSOFA, and NEWS for Early Detection of Clinical Deterioration in SARS-CoV-2 Infected Patients. J Korean Med Sci 2020;35:e234. 10.3346/jkms.2020.35.e234. 32597046 PMC7324266

[290] Jehi L Ji X Milinovich A . Development and validation of a model for individualized prediction of hospitalization risk in 4,536 patients with COVID-19. PLoS One 2020;15:e0237419. 10.1371/journal.pone.0237419. 32780765 PMC7418996

[291] Jimenez-Solem E Petersen TS Hansen C . Developing and validating COVID-19 adverse outcome risk prediction models from a bi-national European cohort of 5594 patients. Sci Rep 2021;11:3246. 10.1038/s41598-021-81844-x. 33547335 PMC7864944

[292] Kaeuffer C Ruch Y Fabacher T COVID-19 Alsace Study Group . The BAS^2^IC Score: A Useful Tool to Identify Patients at High Risk of Early Progression to Severe Coronavirus Disease 2019. Open Forum Infect Dis 2020;7:a405. 10.1093/ofid/ofaa405 33274248 PMC7499730

[293] Kamran SM Mirza ZE Moeed HA . CALL Score and RAS Score as Predictive Models for Coronavirus Disease 2019. Cureus 2020;12:e11368. 10.7759/cureus.11368. 33304701 PMC7721080

[294] Kang J Chen T Luo H Luo Y Du G Jiming-Yang M . Machine learning predictive model for severe COVID-19. Infect Genet Evol 2021;90:104737. 10.1016/j.meegid.2021.104737. 33515712 PMC7840410

[295] Kattan MW Ji X Milinovich A . An Algorithm for Classifying Patients Most Likely to Develop Severe Coronavirus Disease 2019 Illness. Crit Care Explor 2020;2:e0300. 10.1097/CCE.0000000000000300. 33354674 PMC7746202

[296] Kim HJ Han D Kim JH . An Easy-to-Use Machine Learning Model to Predict the Prognosis of Patients With COVID-19: Retrospective Cohort Study. J Med Internet Res 2020;22:e24225. 10.2196/24225. 33108316 PMC7655730

[297] Kimura-Sandoval Y Arévalo-Molina ME Cristancho-Rojas CN . Validation of Chest Computed Tomography Artificial Intelligence to Determine the Requirement for Mechanical Ventilation and Risk of Mortality in Hospitalized Coronavirus Disease-19 Patients in a Tertiary Care Center In Mexico City. Rev Invest Clin 2020. 10.24875/RIC.20000451. 33201872

[298] King JT Jr Yoon JS Rentsch CT . Development and validation of a 30-day mortality index based on pre-existing medical administrative data from 13,323 COVID-19 patients: The Veterans Health Administration COVID-19 (VACO) Index. PLoS One 2020;15:e0241825. 10.1371/journal.pone.0241825. 33175863 PMC7657526

[299] Kirsch B Aziz M Kumar S . Wells Score to Predict Pulmonary Embolism in Patients with Coronavirus Disease 2019. Am J Med 2021;134:688-90. 10.1016/j.amjmed.2020.10.044. 33316254 PMC7732230

[300] Kivrak M Guldogan E Colak C . Prediction of death status on the course of treatment in SARS-COV-2 patients with deep learning and machine learning methods. Comput Methods Programs Biomed 2021;201:105951. 10.1016/j.cmpb.2021.105951. 33513487 PMC7826038

[301] Ko H Chung H Kang WS . An Artificial Intelligence Model to Predict the Mortality of COVID-19 Patients at Hospital Admission Time Using Routine Blood Samples: Development and Validation of an Ensemble Model. J Med Internet Res 2020;22:e25442. 10.2196/25442. 33301414 PMC7759509

[302] Kodama T Obinata H Mori H . Prediction of an increase in oxygen requirement of SARS-CoV-2 pneumonia using three different scoring systems. J Infect Chemother 2021;27:336-41. 10.1016/j.jiac.2020.12.009. 33402303 PMC7833485

[303] Kostakis I Smith GB Prytherch D Meredith P Price C Chauhan A Portsmouth Academic ConsortIum For Investigating COVID-19 (PACIFIC-19) . The performance of the National Early Warning Score and National Early Warning Score 2 in hospitalised patients infected by the severe acute respiratory syndrome coronavirus 2 (SARS-CoV-2). Resuscitation 2021;159:150-7. 10.1016/j.resuscitation.2020.10.039. 33176170 PMC7648887

[304] Laguna-Goya R Utrero-Rico A Talayero P . IL-6-based mortality risk model for hospitalized patients with COVID-19. J Allergy Clin Immunol 2020;146:799-807.e9. 10.1016/j.jaci.2020.07.009. 32710975 PMC7375283

[305] Lasbleiz A Cariou B Darmon P . Phenotypic characteristics and development of a hospitalization prediction risk score for outpatients with diabetes and covid-19: The diabcovid study. J Clin Med 2020;9:E3726. 10.3390/jcm9113726. 33233575 PMC7699790

[306] Lassau N Ammari S Chouzenoux E . Integrating deep learning CT-scan model, biological and clinical variables to predict severity of COVID-19 patients. Nat Commun 2021;12:634. 10.1038/s41467-020-20657-4. 33504775 PMC7840774

[307] Levine DM Lipsitz SR Co Z Song W Dykes PC Samal L . Derivation of a Clinical Risk Score to Predict 14-Day Occurrence of Hypoxia, ICU Admission, and Death Among Patients with Coronavirus Disease 2019. J Gen Intern Med 2021;36:730-7. 10.1007/s11606-020-06353-5. 33274414 PMC7713904

[308] Li J Chen Y Chen S . Derivation and validation of a prognostic model for predicting in-hospital mortality in patients admitted with COVID-19 in Wuhan, China: the PLANS (platelet lymphocyte age neutrophil sex) model. BMC Infect Dis 2020;20:959. 10.1186/s12879-020-05688-y. 33334318 PMC7744735

[309] Li JY Wang HF Yin P Thrombo-COVID-19 Collaborative . Clinical characteristics and risk factors for symptomatic venous thromboembolism in hospitalized COVID-19 patients: A multicenter retrospective study. J Thromb Haemost 2021;19:1038-48. 10.1111/jth.15261. 33534149 PMC8014692

[310] Li L Fang X Cheng L . Development and validation of a prognostic nomogram for predicting in-hospital mortality of COVID-19: a multicenter retrospective cohort study of 4086 cases in China. Aging (Albany NY) 2021;13:3176-89. 10.18632/aging.202605. 33561834 PMC7906167

[311] Li Q Zhang T Li F . Acute Kidney Injury Can Predict In-Hospital Mortality in Elderly Patients with COVID-19 in the ICU: A Single-Center Study. Clin Interv Aging 2020;15:2095-107. 10.2147/CIA.S273720. 33204075 PMC7666828

[312] Li S Lin Y Zhu T . Development and external evaluation of predictions models for mortality of COVID-19 patients using machine learning method. Neural Comput Appl 2021;1-10. 10.1007/s00521-020-05592-1. 33424133 PMC7783503

[313] Li X Ge P Zhu J . Deep learning prediction of likelihood of ICU admission and mortality in COVID-19 patients using clinical variables. PeerJ 2020;8:e10337. 10.7717/peerj.10337. 33194455 PMC7651477

[314] Li Y Horowitz MA Liu J . Individual-Level Fatality Prediction of COVID-19 Patients Using AI Methods. Front Public Health 2020;8:587937. 10.3389/fpubh.2020.587937. 33102426 PMC7556112

[315] Liang M He M Tang J . Novel risk scoring system for predicting acute respiratory distress syndrome among hospitalized patients with coronavirus disease 2019 in Wuhan, China. BMC Infect Dis 2020;20:960. 10.1186/s12879-020-05561-y. 33334314 PMC7744733

[316] Liang W Yao J Chen A . Early triage of critically ill COVID-19 patients using deep learning. Nat Commun 2020;11:3543. 10.1038/s41467-020-17280-8. 32669540 PMC7363899

[317] Linssen J Ermens A Berrevoets M . A novel haemocytometric COVID-19 prognostic score developed and validated in an observational multicentre European hospital-based study. Elife 2020;9:e63195. 10.7554/eLife.63195. 33241996 PMC7732342

[318] Liu C Li L Song K . A nomogram for predicting mortality in patients with COVID-19 and solid tumors: a multicenter retrospective cohort study. J Immunother Cancer 2020;8:e001314. 10.1136/jitc-2020-001314. 32895296 PMC7476423

[319] Liu FY Sun XL Zhang Y . Evaluation of the Risk Prediction Tools for Patients With Coronavirus Disease 2019 in Wuhan, China: A Single-Centered, Retrospective, Observational Study. Crit Care Med 2020;48:e1004-11. 10.1097/CCM.0000000000004549. 32897668 PMC7448719

[320] Liu H Chen J Yang Q . Development and validation of a risk score using complete blood count to predict in-hospital mortality in COVID-19 patients. Med (N Y) 2021;2:435-447.e4. 10.1016/j.medj.2020.12.013. 33521746 PMC7831644

[321] Liu J Tao L Gao Z Jiang R Liu M . Development and validation of a prediction model for early identification of critically ill elderly COVID-19 patients. Aging (Albany NY) 2020;12:18822-32. 10.18632/aging.103716. 33024057 PMC7732309

[322] Liu L Chen Z Du Y . CD8^+^ T cells predicted the conversion of common covid-19 to severe. Sci Rep 2021;11:2169. 10.1038/s41598-021-81732-4. 33500507 PMC7838185

[323] Liu L Xie J Wu W . A simple nomogram for predicting failure of non-invasive respiratory strategies in adults with COVID-19: a retrospective multicentre study. Lancet Digit Health 2021;3:e166-74. 10.1016/S2589-7500(20)30316-2. 33573999 PMC7906717

[324] Liu Q Wang Y Zhao X . Diagnostic Performance of a Blood Urea Nitrogen to Creatinine Ratio-based Nomogram for Predicting In-hospital Mortality in COVID-19 Patients. Risk Manag Healthc Policy 2021;14:117-28. 10.2147/RMHP.S278365. 33469395 PMC7811470

[325] Liu S Yao N Qiu Y He C . Predictive performance of SOFA and qSOFA for in-hospital mortality in severe novel coronavirus disease. Am J Emerg Med 2020;38:2074-80. 10.1016/j.ajem.2020.07.019. 33142178 PMC7354270

[326] Lorente L Gómez-Bernal F Martín MM Working Group on COVID-19 Canary ICU . High serum nitrates levels in non-survivor COVID-19 patients. Med Intensiva (Engl Ed) 2020;S0210-5691(20)30336-3. 10.1016/j.medin.2020.10.003. 33293102 PMC7654288

[327] Lundon DJ Kelly BD Shukla D Bolton DM Wiklund P Tewari A . A decision aide for the risk stratification of gu cancer patients at risk of SARS-CoV-2 infection, COVID-19 related hospitalization, intubation, and mortality. J Clin Med 2020;9:9. 10.3390/jcm9092799 32872607 PMC7563697

[328] Luo Y Mao L Yuan X . Prediction Model Based on the Combination of Cytokines and Lymphocyte Subsets for Prognosis of SARS-CoV-2 Infection. J Clin Immunol 2020;40:960-9. 10.1007/s10875-020-00821-7. 32661797 PMC7357264

[329] Ma B Gong J Yang Y Yao X Deng X Chen X . Applicability of MuLBSTA scoring system as diagnostic and prognostic role in early warning of severe COVID-19. Microb Pathog 2021;150:104706. 10.1016/j.micpath.2020.104706. 33347962 PMC7758722

[330] Ma X Li A Jiao M . Characteristic of 523 COVID-19 in Henan Province and a Death Prediction Model. Front Public Health 2020;8:475. 10.3389/fpubh.2020.00475. 33014973 PMC7506160

[331] Ma X Wang H Huang J . A nomogramic model based on clinical and laboratory parameters at admission for predicting the survival of COVID-19 patients. BMC Infect Dis 2020;20:899. 10.1186/s12879-020-05614-2. 33256643 PMC7702207

[332] Magro B Zuccaro V Novelli L . Predicting in-hospital mortality from Coronavirus Disease 2019: A simple validated app for clinical use. PLoS One 2021;16:e0245281. 10.1371/journal.pone.0245281. 33444411 PMC7808616

[333] Manocha KK Kirzner J Ying X . Troponin and Other Biomarker Levels and Outcomes Among Patients Hospitalized With COVID-19: Derivation and Validation of the HA_2_T_2_ COVID-19 Mortality Risk Score. J Am Heart Assoc 2021;10:e018477. 10.1161/JAHA.120.018477. 33121304 PMC8174190

[334] McElvaney OJ Hobbs BD Qiao D . A linear prognostic score based on the ratio of interleukin-6 to interleukin-10 predicts outcomes in COVID-19. EBioMedicine 2020;61:103026. 10.1016/j.ebiom.2020.103026. 33039714 PMC7543971

[335] McRae MP Dapkins IP Sharif I . Managing COVID-19 With a Clinical Decision Support Tool in a Community Health Network: Algorithm Development and Validation. J Med Internet Res 2020;22:e22033. 10.2196/22033. 32750010 PMC7446714

[336] Mei J Hu W Chen Q . Development and external validation of a COVID-19 mortality risk prediction algorithm: a multicentre retrospective cohort study. BMJ Open 2020;10:e044028. 10.1136/bmjopen-2020-044028. 33361083 PMC7768618

[337] Mei Q Wang AY Bryant A . Development and validation of prognostic model for predicting mortality of COVID-19 patients in Wuhan, China. Sci Rep 2020;10:22451. 10.1038/s41598-020-78870-6. 33384422 PMC7775455

[338] Mejía-Vilet JM Córdova-Sánchez BM Fernández-Camargo DA Méndez-Pérez RA Morales-Buenrostro LE Hernández-Gilsoul T . A risk score to predict admission to the intensive care unit in patients with Covid-19: the ABC-GOALS score. Salud Publica Mex 2020;63(1, ene-feb):1-11. 10.21149/11684. 33021362

[339] Muhammad LJ Islam MM Usman SS Ayon SI . Predictive Data Mining Models for Novel Coronavirus (COVID-19) Infected Patients’ Recovery. SN Comput Sci 2020;1:206. 10.1007/s42979-020-00216-w. 33063049 PMC7306186

[340] Myrstad M Ihle-Hansen H Tveita AA . National Early Warning Score 2 (NEWS2) on admission predicts severe disease and in-hospital mortality from Covid-19 - a prospective cohort study. Scand J Trauma Resusc Emerg Med 2020;28:66. 10.1186/s13049-020-00764-3. 32660623 PMC7356106

[341] Nagant C Ponthieux F Smet J . A score combining early detection of cytokines accurately predicts COVID-19 severity and intensive care unit transfer. Int J Infect Dis 2020;101:342-5. 10.1016/j.ijid.2020.10.003. 33039609 PMC7544772

[342] Nemati M Ansary J Nemati N . Machine-Learning Approaches in COVID-19 Survival Analysis and Discharge-Time Likelihood Prediction Using Clinical Data. Patterns (N Y) 2020;1:100074. 10.1016/j.patter.2020.100074. 32835314 PMC7334917

[343] Nguyen Y Corre F Honsel V . A nomogram to predict the risk of unfavourable outcome in COVID-19: a retrospective cohort of 279 hospitalized patients in Paris area. Ann Med 2020;52:367-75. 10.1080/07853890.2020.1803499. 32723107 PMC7877983

[344] Nicholson CJ Wooster L Sigurslid HH . Estimating Risk of Mechanical Ventilation and Mortality Among Adult COVID-19 patients Admitted to Mass General Brigham: The VICE and DICE Scores. medRxiv 2020 10.1101/2020.09.14.20194670 PMC7906522 33655204

[345] Ning W Lei S Yang J . Open resource of clinical data from patients with pneumonia for the prediction of COVID-19 outcomes via deep learning. Nat Biomed Eng 2020;4:1197-207. 10.1038/s41551-020-00633-5. 33208927 PMC7723858

[346] Niu Y Zhan Z Li J . Development of a predictive model for mortality in hospitalized patients with COVID-19. Disaster Med Public Health Prep 2021;1-9. 10.1017/dmp.2021.320 33413721 PMC8007955

[347] Núñez-Gil IJ Fernández-Pérez C Estrada V HOPE COVID-19 Investigators . Mortality risk assessment in Spain and Italy, insights of the HOPE COVID-19 registry. Intern Emerg Med 2021;16:957-66. 10.1007/s11739-020-02543-5. 33165755 PMC7649104

[348] Pan D Cheng D Cao Y . A Predicting Nomogram for Mortality in Patients With COVID-19. Front Public Health 2020;8:461. 10.3389/fpubh.2020.00461. 32850612 PMC7432145

[349] Pan P Li Y Xiao Y . Prognostic Assessment of COVID-19 in the Intensive Care Unit by Machine Learning Methods: Model Development and Validation. J Med Internet Res 2020;22:e23128. 10.2196/23128. 33035175 PMC7661105

[350] Parchure P Joshi H Dharmarajan K . Development and validation of a machine learning-based prediction model for near-term in-hospital mortality among patients with COVID-19. BMJ Support Palliat Care 2020:bmjspcare-2020-002602. 10.1136/bmjspcare-2020-002602. 32963059 PMC8049537

[351] Payán-Pernía S Gómez Pérez L Remacha Sevilla ÁF Sierra Gil J Novelli Canales S . Absolute Lymphocytes, Ferritin, C-Reactive Protein, and Lactate Dehydrogenase Predict Early Invasive Ventilation in Patients With COVID-19. Lab Med 2021;52:141-5. 10.1093/labmed/lmaa105. 33336243 PMC7798986

[352] Qin S Li W Shi X . 3044 Cases reveal important prognosis signatures of COVID-19 patients. Comput Struct Biotechnol J 2021;19:1163-75. 10.1016/j.csbj.2021.01.042. 33584997 PMC7870437

[353] Qin ZJ Liu L Sun Q . Impaired immune and coagulation systems may be early risk factors for COVID-19 patients: A retrospective study of 118 inpatients from Wuhan, China. Medicine (Baltimore) 2020;99:e21700. 10.1097/MD.0000000000021700. 32871887 PMC7458161

[354] Quiroz JC Feng YZ Cheng ZY . Development and Validation of a Machine Learning Approach for Automated Severity Assessment of COVID-19 Based on Clinical and Imaging Data: Retrospective Study. JMIR Med Inform 2021;9:e24572. 10.2196/24572. 33534723 PMC7879715

[355] Razavian N Major VJ Sudarshan M . A validated, real-time prediction model for favorable outcomes in hospitalized COVID-19 patients. NPJ Digit Med 2020;3:130. 10.1038/s41746-020-00343-x. 33083565 PMC7538971

[356] Rechtman E Curtin P Navarro E Nirenberg S Horton MK . Vital signs assessed in initial clinical encounters predict COVID-19 mortality in an NYC hospital system. Sci Rep 2020;10:21545. 10.1038/s41598-020-78392-1. 33298991 PMC7726000

[357] Riveiro-Barciela M Labrador-Horrillo M Camps-Relats L . Simple predictive models identify patients with COVID-19 pneumonia and poor prognosis. PLoS One 2020;15:e0244627. 10.1371/journal.pone.0244627. 33370397 PMC7769554

[358] Rodriguez-Nava G Yanez-Bello MA Trelles-Garcia DP Chung CW Friedman HJ Hines DW . Performance of the quick COVID-19 severity index and the Brescia-COVID respiratory severity scale in hospitalized patients with COVID-19 in a community hospital setting. Int J Infect Dis 2021;102:571-6. 10.1016/j.ijid.2020.11.003. 33181332 PMC7833674

[359] Roimi M Gutman R Somer J . Development and validation of a machine learning model predicting illness trajectory and hospital utilization of COVID-19 patients: A nationwide study. J Am Med Inform Assoc 2021;28:1188-96. 10.1093/jamia/ocab005. 33479727 PMC7928913

[360] Ruocco G McCullough PA Tecson KM . Mortality Risk Assessment Using CHA(2)DS(2)-VASc Scores in Patients Hospitalized With Coronavirus Disease 2019 Infection. Am J Cardiol 2020;137:111-7. 10.1016/j.amjcard.2020.09.029. 32991860 PMC7521434

[361] Ryan C Minc A Caceres J . Predicting severe outcomes in Covid-19 related illness using only patient demographics, comorbidities and symptoms. Am J Emerg Med 2021;45:378-84. 10.1016/j.ajem.2020.09.017. 33046294 PMC7480533

[362] Ryan L Lam C Mataraso S . Mortality prediction model for the triage of COVID-19, pneumonia, and mechanically ventilated ICU patients: A retrospective study. Ann Med Surg (Lond) 2020;59:207-16. 10.1016/j.amsu.2020.09.044. 33042536 PMC7532803

[363] Saberian P Tavakoli N Hasani-Sharamin P Modabber M Jamshididana M Baratloo A . Accuracy of the pre-hospital triage tools (qSOFA, NEWS, and PRESEP) in predicting probable COVID-19 patients’ outcomes transferred by Emergency Medical Services. Caspian J Intern Med 2020;11(Suppl 1):536-43. 10.22088/cjim.11.0.536. 33425272 PMC7780871

[364] Salahshour F Mehrabinejad MM Nassiri Toosi M . Clinical and chest CT features as a predictive tool for COVID-19 clinical progress: introducing a novel semi-quantitative scoring system. Eur Radiol 2021;31:5178-88. 10.1007/s00330-020-07623-w. 33449185 PMC7809225

[365] Sánchez-Montañés M Rodríguez-Belenguer P Serrano-López AJ Soria-Olivas E Alakhdar-Mohmara Y . Machine learning for mortality analysis in patients with COVID-19. Int J Environ Res Public Health 2020;17:E8386. 10.3390/ijerph17228386. 33198392 PMC7697463

[366] Sang S Sun R Coquet J Carmichael H Seto T Hernandez-Boussard T . Learning From Past Respiratory Infections to Predict COVID-19 Outcomes: Retrospective Study. J Med Internet Res 2021;23:e23026. 10.2196/23026. 33534724 PMC7901593

[367] Saridaki M Metallidis S Grigoropoulou S . Integration of heparin-binding protein and interleukin-6 in the early prediction of respiratory failure and mortality in pneumonia by SARS-CoV-2 (COVID-19). Eur J Clin Microbiol Infect Dis 2021;40:1405-12. 10.1007/s10096-020-04145-7. 33515095 PMC7846268

[368] Schalekamp S Huisman M van Dijk RA . Model-based Prediction of Critical Illness in Hospitalized Patients with COVID-19. Radiology 2021;298:E46-54. 10.1148/radiol.2020202723. 32787701 PMC7427120

[369] Schöning V Liakoni E Baumgartner C . Development and validation of a prognostic COVID-19 severity assessment (COSA) score and machine learning models for patient triage at a tertiary hospital. J Transl Med 2021;19:56. 10.1186/s12967-021-02720-w. 33546711 PMC7862984

[370] Sensusiati AD Amin M Nasronudin N . Age, neutrophil lymphocyte ratio, and radiographic assessment of the quantity of lung edema (RALE) score to predict in-hospital mortality in COVID-19 patients: a retrospective study. F1000Res 2020;9:1286. 10.12688/f1000research.26723.1. 33537125 PMC7836085

[371] Shang Y Liu T Wei Y . Scoring systems for predicting mortality for severe patients with COVID-19. EClinicalMedicine 2020;24:100426. 10.1016/j.eclinm.2020.100426. 32766541 PMC7332889

[372] Sharp AL Huang BZ Broder B . Identifying patients with symptoms suspicious for COVID-19 at elevated risk of adverse events: The COVAS score. Am J Emerg Med 2021;46:489-94. 10.1016/j.ajem.2020.10.068. 33189516 PMC7642742

[373] Shashikumar SP Wardi G Paul P . Development and Prospective Validation of a Deep Learning Algorithm for Predicting Need for Mechanical Ventilation. Chest 2021;159:2264-73. 10.1016/j.chest.2020.12.009. 33345948 PMC8027289

[374] Shi Y Pandita A Hardesty A . Validation of pneumonia prognostic scores in a statewide cohort of hospitalised patients with COVID-19. Int J Clin Pract 2021;75:e13926. 10.1111/ijcp.13926. 33296132 PMC7883205

[375] Shoer S Karady T Keshet A . A Prediction Model to Prioritize Individuals for a SARS-CoV-2 Test Built from National Symptom Surveys. Med (N Y) 2021;2:196-208.e4. 10.1016/j.medj.2020.10.002. 33073258 PMC7547576

[376] Song C Dong Z Gong H . An online tool for predicting the prognosis of cancer patients with SARS-CoV-2 infection: a multi-center study. J Cancer Res Clin Oncol 2021;147:1247-57. 10.1007/s00432-020-03420-6. 33040189 PMC7548053

[377] Soto-Mota A Marfil-Garza BA Martínez R . The low-harm score for predicting mortality in patients diagnosed with COVID-19: A multicentric validation study. J Am Coll Emerg Physicians Open, 2020, 10.1002/emp2.12259. PMC7675373 33230506

[378] Sourij H Aziz F Bräuer A COVID-19 in diabetes in Austria study group . COVID-19 fatality prediction in people with diabetes and prediabetes using a simple score upon hospital admission. Diabetes Obes Metab 2021;23:589-98. 10.1111/dom.14256. 33200501 PMC7753560

[379] Sun C Hong S Song M Li H Wang Z . Predicting COVID-19 disease progression and patient outcomes based on temporal deep learning. BMC Med Inform Decis Mak 2021;21:45. 10.1186/s12911-020-01359-9. 33557818 PMC7869774

[380] Sun H Jain A Leone MJ . CoVA: An Acuity Score for Outpatient Screening that Predicts Coronavirus Disease 2019 Prognosis. J Infect Dis 2021;223:38-46. 10.1093/infdis/jiaa663. 33098643 PMC7665643

[381] Tabatabai A Ghneim MH Kaczorowski DJ . Mortality Risk Assessment in COVID-19 Venovenous Extracorporeal Membrane Oxygenation. Ann Thorac Surg 2021;112:1983-9. 10.1016/j.athoracsur.2020.12.050. 33485917 PMC7825896

[382] Tanboğa IH Canpolat U Çetin EHÖ . Development and validation of clinical prediction model to estimate the probability of death in hospitalized patients with COVID-19: Insights from a nationwide database. J Med Virol 2021;93:3015-22. 10.1002/jmv.26844. 33527474 PMC8014660

[383] Toh C Brody JP . Evaluation of a genetic risk score for severity of COVID-19 using human chromosomal-scale length variation. Hum Genomics 2020;14:36. 10.1186/s40246-020-00288-y. 33036646 PMC7546598

[384] Torres-Macho J Ryan P Valencia J . The PANDEMYC Score. An Easily Applicable and Interpretable Model for Predicting Mortality Associated With COVID-19. J Clin Med 2020;9:E3066. 10.3390/jcm9103066. 32977606 PMC7598151

[385] Tsui ELH Lui CSM Woo PPS . Development of a data-driven COVID-19 prognostication tool to inform triage and step-down care for hospitalised patients in Hong Kong: a population-based cohort study. BMC Med Inform Decis Mak 2020;20:323. 10.1186/s12911-020-01338-0. 33287804 PMC7719738

[386] Vaid A Jaladanki SK Xu J . Federated Learning of Electronic Health Records to Improve Mortality Prediction in Hospitalized Patients With COVID-19: Machine Learning Approach. JMIR Med Inform 2021;9:e24207. 10.2196/24207. 33400679 PMC7842859

[387] van Dam PM Zelis N Stassen P . Validating the RISE UP score for predicting prognosis in patients with COVID-19 in the emergency department: a retrospective study. BMJ Open 2021;11:e045141. 10.1136/bmjopen-2020-045141. 33550267 PMC7925864

[388] van de Sande D van Genderen ME Rosman B . Predicting thromboembolic complications in COVID-19 ICU patients using machine learning. J Clin Transl Res 2020;6:179-86. 33501388 PMC7821745

[389] Varol Y Hakoglu B Kadri Cirak A COVID Study Group . The impact of charlson comorbidity index on mortality from SARS-CoV-2 virus infection and A novel COVID-19 mortality index: CoLACD. Int J Clin Pract 2021;75:e13858. 10.1111/ijcp.13858. 33237615 PMC7744887

[390] Vizcaychipi MP Shovlin CL McCarthy A ChelWest COVID-19 Consortium . Development and implementation of a COVID-19 near real-time traffic light system in an acute hospital setting. Emerg Med J 2020;37:630-6. 10.1136/emermed-2020-210199. 32948623

[391] Wang B Zhong F Zhang H An W Liao M Cao Y . Risk Factor Analysis and Nomogram Construction for Non-Survivors among Critical Patients with COVID-19. Jpn J Infect Dis 2020;73:452-8. 10.7883/yoken.JJID.2020.227. 32611979

[392] Wang J Zhang H Qiao R . Thrombo-inflammatory features predicting mortality in patients with COVID-19: The FAD-85 score. J Int Med Res 2020;48:300060520955037. 10.1177/0300060520955037 32960106 PMC7511832

[393] Wang L Lv Q Zhang X . The utility of MEWS for predicting the mortality in the elderly adults with COVID-19: a retrospective cohort study with comparison to other predictive clinical scores. PeerJ 2020;8:e10018. 10.7717/peerj.10018. 33062437 PMC7528814

[394] Wang P Sha J Meng M . Risk factors for severe COVID-19 in middle-aged patients without comorbidities: a multicentre retrospective study. J Transl Med 2020;18:461. 10.1186/s12967-020-02655-8. 33287826 PMC7719726

[395] Wang R He M Yin W . The Prognostic Nutritional Index is associated with mortality of COVID-19 patients in Wuhan, China. J Clin Lab Anal 2020;34:e23566. 10.1002/jcla.23566. 32914892 PMC7595894

[396] Wang T Paschalidis A Liu Q Liu Y Yuan Y Paschalidis IC . Predictive Models of Mortality for Hospitalized Patients With COVID-19: Retrospective Cohort Study. JMIR Med Inform 2020;8:e21788. 10.2196/21788. 33055061 PMC7572117

[397] Weng Z Chen Q Li S . ANDC: an early warning score to predict mortality risk for patients with Coronavirus Disease 2019. J Transl Med 2020;18:328. 10.1186/s12967-020-02505-7. 32867787 PMC7457219

[398] Wollenstein-Betech S Cassandras CG Paschalidis IC . Personalized predictive models for symptomatic COVID-19 patients using basic preconditions: Hospitalizations, mortality, and the need for an ICU or ventilator. Int J Med Inform 2020;142:104258. 10.1016/j.ijmedinf.2020.104258. 32927229 PMC7442577

[399] Wollenstein-Betech S Silva AAB Fleck JL Cassandras CG Paschalidis IC . Physiological and socioeconomic characteristics predict COVID-19 mortality and resource utilization in Brazil. PLoS One 2020;15:e0240346. 10.1371/journal.pone.0240346. 33052960 PMC7556459

[400] Wu G Yang P Xie Y . Development of a clinical decision support system for severity risk prediction and triage of COVID-19 patients at hospital admission: an international multicentre study. Eur Respir J 2020;56:2001104. 10.1183/13993003.01104-2020. 32616597 PMC7331655

[401] Wu G Zhou S Wang Y . A prediction model of outcome of SARS-CoV-2 pneumonia based on laboratory findings. Sci Rep 2020;10:14042. 10.1038/s41598-020-71114-7. 32820210 PMC7441177

[402] Wu H Zhang H Karwath A . Ensemble learning for poor prognosis predictions: A case study on SARS-CoV-2. J Am Med Inform Assoc 2021;28:791-800. 10.1093/jamia/ocaa295. 33185672 PMC7717299

[403] Wu Q Wang S Li L . Radiomics Analysis of Computed Tomography helps predict poor prognostic outcome in COVID-19. Theranostics 2020;10:7231-44. 10.7150/thno.46428. 32641989 PMC7330838

[404] Xia Y Zhang Y Yuan S . A nomogram to early predict isolation length for non-severe COVID-19 patients based on laboratory investigation: A multicenter retrospective study in Zhejiang Province, China. Clin Chim Acta 2021;512:49-57. 10.1016/j.cca.2020.11.019. 33279501 PMC7836550

[405] Xiao LS Zhang WF Gong MC . Development and validation of the HNC-LL score for predicting the severity of coronavirus disease 2019. EBioMedicine 2020;57:102880. 10.1016/j.ebiom.2020.102880. 32645614 PMC7338276

[406] Xie J Shi D Bao M . A Predictive Nomogram for Predicting Improved Clinical Outcome Probability in Patients with COVID-19 in Zhejiang Province, China. Engineering (Beijing) 2022;8:122-9. 10.1016/j.eng.2020.05.014. 32837744 PMC7274972

[407] Xie L Hou K Xu H . Chest CT features and progression of patients with coronavirus disease 2019. Br J Radiol 2020;93:20200219. 10.1259/bjr.20200219. 33186052 PMC7716016

[408] Xu J Yang X Huang C . A Novel Risk-Stratification Models of the High-Flow Nasal Cannula Therapy in COVID-19 Patients With Hypoxemic Respiratory Failure. Front Med (Lausanne) 2020;7:607821. 10.3389/fmed.2020.607821. 33425951 PMC7793962

[409] Xu R Cui J Hu L . Development and validation of a simplified nomogram predicting individual critical illness of risk in COVID-19: A retrospective study. J Med Virol 2021;93:1999-2009. 10.1002/jmv.26551. 32975825

[410] Xu R Hou K Zhang K . Performance of Two Risk-Stratification Models in Hospitalized Patients With Coronavirus Disease. Front Med (Lausanne) 2020;7:518. 10.3389/fmed.2020.00518. 32923449 PMC7457082

[411] Xue G Gan X Wu Z . Novel serological biomarkers for inflammation in predicting disease severity in patients with COVID-19. Int Immunopharmacol 2020;89(Pt A):107065. 10.1016/j.intimp.2020.107065. 33045571 PMC7532789

[412] Yadaw AS Li YC Bose S Iyengar R Bunyavanich S Pandey G . Clinical features of COVID-19 mortality: development and validation of a clinical prediction model. Lancet Digit Health 2020;2:e516-25. 10.1016/S2589-7500(20)30217-X. 32984797 PMC7508513

[413] Yu J Nie L Wu D . Prognostic Value of a Clinical Biochemistry-Based Nomogram for Coronavirus Disease 2019. Front Med (Lausanne) 2021;7:597791. 10.3389/fmed.2020.597791. 33537326 PMC7848223

[414] Yu Y Wang X Li M . Nomogram to identify severe coronavirus disease 2019 (COVID-19) based on initial clinical and CT characteristics: a multi-center study. BMC Med Imaging 2020;20:111. 10.1186/s12880-020-00513-z. 33008329 PMC7530870

[415] Yu Y Zhu C Yang L . Identification of risk factors for mortality associated with COVID-19. PeerJ 2020;8:e9885. 10.7717/peerj.9885. 32953279 PMC7473053

[416] Yuan Y Sun C Tang X . Development and Validation of a Prognostic Risk Score System for COVID-19 Inpatients: A Multi-Center Retrospective Study in China. Engineering (Beijing) 2022;8:116-21. 10.1016/j.eng.2020.10.013. 33282444 PMC7695569

[417] Zeng F Deng G Cui Y . A predictive model for the severity of COVID-19 in elderly patients. Aging (Albany NY) 2020;12:20982-96. 10.18632/aging.103980. 33170150 PMC7695402

[418] Zhang B Liu Q Zhang X . Clinical Utility of a Nomogram for Predicting 30-Days Poor Outcome in Hospitalized Patients With COVID-19: Multicenter External Validation and Decision Curve Analysis. Front Med (Lausanne) 2020;7:590460. 10.3389/fmed.2020.590460. 33425939 PMC7785751

[419] Zhang S Guo M Duan L . Development and validation of a risk factor-based system to predict short-term survival in adult hospitalized patients with COVID-19: a multicenter, retrospective, cohort study. Crit Care 2020;24:438. 10.1186/s13054-020-03123-x. 32678040 PMC7364297

[420] Zhang X Wang W Wan C . A predictive model for respiratory distress in patients with COVID-19: a retrospective study. Ann Transl Med 2020;8:1585. 10.21037/atm-20-4977. 33437784 PMC7791231

[421] Zhang XY Zhang L Zhao Y Chen L . Risk assessment and prediction of severe or critical covid-19 illness in older adults. Clin Interv Aging 2020;15:2145-53. 10.2147/CIA.S268156. 33204079 PMC7666995

[422] Zhang Y Wu L Yang J Zhou C Liu Y . A nomogram-based prediction for severe pneumonia in patients with coronavirus disease 2019 (COVID-19). Infect Drug Resist 2020;13:3575-82. 10.2147/IDR.S261725. 33116677 PMC7567552

[423] Zhao Y Wang F Dong G Sheng Q Feng S . A disease progression prediction model and nervous system symptoms in coronavirus disease 2019 patients. Am J Transl Res 2020;12:8192-207. 33437392 PMC7791519

[424] Zhao Z Chen A Hou W . Prediction model and risk scores of ICU admission and mortality in COVID-19. PLoS One 2020;15:e0236618. 10.1371/journal.pone.0236618. 32730358 PMC7392248

[425] Zheng Y Xiao A Yu X . Development and Validation of a Prognostic Nomogram Based on Clinical and CT Features for Adverse Outcome Prediction in Patients with COVID-19. Korean J Radiol 2020;21:1007-17. 10.3348/kjr.2020.0485. 32677385 PMC7369204

[426] Zheng Y Zhu Y Ji M . A Learning-Based Model to Evaluate Hospitalization Priority in COVID-19 Pandemics. Patterns (N Y) 2020;1:100092. 10.1016/j.patter.2020.100092. 32838344 PMC7396968

[427] Zhou J Huang L Chen J . Clinical features predicting mortality risk in older patients with COVID-19. Curr Med Res Opin 2020;36:1753-9. 10.1080/03007995.2020.1825365. 32945707

[428] Zhou W Qin X Hu X Lu Y Pan J . Prognosis models for severe and critical COVID-19 based on the Charlson and Elixhauser comorbidity indices. Int J Med Sci 2020;17:2257-63. 10.7150/ijms.50007. 32922189 PMC7484649

[429] Zhou Y He Y Yang H . Exploiting an early warning Nomogram for predicting the risk of ICU admission in patients with COVID-19: a multi-center study in China. Scand J Trauma Resusc Emerg Med 2020;28:106. 10.1186/s13049-020-00795-w. 33109234 PMC7590555

[430] Zhu JS Ge P Jiang C . Deep-learning artificial intelligence analysis of clinical variables predicts mortality in COVID-19 patients. J Am Coll Emerg Physicians Open, 2020, 10.1002/emp2.12205. PMC7405082 32838390

[431] The Royal College of Physicians . National Early Warning Score (NEWS) 2: Standardising the assessment of acute-illness severity in the NHS. Updated report of a working party. RCP, 2017.

[432] Carr E Bendayan R O’Gallagher K . Supplementing the National Early Warning Score (NEWS2) for anticipating early deterioration among patients with COVID-19 infection. MedRxiv 2020. 10.1101/2020.04.24.20078006

[433] Unit CCT. TACTIC: Cambridge Clinical Trials Unit; 2020. https://cctu.org.uk/portfolio/COVID-19/TACTIC.

[434] Subbe CP Kruger M Rutherford P Gemmel L . Validation of a modified Early Warning Score in medical admissions. QJM 2001;94:521-6. 10.1093/qjmed/94.10.521. 11588210

[435] Lim WS van der Eerden MM Laing R . Defining community acquired pneumonia severity on presentation to hospital: an international derivation and validation study. Thorax 2003;58:377-82. 10.1136/thorax.58.5.377. 12728155 PMC1746657

[436] Olsson T Terent A Lind L . Rapid Emergency Medicine score: a new prognostic tool for in-hospital mortality in nonsurgical emergency department patients. J Intern Med 2004;255:579-87. 10.1111/j.1365-2796.2004.01321.x. 15078500

[437] Seymour CW Liu VX Iwashyna TJ . Assessment of Clinical Criteria for Sepsis: For the Third International Consensus Definitions for Sepsis and Septic Shock (Sepsis-3). JAMA 2016;315:762-74. 10.1001/jama.2016.0288. 26903335 PMC5433435

[438] Riley RD Ensor J Snell KIE . Calculating the sample size required for developing a clinical prediction model. BMJ 2020;368:m441. 10.1136/bmj.m441 32188600

[439] Riley RD Collins GS Ensor J . Minimum sample size calculations for external validation of a clinical prediction model with a time-to-event outcome. Stat Med 2022;41:1280-95. 10.1002/sim.9275. 34915593

[440] Riley RD Debray TPA Collins GS . Minimum sample size for external validation of a clinical prediction model with a binary outcome. Stat Med 2021;40:4230-51. 10.1002/sim.9025. 34031906

[441] Riley RD Ensor J Snell KIE . Calculating the sample size required for developing a clinical prediction model. BMJ 2020;368:m441. 10.1136/bmj.m441. 32188600

[442] Pavlou M Qu C Omar RZ . Estimation of required sample size for external validation of risk models for binary outcomes. Stat Methods Med Res 2021;30:2187-206. 10.1177/09622802211007522. 33881369 PMC8529102

[443] Gravesteijn BY Sewalt CA Venema E Nieboer D Steyerberg EW CENTER-TBI Collaborators . Missing Data in Prediction Research: A Five-Step Approach for Multiple Imputation, Illustrated in the CENTER-TBI Study. J Neurotrauma 2021;38:1842-57. 10.1089/neu.2020.7218. 33470157

[444] Vergouw D Heymans MW van der Windt DA . Missing data and imputation: a practical illustration in a prognostic study on low back pain. J Manipulative Physiol Ther 2012;35:464-71. 10.1016/j.jmpt.2012.07.002. 22964020

[445] van Smeden M Moons KG de Groot JA . Sample size for binary logistic prediction models: beyond events per variable criteria. Stat Methods Med Res 2019;28:2455-74. 10.1177/0962280218784726 29966490 PMC6710621

[446] Steyerberg EW Harrell FE Jr . Prediction models need appropriate internal, internal-external, and external validation. J Clin Epidemiol 2016;69:245-7. 10.1016/j.jclinepi.2015.04.005 25981519 PMC5578404

[447] Van Calster B McLernon DJ van Smeden M Wynants L Steyerberg EW Topic Group ‘Evaluating diagnostic tests and prediction models’ of the STRATOS initiative . Calibration: the Achilles heel of predictive analytics. BMC Med 2019;17:230. 10.1186/s12916-019-1466-7 31842878 PMC6912996

[448] Austin PC Lee DS Fine JP . Introduction to the analysis of survival data in the presence of competing risks. Circulation 2016;133:601-9. 10.1161/CIRCULATIONAHA.115.017719 26858290 PMC4741409

[449] Riley RD Ensor J Snell KI . External validation of clinical prediction models using big datasets from e-health records or IPD meta-analysis: opportunities and challenges [correction: *BMJ* 2019;365:l4379]. BMJ 2016;353:i3140. 10.1136/bmj.i3140 27334381 PMC4916924

[450] Debray TP Riley RD Rovers MM Reitsma JB Moons KG Cochrane IPD Meta-analysis Methods group . Individual participant data (IPD) meta-analyses of diagnostic and prognostic modeling studies: guidance on their use. PLoS Med 2015;12:e1001886. 10.1371/journal.pmed.1001886 26461078 PMC4603958

[451] Wynants L Kent DM Timmerman D Lundquist CM Van Calster B . Untapped potential of multicenter studies: a review of cardiovascular risk prediction models revealed inappropriate analyses and wide variation in reporting. Diagn Progn Res 2019;3:6. 10.1186/s41512-019-0046-9 31093576 PMC6460661

[452] Wynants L Riley RD Timmerman D Van Calster B . Random-effects meta-analysis of the clinical utility of tests and prediction models. Stat Med 2018;37:2034-52. 10.1002/sim.7653 29575170

[453] Infervision. Infervision launches hashtag#AI-based hashtag#Covid-19 solution in Europe 2020. https://www.linkedin.com/posts/infervision_ai-covid-medicine-activity-6650772755031613440-TqLJ.

[454] Offord C. Surgisphere fallout hits African nonprotfits covid-19 efforts 2020. The Scientist. https://www.the-scientist.com/news-opinion/surgisphere-fallout-hits-african-nonprofits-covid-19-efforts--67617.

[455] Van Calster B Wynants L Timmerman D Steyerberg EW Collins GS . Predictive analytics in health care: how can we know it works? J Am Med Inform Assoc 2019;26:1651-4. 10.1093/jamia/ocz130 31373357 PMC6857503

[456] Lyons J Nafilyan V Akbari A . Validating the QCOVID risk prediction algorithm for risk of mortality from COVID-19 in the adult population in Wales, UK. Int J Popul Data Sci 2022;5:1697. 10.23889/ijpds.v5i4.1697. 35310465 PMC8900650

[457] Nafilyan V Humberstone B Mehta N . An external validation of the QCovid risk prediction algorithm for risk of mortality from COVID-19 in adults: a national validation cohort study in England. Lancet Digit Health 2021;3:e425-33. 10.1016/S2589-7500(21)00080-7. 34049834 PMC8148652

[458] Simpson CR Robertson C Kerr S . External validation of the QCovid risk prediction algorithm for risk of COVID-19 hospitalisation and mortality in adults: national validation cohort study in Scotland. Thorax 2022;77:497-504. 10.1136/thoraxjnl-2021-217580. 34782484 PMC8595052

[459] Wickstrøm KE Vitelli V Carr E . Regional performance variation in external validation of four prediction models for severity of COVID-19 at hospital admission: An observational multi-centre cohort study. PLoS One 2021;16:e0255748-48. 10.1371/journal.pone.0255748. 34432797 PMC8386866

[460] de Jong VMT Rousset RZ Antonio-Villa NE CovidRetro collaboration CAPACITY-COVID consortium . Clinical prediction models for mortality in patients with covid-19: external validation and individual participant data meta-analysis. BMJ 2022;378:e069881. 10.1136/bmj-2021-069881. 35820692 PMC9273913

[461] Van Calster B Vickers AJ . Calibration of risk prediction models: impact on decision-analytic performance. Med Decis Making 2015;35:162-9. 10.1177/0272989X14547233 25155798

[462] Collins GS Ogundimu EO Altman DG . Sample size considerations for the external validation of a multivariable prognostic model: a resampling study. Stat Med 2016;35:214-26. 10.1002/sim.6787 26553135 PMC4738418

[463] Vergouwe Y Steyerberg EW Eijkemans MJ Habbema JD . Substantial effective sample sizes were required for external validation studies of predictive logistic regression models. J Clin Epidemiol 2005;58:475-83. 10.1016/j.jclinepi.2004.06.017 15845334

[464] Mahase E . Covid-19: What do we know about “long covid”? BMJ 2020;370:m2815. 10.1136/bmj.m2815 32665317

[465] Klok FA Boon GJAM Barco S . The post-covid-19 functional status scale: a tool to measure functional status over time after covid-19. Eur Respir J 2020;56:2001494. 10.1183/13993003.01494-2020 32398306 PMC7236834

