# Prediction models for diagnosis and prognosis of covid-19: systematic review and critical appraisal

**DOI:** 10.1136/bmj.m1328

**Published:** 2020-04-07

**Authors:** Laure Wynants, Ben Van Calster, Gary S Collins, Richard D Riley, Georg Heinze, Ewoud Schuit, Marc M J Bonten, Johanna A A Damen, Thomas P A Debray, Maarten De Vos, Paula Dhiman, Maria C Haller, Michael O Harhay, Liesbet Henckaerts, Nina Kreuzberger, Anna Lohmann, Kim Luijken, Jie Ma, Constanza L Andaur Navarro, Johannes B Reitsma, Jamie C Sergeant, Chunhu Shi, Nicole Skoetz, Luc J M Smits, Kym I E Snell, Matthew Sperrin, René Spijker, Ewout W Steyerberg, Toshihiko Takada, Sander M J van Kuijk, Florien S van Royen, Christine Wallisch, Lotty Hooft, Karel G M Moons, Maarten van Smeden

**Affiliations:** 1 Department of Epidemiology, CAPHRI Care and Public Health Research Institute, Maastricht University, Peter Debyeplein 1, 6229 HA Maastricht, Netherlands; 2 Department of Development and Regeneration, KU Leuven, Leuven, Belgium; 3 Department of Biomedical Data Sciences, Leiden University Medical Centre, Leiden, Netherlands; 4 Centre for Statistics in Medicine, Nuffield Department of Orthopaedics, Musculoskeletal Sciences, University of Oxford, Oxford, UK; 5 NIHR Oxford Biomedical Research Centre, John Radcliffe Hospital, Oxford, UK; 6 Centre for Prognosis Research, School of Primary, Community and Social Care, Keele University, Keele, UK; 7 Section for Clinical Biometrics, Centre for Medical Statistics, Informatics and Intelligent Systems, Medical University of Vienna, Vienna, Austria; 8 Julius Center for Health Sciences and Primary Care, University Medical Centre Utrecht, Utrecht University, Utrecht, Netherlands; 9 Cochrane Netherlands, University Medical Centre Utrecht, Utrecht University, Utrecht, Netherlands; 10 Department of Medical Microbiology, University Medical Centre Utrecht, Utrecht, Netherlands; 11 Department of Electrical Engineering, ESAT Stadius, KU Leuven, Leuven, Belgium; 12 Ordensklinikum Linz, Hospital Elisabethinen, Department of Nephrology, Linz, Austria; 13 Department of Biostatistics, Epidemiology and Informatics, Perelman School of Medicine, University of Pennsylvania, Philadelphia, PA, USA; 14 Palliative and Advanced Illness Research (PAIR) Center and Division of Pulmonary and Critical Care Medicine, Department of Medicine, Perelman School of Medicine, University of Pennsylvania, Philadelphia, PA, USA; 15 Department of Microbiology, Immunology and Transplantation, KU Leuven-University of Leuven, Leuven, Belgium; 16 Department of General Internal Medicine, KU Leuven-University Hospitals Leuven, Leuven, Belgium; 17 Evidence-Based Oncology, Department I of Internal Medicine and Center for Integrated Oncology Aachen Bonn Cologne Dusseldorf, Faculty of Medicine and University Hospital Cologne, University of Cologne, Cologne, Germany; 18 Department of Clinical Epidemiology, Leiden University Medical Centre, Leiden, Netherlands; 19 Centre for Biostatistics, University of Manchester, Manchester Academic Health Science Centre, Manchester, UK; 20 Centre for Epidemiology Versus Arthritis, Centre for Musculoskeletal Research, University of Manchester, Manchester Academic Health Science Centre, Manchester, UK; 21 Division of Nursing, Midwifery and Social Work, School of Health Sciences, University of Manchester; 22 Faculty of Biology, Medicine and Health, University of Manchester, Manchester, UK; 23 Department of Clinical Epidemiology and Medical Technology Assessment, Maastricht University Medical Centre+, Maastricht, Netherlands; 24 Charité Universitätsmedizin Berlin, corporate member of Freie Universität Berlin, Humboldt-Universität zu Berlin, Berlin, Germany; 25 Berlin Institute of Health, Berlin, Germany

## Abstract

**Objective:**

To review and critically appraise published and preprint reports of prediction models for diagnosing coronavirus disease 2019 (covid-19) in patients with suspected infection, for prognosis of patients with covid-19, and for detecting people in the general population at increased risk of becoming infected with covid-19 or being admitted to hospital with the disease.

**Design:**

Living systematic review and critical appraisal.

**Data sources:**

PubMed and Embase through Ovid, Arxiv, medRxiv, and bioRxiv up to 7 April 2020.

**Study selection:**

Studies that developed or validated a multivariable covid-19 related prediction model.

**Data extraction:**

At least two authors independently extracted data using the CHARMS (critical appraisal and data extraction for systematic reviews of prediction modelling studies) checklist; risk of bias was assessed using PROBAST (prediction model risk of bias assessment tool).

**Results:**

4909 titles were screened, and 51 studies describing 66 prediction models were included. The review identified three models for predicting hospital admission from pneumonia and other events (as proxy outcomes for covid-19 pneumonia) in the general population; 47 diagnostic models for detecting covid-19 (34 were based on medical imaging); and 16 prognostic models for predicting mortality risk, progression to severe disease, or length of hospital stay. The most frequently reported predictors of presence of covid-19 included age, body temperature, signs and symptoms, sex, blood pressure, and creatinine. The most frequently reported predictors of severe prognosis in patients with covid-19 included age and features derived from computed tomography scans. C index estimates ranged from 0.73 to 0.81 in prediction models for the general population, from 0.65 to more than 0.99 in diagnostic models, and from 0.85 to 0.99 in prognostic models. All models were rated at high or unclear risk of bias, mostly because of non-representative selection of control patients, exclusion of patients who had not experienced the event of interest by the end of the study, high risk of model overfitting, and vague reporting. Most reports did not include any description of the study population or intended use of the models, and calibration of the model predictions was rarely assessed.

**Conclusion:**

Prediction models for covid-19 are quickly entering the academic literature to support medical decision making at a time when they are urgently needed. This review indicates that proposed models are poorly reported, at high risk of bias, and their reported performance is probably optimistic. Hence, we do not recommend any of these reported prediction models to be used in current practice. Immediate sharing of well documented individual participant data from covid-19 studies and collaboration are urgently needed to develop more rigorous prediction models, and validate promising ones. The predictors identified in included models should be considered as candidate predictors for new models. Methodological guidance should be followed because unreliable predictions could cause more harm than benefit in guiding clinical decisions. Finally, studies should adhere to the TRIPOD (transparent reporting of a multivariable prediction model for individual prognosis or diagnosis) reporting guideline.

**Systematic review registration:**

Protocol https://osf.io/ehc47/, registration https://osf.io/wy245.

**Readers’ note:**

This article is a living systematic review that will be updated to reflect emerging evidence. Updates may occur for up to two years from the date of original publication. This version is update 1 of the original article published on 7 April 2020 (*BMJ* 2020;369:m1328), and previous updates can be found as data supplements (https://www.bmj.com/content/369/bmj.m1328/related#datasupp).****

## Introduction

The novel coronavirus disease 2019 (covid-19) presents an important and urgent threat to global health. Since the outbreak in early December 2019 in the Hubei province of the People’s Republic of China, the number of patients confirmed to have the disease has exceeded 3 231 701 in more than 180 countries, and the number of people infected is probably much higher. More than 220 000 people have died from covid-19 (up to 30 April 2020).^1^ Despite public health responses aimed at containing the disease and delaying the spread, several countries have been confronted with a critical care crisis, and more countries could follow.^2^ ^3^ ^4^ Outbreaks lead to important increases in the demand for hospital beds and shortage of medical equipment, while medical staff themselves could also get infected.

To mitigate the burden on the healthcare system, while also providing the best possible care for patients, efficient diagnosis and information on the prognosis of the disease is needed. Prediction models that combine several variables or features to estimate the risk of people being infected or experiencing a poor outcome from the infection could assist medical staff in triaging patients when allocating limited healthcare resources. Models ranging from rule based scoring systems to advanced machine learning models (deep learning) have been proposed and published in response to a call to share relevant covid-19 research findings rapidly and openly to inform the public health response and help save lives.^5^ Many of these prediction models are published in open access repositories, ahead of peer review.

We aimed to systematically review and critically appraise all currently available prediction models for covid-19, in particular models to predict the risk of developing covid-19 or being admitted to hospital with covid-19, models to predict the presence of covid-19 in patients with suspected infection, and models to predict the prognosis or course of infection in patients with covid-19. We include model development and external validation studies. This living systematic review, with periodic updates, is being conducted in collaboration with the Cochrane Prognosis Methods Group.

## Methods

We searched PubMed and Embase through Ovid, bioRxiv, medRxiv, and arXiv for research on covid-19 published after 3 January 2020. We used the publicly available publication list of the covid-19 living systematic review.^6^ This list contains studies on covid-19 published on PubMed and Embase through Ovid, bioRxiv, and medRxiv, and is continuously updated. We validated the list to examine whether it is fit for purpose by comparing it to relevant hits from bioRxiv and medRxiv when combining covid-19 search terms (covid-19, sars-cov-2, novel corona, 2019-ncov) with methodological search terms (diagnostic, prognostic, prediction model, machine learning, artificial intelligence, algorithm, score, deep learning, regression). All relevant hits were found on the living systematic review list.^6^ We supplemented this list with hits from PubMed by searching for “covid-19” because when we performed our initial search this term was not included in the reported living systematic review^6^ search terms for PubMed. We further supplemented the list with studies on covid-19 retrieved from arXiv. The online supplementary material presents the search strings. Additionally, we contacted authors for studies that were not publicly available at the time of the search,^7^ ^8^ and included studies that were publicly available but not on the living systematic review^6^ list at the time of our search.^9^ ^10^ ^11^ ^12^

We searched databases on 13 March 2020 and 24 March 2020 (for the first version of the review), and 7 April 2020 (for the first update of the review). All studies were considered, regardless of language or publication status (preprint or peer reviewed articles; updates of preprints will only be included and reassessed in future updates after publication in a peer reviewed journal). We included studies if they developed or validated a multivariable model or scoring system, based on individual participant level data, to predict any covid-19 related outcome. These models included three types of prediction models: diagnostic models for predicting the presence of covid-19 in patients with suspected infection; prognostic models for predicting the course of infection in patients with covid-19; and prediction models to identify people at increased risk of developing covid-19 in the general population. No restrictions were made on the setting (eg, inpatients, outpatients, or general population), prediction horizon (how far ahead the model predicts), included predictors, or outcomes. Epidemiological studies that aimed to model disease transmission or fatality rates, diagnostic test accuracy, and predictor finding studies were excluded. Titles, abstracts, and full texts were screened in duplicate for eligibility by independent reviewers (two from LW, BVC, and MvS), and discrepancies were resolved through discussion.

Data extraction of included articles was done by two independent reviewers (from LW, BVC, GSC, TPAD, MCH, GH, KGMM, RDR, ES, LJMS, EWS, KIES, CW, AL, JM, TT, JAAD, KL, JBR, LH, CS, MS, MCH, NS, NK, SMJvK, JCS, PD, CLAN, and MvS). Reviewers used a standardised data extraction form based on the CHARMS (critical appraisal and data extraction for systematic reviews of prediction modelling studies) checklist^13^ and PROBAST (prediction model risk of bias assessment tool) for assessing the reported prediction models.^14^ We sought to extract each model’s predictive performance by using whatever measures were presented. These measures included any summaries of discrimination (the extent to which predicted risks discriminate between participants with and without the outcome), and calibration (the extent to which predicted risks correspond to observed risks) as recommended in the TRIPOD (transparent reporting of a multivariable prediction model for individual prognosis or diagnosis) statement.^15^ Discrimination is often quantified by the C index (C index=1 if the model discriminates perfectly; C index=0.5 if discrimination is no better than chance). Calibration is often quantified by the calibration intercept (which is zero when the risks are not systematically overestimated or underestimated) and calibration slope (which is one if the predicted risks are not too extreme or too moderate).^16^ We focused on performance statistics as estimated from the strongest available form of validation (in order of strength: external (evaluation in an independent database), internal (bootstrap validation, cross validation, random training test splits, temporal splits), apparent (evaluation by using exactly the same data used for development)). Any discrepancies in data extraction discussed between reviewers, followed by conflict resolution by LW and MvS if needed. The online supplementary material provides details on data extraction. We considered aspects of PRISMA (preferred reporting items for systematic reviews and meta-analyses)^17^ and TRIPOD^15^ in reporting our article. 

### Patient and public involvement

It was neither appropriate nor possible to involve patients or the public in the design, conduct, or reporting of our research. The study protocol and preliminary results are publicly available on https://osf.io/ehc47/ and medRxiv.

## Results

**Fig 1:**
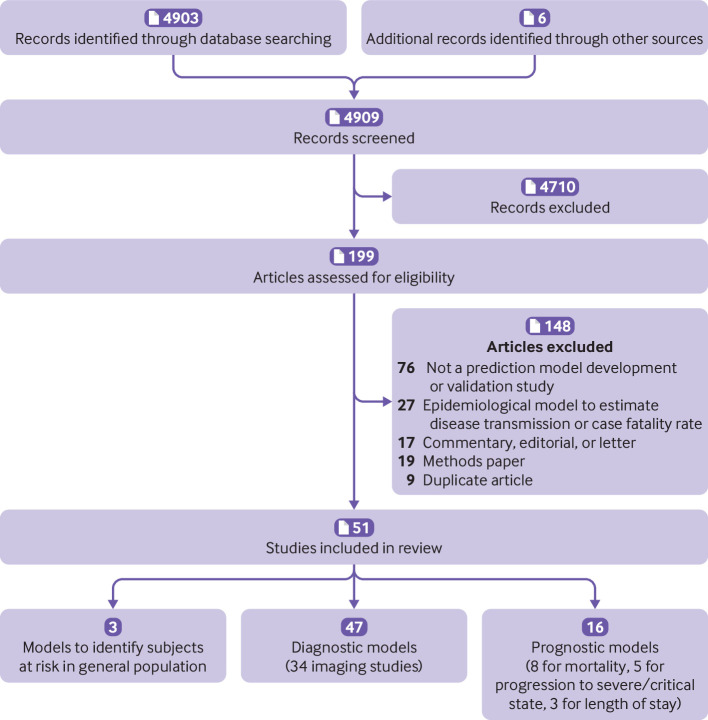
PRISMA (preferred reporting items for systematic reviews and meta-analyses) flowchart of study inclusions and exclusions. CT=computed tomography

We retrieved 4903 titles through our systematic search (fig 1; 1916 on 13 March 2020 and 774 on 24 March 2020, included in the first version of the review; and 2213 on 7 April 2020, included in the first update). Two additional unpublished studies were made available on request (after a call on social media). We included a further four studies that were publicly available but were not detected by our search. Of 4909 titles, 199 studies were retained for abstract and full text screening (85 in the first version of the review; 114 were added in the first update). Fifty one studies describing 66 prediction models met the inclusion criteria (31 models in 27 papers included in the first version of the review; 35 models in 24 papers added in the first update).^7^ ^8^ ^9^ ^10^ ^11^ ^12^ ^18^ ^19^ ^20^ ^21^ ^22^ ^23^ ^24^ ^25^ ^26^ ^27^ ^28^ ^29^ ^30^ ^31^ ^32^ ^33^ ^34^ ^35^ ^36^ ^37^ ^38^ ^39^ ^40^ ^41^ ^42^ ^43^ ^44^ ^45^ ^46^ ^47^ ^48^ ^49^ ^50^ ^51^ ^52^ ^53^ ^54^ ^55^ ^56^ ^57^ ^58^ ^59^ ^60^ ^61^ ^62^ These studies were selected for data extraction and critical appraisal (table 1 and table 2).

**Table 1:** Overview of prediction models for diagnosis and prognosis of covid-19

Study; setting; and outcome	Predictors in final model	Sample size: total No of participants for model development set (No with outcome)	Predictive performance on validation			Overall risk of bias using PROBAST
			Type of validation*	Sample size: total No of participants for model validation (No with outcome)	Performance* (C index, sensitivity (%), specificity (%), PPV/NPV (%), calibration slope, other (95% CI, if reported))	
**General population**						
Decaprio et al^8^; data from US general population; hospital admission for covid-19 pneumonia (proxy events)†	Age, sex, number of previous hospital admissions, 11 diagnostic features, interactions between age and diagnostic features	1.5 million (unknown)	Training test split	369 865 (unknown)	C index 0.73	High
Decaprio et al^8^; data from US general population; hospital admission for covid-19 pneumonia (proxy events)†	Age and ≥500 features related to diagnosis history	1.5 million (unknown)	Training test split	369 865 (unknown)	C index 0.81	High
Decaprio et al^8^; data from US general population; hospital admission for covid-19 pneumonia (proxy events)†	≥500 undisclosed features, including age, diagnostic history, social determinants of health, Charlson comorbidity index	1.5 million (unknown)	Training test split	369 865 (unknown)	C index 0.81	High
**Diagnosis**						
Original review						
Feng et al^10^; data from China, patients presenting at fever clinic; suspected covid-19 pneumonia	Age, temperature, heart rate, diastolic blood pressure, systolic blood pressure, basophil count, platelet count, mean corpuscular haemoglobin content, eosinophil count, monocyte count, fever, shiver, shortness of breath, headache, fatigue, sore throat, fever classification, interleukin 6	132 (26)	Temporal validation	32 (unclear)	C index 0.94	High
Lopez-Rincon et al^35^; data from international genome sequencing data repository, target population unclear; covid-19 diagnosis	Specific sequences of base pairs	553 (66)	10-fold cross validation	Not applicable	C index 0.98, sensitivity100, specificity 99	High
Meng et al^12^; data from China, asymptomatic patients with suspected covid-19; covid-19 diagnosis	Age, activated partial thromboplastin time, red blood cell distribution width SD, uric acid, triglyceride, serum potassium, albumin/globulin, 3-hydroxybutyrate, serum calcium	620 (302)	External validation	145 (80)	C index 0.87‡	High
Song et al^30^; data from China, inpatients with suspected covid-19; covid-19 diagnosis	Fever, history of close contact, signs of pneumonia on CT, neutrophil to lymphocyte ratio, highest body temperature, sex, age, meaningful respiratory syndromes	304 (73)	Training test split	95 (18)	C index 0.97 (0.93 to 1.00)	High
Yu et al^24^; data from China, paediatric inpatients with confirmed covid-19; severe disease (yes/no) defined based on clinical symptoms	Direct bilirubin; alanine transaminase	105 (8)	Apparent performance only	Not applicable	F1 score 1.00	High
Update 1						
Martin et al^41^; simulated patients with suspected covid-19; covid-19 diagnosis	Unknown	Not applicable	External validation only (simulation)	Not applicable	Sensitivity 97, specificity 96	High
Sun et al^40^; data from Singapore, patients with suspected infection presenting at infectious disease clinic; covid-19 diagnosis	Age, sex, temperature, heart rate, systolic blood pressure, diastolic blood pressure, sore throat	292 (49)	Leave-one-out cross validation	Not applicable	C index 0.65 (0.57 to 0.73)	High
Sun et al^40^; data from Singapore, patients with suspected infection presenting at infectious disease clinic; covid-19 diagnosis	Sex, temperature, heart rate, respiration rate, diastolic blood pressure, sore throat, sputum production, shortness of breath, gastrointestinal symptoms, lymphocytes, neutrophils, eosinophils, creatinine	292 (49)	Leave-one-out cross validation	Not applicable	C index 0.88 (0.83 to 0.93)	High
Sun et al^40^; data from Singapore, patients with suspected infection presenting at infectious disease clinic; covid-19 diagnosis	Sex, temperature, heart rate, respiration rate, diastolic blood pressure, sputum production, gastrointestinal symptoms, chest radiograph or CT scan suggestive of pneumonia, neutrophils, eosinophils, creatinine	292 (49)	Leave-one-out cross validation	Not applicable	C index 0.88 (0.83 to 0.93)	High
Sun et al^40^; data from Singapore, patients with suspected infection presenting at infectious disease clinic; covid-19 diagnosis	Sex, covid-19 case contact, travel to Wuhan, travel to China, temperature, heart rate, respiration rate, diastolic blood pressure, sore throat, sputum production, gastrointestinal symptoms, chest radiograph or CT scan suggestive of pneumonia, neutrophils, eosinophils, creatinine, sodium	292 (49)	Leave-one-out cross validation	Not applicable	C index 0.91 (0.86 to 0.96)	High
Wang et^43^; data from China, patients with suspected covid-19; covid-19 pneumonia	Epidemiological history, wedge shaped or fan shaped lesion parallel to or near the pleura, bilateral lower lobes, ground glass opacities, crazy paving pattern, white blood cell count	178 (69)	External validation	116 (68)	C index 0.85, calibration slope 0.56	High
Wu et al^45^; data from China, inpatients with suspected covid-19; covid-19 diagnosis	Lactate dehydrogenase, calcium, creatinine, total protein, total bilirubin, basophil, platelet distribution width, kalium, magnesium, creatinine kinase isoenzyme, glucose	108 (12)	Training test split	107 (61)	C index 0.99, sensitivity 100, specificity 94	High
Zhou et al^46^; data from China, inpatients with confirmed covid-19; severe pneumonia	Age, sex, onset-admission time, high blood pressure, diabetes, CHD, COPD, white blood cell counts, lymphocyte, neutrophils, alanine transaminase, aspartate aminotransferase, serum albumin, serum creatinine, blood urea nitrogen, CRP	250 (79)	Training test split	127 (38)	C index 0.88 (0.94 to 0.92), sensitivity 89, specificity 74	High
**Diagnostic imaging**						
Original review						
Barstugan et al^31^; data from Italy, patients with suspected covid-19; covid-19 diagnosis	Not applicable	53 (not applicable)	Cross validation	Not applicable	Sensitivity 93, specificity 100	High
Chen et al^26^; data from China, people with suspected covid-19 pneumonia; covid-19 pneumonia	Not applicable	106 (51)	Training test split	27 (11)	Sensitivity 100, specificity 82	High
Gozes et al^25^; data from China and US,§ patients with suspected covid-19; covid-19 diagnosis	Not applicable	50 (unknown)	External validation with Chinese cases and US controls	Unclear	C index 0.996 (0.989 to 1.000)	High
Jin et al^11^; data from China, US, and Switzerland,¶ patients with suspected covid-19; covid-19 diagnosis	Not applicable	416 (196)	Training test split	1255 (183)	C index 0.98, sensitivity 94, specificity 95	High
Jin et al^33^; data from China, patients with suspected covid-19; covid-19 pneumonia	Not applicable	1136 (723)	Training test split	282 (154)	C index: 0.99, sensitivity 97, specificity 92	High
Li et al^34^; data from China, patients with suspected covid-19; covid-19 diagnosis	Not applicable	2969 (400)	Training test split	353 (68)	C index 0.96 (0.94 to 0.99), sensitivity 90 (83 to 94), specificity 96 (93 to 98)	High
Shan et al^28^; data from China, people with confirmed covid-19; segmentation and quantification of infection regions in lung from chest CT scans	Not applicable	249 (not applicable)	Training test split	300 (not applicable)	Dice similarity coefficient 91.6%**	High
Shi et al^36^; data from China, target population unclear; covid-19 pneumonia	5 categories of location features from imaging: volume, number, histogram, surface, radiomics	2685 (1658)	Fivefold cross validation	Not applicable	C index 0.94	High
Wang et al^29^; data from China, target population unclear; covid-19 diagnosis	Not applicable	259 (79)	Internal, other images from same people	Not applicable	C index 0.81 (0.71 to 0.84), sensitivity 83, specificity 67	High
Xu et al^27^; data from China, target population unclear; covid-19 diagnosis	Not applicable	509 (110)	Training test split	90 (30)	Sensitivity 87, PPV 81	High
Song et al^23^; data from China, target population unclear; diagnosis of covid-19 *v* healthy controls	Not applicable	123 (61)	Training test split	51 (27)	C index 0.99	High
Song et al^23^; data from China, target population unclear; diagnosis of covid-19 *v* bacterial pneumonia	Not applicable	131 (61)	Training test split	57 (27)	C index 0.96	High
Zheng et al^38^; data from China, target population unclear; covid-19 diagnosis	Not applicable	Unknown	Temporal validation	Unknown	C index 0.96	High
Update 1						
Abbas et al^47^; data from repositories (origin unspecified), target population unclear; covid-19 diagnosis	Not applicable	137 (unknown)	Training test split	59 (unknown)	C index 0.94, sensitivity 98, specificity 92	High
Apostolopoulos et al^48^; data from repositories (US, Italy); patients with suspected covid-19; covid-19 diagnosis	Not applicable	1427 (224)	10-fold cross validation	Not applicable	Sensitivity 99, specificity 97	High
Bukhari et al^49^; data from Canada and US; patients with suspected covid-19; covid-19 diagnosis	Not applicable	223 (unknown)	Training test split	61 (17)	Sensitivity 98, PPV 91	High
Chaganti et al^50^; data from Canada, US, and European countries; patients with suspected covid-19; percentage lung opacity	Not applicable	631 (not applicable)	Training test split	100 (not applicable)	Correlation§§ 0.98	High
Chaganti et al^50^; data from Canada, US, and European countries; patients with suspected covid-19; percentage high lung opacity	Not applicable	631 (not applicable)	Training test split	100 (not applicable)	Correlation§§ 0.98	High
Chaganti et al^50^; data from Canada, US, and European countries; patients with suspected covid-19; severity score	Not applicable	631 (not applicable)	Training test split	100 (not applicable)	Correlation§§ 0.97	High
Chaganti et al^50^; data from Canada, US, and European countries; patients with suspected covid-19; lung opacity score	Not applicable	631 (not applicable)	Training test split	100 (not applicable)	Correlation§§ 0.97	High
Chowdhury et al^39^; data from repositories (Italy and other unspecified countries), target population unclear; covid-19 *v* “normal”	Not applicable	Unknown	Fivefold cross validation	Not applicable	C index 0.99	High
Chowdhury et al^39^; data from repositories (Italy and other unspecified countries), target population unclear; covid-19 *v* “normal” and viral pneumonia	Not applicable	Unknown	Fivefold cross validation	Not applicable	C index 0.98	High
Chowdhury et al^39^; data from repositories (Italy and other unspecified countries), target population unclear; covid-19 *v* “normal”	Not applicable	Unknown	Fivefold cross validation	Not applicable	C index 0.998	High
Chowdhury et al^39^; data from repositories (Italy and other unspecified countries), target population unclear; covid-19 *v* “normal” and viral pneumonia	Not applicable	Unknown	Fivefold cross validation	Not applicable	C index 0.99	High
Fu et al^51^; data from China, target population unclear; covid-19 diagnosis	Not applicable	610 (100)	External validation	309 (50)	C index 0.99, sensitivity 97, specificity 99	High
Gozes et al^52^; data from China, people with suspected covid-19; covid-19 diagnosis	Not applicable	50 (unknown)	External validation	199 (109)	C index 0.95 (0.91 to 0.99)	High
Imran et al^53^; data from unspecified source, target population unclear; covid-19 diagnosis	Not applicable	357 (48)	Twofold cross validation	Not applicable	Sensitivity 90, specificity 81	High
Li et al^54^; data from China, inpatients with confirmed covid-19; severe and critical covid-19	Severity score based on CT scans	Not applicable	External validation of existing score	78 (not applicable)	C index 0.92 (0.84 to 0.99)	High
Li et al^55^; data from unknown origin, patients with suspected covid-19; covid-19	Not applicable	360 (120)	Training test split	135 (45)	C index 0.97	High
Hassanien et al^56^; data from repositories (origin unspecified), people with suspected covid-19; covid-19 diagnosis	Not applicable	Unknown	Training test split	Unknown	Sensitivity 95, specificity 100	High
Tang et al^57^; data from China, patients with confirmed covid-19; covid-19 severe *v* non-severe	Not applicable	176 (55)	Threefold cross validation	Not applicable	C index 0.91, sensitivity 93, specificity 75	High
Wang et al^42^; data from China, inpatients with suspected covid-19; covid-19	Not applicable	709 (560)	External validation in other centres	508 (223)	C index (average) 0.87	High
Zhang et al^58^; data from repositories (origin unspecified), people with suspected covid-19; covid-19	Not applicable	1078 (70)	Twofold cross validation	Not applicable	C index 0.95, sensitivity 96, specificity 71	High
Zhou et al^59^; data from China, patients with suspected covid-19; covid-19 diagnosis	Not applicable	191 (35)	External validation in other centres	107 (57)	C index 0.92, sensitivity 83, specificity 86	High
**Prognosis**						
Original review						
Bai et al^9^; data from China, inpatients at admission with mild confirmed covid-19; deterioration into severe/critical disease (period unspecified)	Combination of demographics, signs and symptoms, laboratory results and features derived from CT images	133 (54)	Unclear	Not applicable	C index 0.95 (0.94 to 0.97)	High
Caramelo et al^18^; data from China, target population unclear; mortality (period unspecified)††	Age, sex, presence of any comorbidity (hypertension, diabetes, cardiovascular disease, chronic respiratory disease, cancer)††	Unknown	Not reported	Not applicable	Not reported	High
Gong et al^32^; data from China, inpatients with confirmed covid-19 at admission; severe covid-19 (within minimum 15 days)	Age, serum LDH, CRP, variation of red blood cell distribution width, blood urea nitrogen, albumin, direct bilirubin	189 (28)	External validation (two centres)	165 (40) and 18 (4)	Centre 1: C index 0.85 (0.79 to 0.92), sensitivity 78, specificity 78; centre 2: sensitivity 75, specificity 100	High
Lu et al^19^; data from China, inpatients at admission with suspected or confirmed covid-19; mortality (within 12 days)	Age, CRP	577 (44)	Not reported	Not applicable	Not reported	High
Qi et al^20^; data from China, inpatients with confirmed covid-19 at admission; hospital stay >10 days	6 features derived from CT images‡‡ (logistic regression model)	26 (20)	5 fold cross validation	Not applicable	C index 0.92	High
Qi et al^20^; data from China, inpatients with confirmed covid-19 at admission; hospital stay >10 days	6 features derived from CT images‡‡ (random forest)	26 (20)	5 fold cross validation	Not applicable	C index 0.96	High
Shi et al^37^; data from China, inpatients with confirmed covid-19 at admission; death or severe covid-19 (period unspecified)	Age (dichotomised), sex, hypertension	478 (49)	Validation in less severe cases	66 (15)	Not reported	High
Xie et al^7^; data from China, inpatients with confirmed covid-19 at admission; mortality (in hospital)	Age, LDH, lymphocyte count, SPO_2_	299 (155)	External validation (other Chinese centre)	130 (69)	C index 0.98 (0.96 to 1.00), calibration slope 2.5 (1.7 to 3.7)	High
Yan et al^21^; data from China, inpatients suspected of covid-19; mortality (period unspecified)	LDH, lymphocyte count, high sensitivity CRP	375 (174)	Temporal validation, selecting only severe cases	29 (17)	Sensitivity 92, PPV 95	High
Yuan et al^22^; data from China, inpatients with confirmed covid-19 at admission; mortality (period unspecified)	Clinical scorings of CT images (zone, left/right, location, attenuation, distribution of affected parenchyma)	Not applicable	External validation of existing model	27 (10)	C index 0.90 (0.87 to 0.93)	High
Update 1						
Huang et al^60^; data from China, inpatients with confirmed covid-19 at admission; severe symptoms three days after admission	Underlying diseases, fast respiratory rate >24/min, elevated CRP level (>10 mg/dL), elevated LDH level (>250 U/L)	125 (32)	Apparent performance only	Not applicable	C index 0.99 (0.97 to 1.00), sensitivity 0.91, specificity 0.96	High
Pourhomayoun et al^61^; data from 76 countries, inpatients with confirmed covid-19; in-hospital mortality (period unspecified)	Unknown	Unknown	10-fold cross validation	Not applicable	C index 0.96, sensitivity 90, specificity 0.97	High
Sarkar et al^44^; data from several continents (Australia, Asia, Europe, North America), inpatients with covid-19 symptoms; death *v* recovery (period unspecified)	Age, days from symptom onset to hospitalisation, from Wuhan, sex, visit to Wuhan	80 (37)	Apparent performance only	Not applicable	C index 0.97	High
Wang et al^42^; data from China, inpatients with confirmed covid-19; length of hospital stay	Age and CT features	301 (not applicable)	Not reported	Not applicable	Not reported	High
Zeng et al^62^; data from China, inpatients with confirmed covid-19; severe disease progression (period unspecified)	CT features	338 (76)	Cross validation (number of folds unclear)	Not applicable	C index 0.88	High
Zeng et al^62^; data from China, inpatients with confirmed covid-19; severe disease progression (period unspecified)	CT features and laboratory markers	338 (76)	Cross validation (number of folds unclear)	Not applicable	C index 0.88	High

CHD=coronary heart disease; COPD=chronic obstructive pulmonary disease; covid-19=coronavirus disease 2019; CRP=C reactive protein; CT=computed tomography; LDH=lactate dehydrogenase; NPV=negative predictive value; PPV=positive predictive value; PROBAST=prediction model risk of bias assessment tool; SPO_2_=oxygen saturation.

* Performance is given for the strongest form of validation reported. This is indicated in the column “type of validation.” When a training test split was used, performance on the test set is reported. Apparent performance is the performance observed in the development data.

† Proxy events used: pneumonia (except from tuberculosis), influenza, acute bronchitis, or other specified upper respiratory tract infections (no patients with covid-19 pneumonia in data).

‡ Calibration plot presented, but unclear which data were used.

§ The development set contains scans from Chinese patients, the testing set contains scans from Chinese cases and controls, and US controls.

¶ Data contain mixed cases and controls. Chinese data and controls from US and Switzerland.

** Describes similarity between segmentation of the CT scan by a medical doctor and automated segmentation.

†† Outcome and predictor data were simulated.

‡‡ Wavelet-HLH_gldm_SmallDependenceLowGrayLevelEmphasis, wavelet-LHH_glcm_Correlation, wavelet-LHL_glszm_GrayLevelVariance, wavelet-LLH_glszm_SizeZoneNonUniformityNormalized, wavelet-LLH_glszm_SmallAreaEmphasis, wavelet-LLH_glcm_Correlation. §§Pearson correlation between the predicted and ground truth scores for patients with lung abnormalities.

**Table 2:** Risk of bias assessment (using PROBAST) based on four domains across 51 studies that created prediction models for coronavirus disease 2019

Authors	Risk of bias			
	Participants	Predictors	Outcome	Analysis
**Hospital admission in general population**				
DeCaprio et al^8^	High	Low	High	High
**Diagnosis**				
Original review				
Feng et al^10^	Low	Unclear	High	High
Lopez-Rincon et al^35^	Unclear	Low	Low	High
Meng et al^12^	High	Low	High	High
Song et al^30^	High	Unclear	Low	High
Yu et al^24^	Unclear	Unclear	Unclear	High
Update 1				
Martin et al^41^	High	High	High	High
Sun et al^40^	Low	Low	Unclear	High
Wang et al^43^	Low	Unclear	Unclear	High
Wu et al^45^	High	Unclear	Low	High
Zhou et al^46^	Unclear	Low	High	High
**Diagnostic imaging**				
Original review				
Barstugan et al^31^	Unclear	Unclear	Unclear	High
Chen et al^26^	High	Unclear	Low	High*
Gozes et al^25^	Unclear	Unclear	High	High
Jin et al^11^	High	Unclear	Unclear	High†
Jin et al^33^	High	Unclear	High	High*
Li et al^34^	Low	Unclear	Low	High
Shan et al^28^	Unclear	Unclear	High	High†
Shi et al^36^	High	Unclear	Low	High
Wang et al^29^	High	Unclear	Low	High
Xu et al^27^	High	Unclear	High	High
Song et al^23^	Unclear	Unclear	Low	High
Zheng et al^38^	Unclear	Unclear	High	High
Update 1				
Abbas et al^47^	High	Unclear	Unclear	High
Apostolopoulos et al^48^	High	Unclear	High	High
Bukhari et al^49^	Unclear	Unclear	Unclear	High
Chaganti et al^50^	High	Unclear	Low	Unclear
Chowdhury et al^39^	High	Unclear	Unclear	High
Fu et al^51^	High	Unclear	Unclear	High
Gozes et al^52^	High	Unclear	Unclear	High
Imran et al^53^	High	Unclear	Unclear	High*
Li et al^54^	Low	Low	Unclear	High
Li et al^55^	High	Unclear	High	High*
Hassanien et al^56^	Unclear	Unclear	Unclear	High*
Tang et al^57^	Unclear	Unclear	High	High
Wang et al^42^	Low	Unclear	Unclear	High
Zhang et al^58^	High	Unclear	High	High
Zhou et al^59^	High	Unclear	High	High*
**Prognosis**				
Original review				
Bai et al^9^	Low	Unclear	Unclear	High
Caramelo et al^18^	High	High	High	High
Gong et al^32^	Low	Unclear	Unclear	High
Lu et al^19^	Low	Low	Low	High
Qi et al^20^	Unclear	Low	Low	High
Shi et al^37^	High	High	High	High
Xie et al^7^	Low	Low	Low	High
Yan et al^21^	Low	High	Low	High
Yuan et al^22^	Low	High	Low	High
Update 1				
Huang et al^60^	Unclear	Unclear	Unclear	High
Pourhomayoun et al^61^	Low	Low	Unclear	High
Sarkar et al^44^	High	High	High	High
Wang et al^42^	Low	Low	Low	High
Zeng et al^62^	Low	Low	Low	High

PROBAST=prediction model risk of bias assessment tool.

* Risk of bias high owing to calibration not being evaluated. If this criterion is not taken into account, analysis risk of bias would have been unclear.

† Risk of bias high owing to calibration not being evaluated. If this criterion is not taken into account, analysis risk of bias would have been low.

### Primary datasets

Thirty two studies used data on patients with covid-19 from China, two studies used data on patients from Italy,^31^ ^39^ and one study used data on patients from Singapore^40^ (supplementary table 1). Ten studies used international data (supplementary table 1) and two studies used simulated data.^35^ ^41^ One study used US Medicare claims data from 2015 to 2016 to estimate vulnerability to covid-19.^8^ Three studies were not clear on the origin of covid-19 data (supplementary table 1).

Based on 26 of the 51 studies that reported study dates, data were collected between 8 December 2019 and 15 March 2020. The duration of follow-up was unclear in most studies. Two studies reported median follow-up time (8.4 and 15 days),^19^ ^37^ while another study reported a follow-up of at least five days.^42^ Some centres provided data to multiple studies and several studies used open Github^63^ or Kaggle^64^ data repositories (version or date of access often unclear), and so it was unclear how much these datasets overlapped across our 51 identified studies (supplementary table 1). One study^24^ developed prediction models for use in paediatric patients. The median age in studies on adults varied (from 34 to 65 years; see supplementary table 1), as did the proportion of men (from 41% to 67%), although this information was often not reported at all.

Among the six studies that developed prognostic models to predict mortality risk in people with confirmed or suspected infection, the percentage of deaths varied between 8% and 59% (table 1). This wide variation is partly because of severe sampling bias caused by studies excluding participants who still had the disease at the end of the study period (that is, they had neither recovered nor died).^7^ ^20^ ^21^ ^22^ ^44^ Additionally, length of follow-up could have varied between studies (but was rarely reported), and there might be local and temporal variation in how people were diagnosed as having covid-19 or were admitted to the hospital (and therefore recruited for the studies). Among the diagnostic model studies, only five reported on prevalence of covid-19 and used a cross sectional or cohort design; the prevalence varied between 17% and 79% (see table 1). Because 31 diagnostic studies used either case-control sampling or an unclear method of data collection, the prevalence in these diagnostic studies might not have been representative of their target population. 

Table 1 gives an overview of the 66 prediction models reported in the 51 identified studies. Supplementary table 2 provides modelling details and box 1 discusses the availability of models in a format for use in clinical practice.

Box 1 **Availability of models in format for use in clinical practice**

Sixteen studies presented their models in a format for use in clinical practice. However, because all models were at high risk of bias, we do not recommend their routine use before they are properly externally validated.

**Models to predict risk of developing coronavirus disease 2019 (covid-19) or of hospital admission for covid-19 in general population**

The “COVID-19 Vulnerability Index” to detect hospital admission for covid-19 pneumonia from other respiratory infections (eg, pneumonia, influenza) is available as an online tool.^8^ ^65^

**Diagnostic models**

The “COVID-19 diagnosis aid APP” is available on iOS and android devices to diagnose covid-19 in asymptomatic patients and those with suspected disease.^12^ The “suspected COVID-19 pneumonia Diagnosis Aid System” is available as an online tool.^10^ ^66^ The “COVID-19 early warning score” to detect covid-19 in adults is available as a score chart in an article.^30^ A nomogram (a graphical aid to calculate risk) is available to diagnose covid-19 pneumonia based on imaging features, epidemiological history, and white blood cell count.^43^ A decision tree to detect severe disease for paediatric patients with confirmed covid-19 is also available in an article.^24^ Additionally an online tool is available for diagnosis based on routine blood examination data.^45^

**Diagnostic models based on images**

Three artificial intelligence models to assist with diagnosis based on medical images are available through web applications.^23^ ^26^ ^29^ ^67^ ^68^ ^69^ One model is deployed in 16 hospitals, but the authors do not provide any usable tools in their study.^33^ One paper includes a “total severity score” to classify patients based on images.^54^

**Prognostic models**

To assist in the prognosis of mortality, a nomogram,^7^ a decision tree,^21^ and a computed tomography based scoring rule are available in the articles.^22^ Additionally a nomogram exists to predict progression to severe covid-19.^32^ A model equation to predict disease progression was made available in one paper.^60^

Overall, seven studies made their source code available on GitHub.^8^ ^11^ ^34^ ^35^ ^38^ ^47^ ^55^ Thirty one studies did not include any usable equation, format, or reference for use or validation of their prediction model.

### Models to predict risk of developing covid-19 or of hospital admission for covid-19 in general population

We identified three models that predicted risk of hospital admission for covid-19 pneumonia in the general population, but used admission for non-tuberculosis pneumonia, influenza, acute bronchitis, or upper respiratory tract infections as outcomes in a dataset without any patients with covid-19 (table 1).^8^ Among the predictors were age, sex, previous hospital admissions, comorbidity data, and social determinants of health. The study estimated C indices of 0.73, 0.81, and 0.81 for the three models.

### Diagnostic models to detect covid-19 in patients with suspected infection

Nine studies developed 13 multivariable models to diagnose covid-19. Most models target patients with suspected covid-19. Reported C index values ranged between 0.85 and 0.99, except for one model with a C index of 0.65. Two studies aimed to diagnose severe disease in patients with confirmed covid-19: one in adults with confirmed covid-19 with a reported C index value of 0.88,^46^ and one in paediatric patients with reported perfect performance.^24^ Several diagnostic predictors were used in more than one model: age (five models); body temperature or fever (three models); signs and symptoms (such as shortness of breath, headache, shiver, sore throat, and fatigue; three models); sex (three models); blood pressure (three models); creatinine (three models); epidemiological contact history, pneumonia signs on computed tomography scan, basophils, neutrophils, lymphocytes, alanine transaminase, albumin, platelets, eosinophils, calcium, and bilirubin (each in two models; table 1).

Thirty four prediction models were proposed to support the diagnosis of covid-19 or covid-19 pneumonia (and monitor progression) based on images. Most studies used computed tomography images. Other image sources were chest radiographs^39^ ^47^ ^48^ ^49^ ^55^ ^56^ ^58^ and spectrograms of cough sounds.^53^ The predictive performance varied widely, with estimated C index values ranging from 0.81 to 0.998.

### Prognostic models for patients with diagnosis of covid-19

We identified 16 prognostic models (table 1) for patients with a diagnosis of covid-19. Of these models, eight estimated mortality risk in patients with suspected or confirmed covid-19 (table 1). The intended use of these models (that is, when to use them, in whom to use them, and the prediction horizon, eg, mortality by what time) was not clearly described. Five models aimed to predict progression to a severe or critical state, and three aimed to predict length of hospital stay (table 1). Predictors (for any outcome) included age (seven models), features derived from computed tomography scoring (seven models), lactate dehydrogenase (four models), sex (three models), C reactive protein (three models), comorbidity (including hypertension, diabetes, cardiovascular disease, respiratory disease; three models), and lymphocyte count (three models; table 1).

Four studies that predicted mortality reported a C index between 0.90 and 0.98. One study also evaluated calibration.^7^ When applied to new patients, their model yielded probabilities of mortality that were too high for low risk patients and too low for high risk patients (calibration slope >1), despite excellent discrimination.^7^ One study developed two models to predict a hospital stay of more than 10 days and estimated C indices of 0.92 and 0.96.^20^ The other study predicting length of hospital stay did not report a C index. Neither study predicting length of hospital stay reported calibration. The five studies that developed models to predict progression to a severe or critical state reported C indices between 0.85 and 0.99. One of these studies also reported perfect calibration, but it was unclear how this was evaluated.^32^

### Risk of bias

All models were at high risk of bias according to assessment with PROBAST (table 1), which suggests that their predictive performance when used in practice is probably lower than that reported. Therefore, we have cause for concern that the predictions of these models are unreliable when used in other people. Box 2 gives details on common causes for risk of bias for each type of model.

Box 2 **Common causes of risk of bias in the reported prediction models**

**Models to predict risk of developing coronavirus disease 2019 (covid-19) or of hospital admission for covid-19 in general population**

These models were based on Medicare claims data, and used proxy outcomes to predict hospital admission for covid-19 pneumonia, in the absence of patients with covid-19.^8^

**Diagnostic models**

Controls are probably not representative of the target population for a diagnostic model (eg, controls for a screening model had viral pneumonia).^12^ ^41^ ^45^ The test used to determine the outcome varied between participants,^12^ ^41^ or one of the predictors (eg, fever) was part of the outcome definition.^10^

**Diagnostic models based on medical imaging**

Generally, studies did not clearly report which patients had imaging during clinical routine, and it was unclear whether the selection of controls was made from the target population (that is, patients with suspected covid-19). Often studies did not clearly report how regions of interest were annotated. Images were sometimes annotated by only one scorer without quality control.^25^ ^27^ ^47^ ^52^ ^55^ Careful description of model specification and subsequent estimation were lacking, challenging the transparency and reproducibility of the models. Every study used a different deep learning architecture, some were established and others specifically designed, without benchmarking the used architecture against others.

**Prognostic models**

Study participants were often excluded because they did not develop the outcome at the end of the study period but were still in follow-up (that is, they were in hospital but had not recovered or died), yielding a highly selected study sample.^7^ ^20^ ^21^ ^22^ ^44^ Additionally, only three studies accounted for censoring by using Cox regression^19^ ^42^ or competing risk models.^62^ One study used the last available predictor measurement from electronic health records (rather than measuring the predictor value at the time when the model was intended for use).^21^

Twenty four of the 51 studies had a high risk of bias for the participants domain (table 2), which indicates that the participants enrolled in the studies might not be representative of the models’ targeted populations. Unclear reporting on the inclusion of participants prohibited a risk of bias assessment in 13 studies. Six of the 51 studies had a high risk of bias for the predictor domain, which indicates that predictors were not available at the models’ intended time of use, not clearly defined, or influenced by the outcome measurement. The diagnostic model studies that used medical images as predictors in artificial intelligence were all scored as unclear on the predictor domain. One diagnostic imaging study used a simple scoring rule and was scored at low predictor risk of bias. The publications often lacked clear information on the preprocessing steps (eg, cropping of images). Moreover, complex machine learning algorithms transform images into predictors in a complex way, which makes it challenging to fully apply the PROBAST predictors section for such imaging studies. Most studies used outcomes that are easy to assess (eg, death, presence of covid-19 by laboratory confirmation). Nonetheless, there was reason to be concerned about bias induced by the outcome measurement in 18 studies, among others, because of the use of subjective or proxy outcomes (non covid-19 severe respiratory infections).

All but one study were at high risk of bias for the analysis domain (table 2). Many studies had small sample sizes (table 1), which led to an increased risk of overfitting, particularly if complex modelling strategies were used. Three studies did not report the predictive performance of the developed model, and three studies reported only the apparent performance (the performance with exactly the same data used to develop the model, without adjustment for optimism owing to potential overfitting). Only five studies assessed calibration,^7^ ^12^ ^32^ ^43^ ^50^ but the method to check calibration was probably suboptimal in two studies.^12^ ^32^

Nine models were developed and externally validated in the same study (in an independent dataset, excluding random training test splits and temporal splits).^7^ ^12^ ^25^ ^32^ ^42^ ^43^ ^51^ ^52^ ^59^ However, in six of these models, the datasets used for the external validation were not representative of the target population.^7^ ^12^ ^25^ ^42^ ^59^ Consequently, predictive performance could differ if the models are applied in the targeted population. In one study, commonly used performance statistics for prognosis (discrimination, calibration) were not reported.^42^ Gozes and colleagues^52^ and Fu and colleagues^51^ had satisfactory predictive performance on an external validation set, but it is unclear how the data for the external validation were collected, and whether they are representative. Gong and colleagues^32^ and Wang and colleagues^43^ obtained satisfactory discrimination on probably unbiased but small external validation datasets.

One study presented a small external validation (27 participants) that reported satisfactory predictive performance of a model originally developed for avian influenza H7N9 pneumonia. However, patients who had not recovered at the end of the study period were excluded, which again led to a selection bias.^22^ Another study was a small scale external validation study (78 participants) of an existing severity score for lung computed tomography images with satisfactory reported discrimination.^54^

## Discussion

In this systematic review of all prediction models related to the covid-19 pandemic, we identified and critically appraised 51 studies that described 66 models. These prediction models can be divided into three categories: models for the general population to predict the risk of developing covid-19 or being admitted to hospital for covid-19; models to support the diagnosis of covid-19 in patients with suspected infection; and models to support the prognostication of patients with covid-19. All models reported good to excellent predictive performance, but all were appraised to have high risk of bias owing to a combination of poor reporting and poor methodological conduct for participant selection, predictor description, and statistical methods used. As expected, in these early covid-19 related prediction model studies, clinical data from patients with covid-19 are still scarce and limited to data from China, Italy, and international registries. With few exceptions, the available sample sizes and number of events for the outcomes of interest were limited. This is a well known problem when building prediction models and increases the risk of overfitting the model.^70^ A high risk of bias implies that the performance of these models in new samples will probably be worse than that reported by the researchers. Therefore, the estimated C indices, often close to 1 and indicating near perfect discrimination, are probably optimistic. Eleven studies carried out an external validation,^7^ ^12^ ^22^ ^25^ ^32^ ^42^ ^43^ ^51^ ^52^ ^54^ ^59^ and calibration was rarely assessed. 

We reviewed 33 studies that used advanced machine learning methodology on medical images to diagnose covid-19, covid-19 related pneumonia, or to assist in segmentation of lung images. The predictive performance measures showed a high to almost perfect ability to identify covid-19, although these models and their evaluations also had a high risk of bias, notably because of poor reporting and an artificial mix of patients with and without covid-19. Therefore, we do not recommend any of the 66 identified prediction models to be used in practice. 

### Challenges and opportunities

The main aim of prediction models is to support medical decision making. Therefore it is vital to identify a target population in which predictions serve a clinical need, and a representative dataset (preferably comprising consecutive patients) on which the prediction model can be developed and validated. This target population must also be carefully described so that the performance of the developed or validated model can be appraised in context, and users know which people the model applies to when making predictions. Unfortunately, the included studies in our systematic review often lacked an adequate description of the study population, which leaves users of these models in doubt about the models’ applicability. Although we recognise that all studies were done under severe time constraints caused by urgency, we recommend that any studies currently in preprint and all future studies should adhere to the TRIPOD reporting guideline^15^ to improve the description of their study population and their modelling choices. TRIPOD translations (eg, in Chinese and Japanese) are also available at https://www.tripod-statement.org.

A better description of the study population could also help us understand the observed variability in the reported outcomes across studies, such as covid-19 related mortality. The variability in the relative frequencies of the predicted outcomes presents an important challenge to the prediction modeller. A prediction model applied in a setting with a different relative frequency of the outcome might produce predictions that are miscalibrated^71^ and might need to be updated before it can safely be applied in that new setting.^16^ Such an update might often be required when prediction models are transported to different healthcare systems, which requires data from patients with covid-19 to be available from that system.

Covid-19 prediction problems will often not present as a simple binary classification task. Complexities in the data should be handled appropriately. For example, a prediction horizon should be specified for prognostic outcomes (eg, 30 day mortality). If study participants have neither recovered nor died within that time period, their data should not be excluded from analysis, which most reviewed studies have done. Instead, an appropriate time to event analysis should be considered to allow for administrative censoring.^16^ Censoring for other reasons, for instance because of quick recovery and loss to follow-up of patients who are no longer at risk of death from covid-19, could necessitate analysis in a competing risk framework.^72^

Instead of developing and updating predictions in their local setting, individual participant data from multiple countries and healthcare systems might allow better understanding of the generalisability and implementation of prediction models across different settings and populations. This approach could greatly improve the applicability and robustness of prediction models in routine care.^73^ ^74^ ^75^ ^76^ ^77^

The evidence base for the development and validation of prediction models related to covid-19 will quickly increase over the coming months. Together with the increasing evidence from predictor finding studies^78^ ^79^ ^80^ ^81^ ^82^ ^83^ ^84^ and open peer review initiatives for covid-19 related publications,^85^ data registries^63^ ^64^ ^86^ ^87^ ^88^ are being set up. To maximise the new opportunities and to facilitate individual participant data meta-analyses, the World Health Organization has recently released a new data platform to encourage sharing of anonymised covid-19 clinical data.^89^ To leverage the full potential of these evolutions, international and interdisciplinary collaboration in terms of data acquisition and model building is crucial.

### Study limitations

With new publications on covid-19 related prediction models rapidly entering the medical literature, this systematic review cannot be viewed as an up-to-date list of all currently available covid-19 related prediction models. Also, 45 of the studies we reviewed were only available as preprints. These studies might improve after peer review, when they enter the official medical literature; we will reassess these peer reviewed publications in future updates. We also found other prediction models that are currently being used in clinical practice but without scientific publications,^90^ and web risk calculators launched for use while the scientific manuscript is still under review.^91^ These unpublished models naturally fall outside the scope of this review of the literature.

### Implications for practice

All 66 reviewed prediction models were found to have a high risk of bias, and evidence from independent external validation of the newly developed models is currently lacking. However, the urgency of diagnostic and prognostic models to assist in quick and efficient triage of patients in the covid-19 pandemic might encourage clinicians to implement prediction models without sufficient documentation and validation. Although we cannot let perfect be the enemy of good, earlier studies have shown that models were of limited use in the context of a pandemic,^92^ and they could even cause more harm than good.^93^ Therefore, we cannot recommend any model for use in practice at this point.

We anticipate that more covid-19 data at the individual participant level will soon become available. These data could be used to validate and update currently available prediction models.^16^ For example, one model predicted progression to severe covid-19 within 15 days of admission to hospital with promising discrimination when validated externally on two small but unselected cohorts.^32^ A second model to diagnose covid-19 pneumonia showed promising discrimination at external validation.^43^ A third model that used computed tomography based total severity scores showed good discrimination between patients with mild, common, and severe-critical disease.^54^ Because reporting in these studies was insufficiently detailed and the validation was in small Chinese datasets, validation in larger, international datasets is needed. Owing to differences between healthcare systems (eg, Chinese and European) on when patients are admitted to and discharged from hospital, and testing criteria for patients with covid-19, we anticipate most existing models will need to be updated (that is, adjusted to the local setting).

When creating a new prediction model, we recommend building on previous literature and expert opinion to select predictors, rather than selecting predictors in a purely data driven way^16^; this is especially important for datasets with limited sample size.^94^ Based on the predictors included in multiple models identified by our review, we encourage researchers to consider incorporating several candidate predictors: for diagnostic models, these include age, body temperature or fever, signs and symptoms (such as shortness of breath, headache, shiver, sore throat, and fatigue), sex, blood pressure, creatinine, basophils, neutrophils, lymphocytes, alanine transaminase, albumin, platelets, eosinophils, calcium, bilirubin, creatinine, epidemiological contact history, and potentially features derived from lung imaging. For prognostic models, these predictors include age, features derived from computed tomography scoring, lactate dehydrogenase, sex, C reactive protein, comorbidity (including hypertension, diabetes, cardiovascular disease, respiratory disease), and lymphocyte count. By pointing to the most important methodological challenges and issues in design and reporting of the currently available models, we hope to have provided a useful starting point for further studies aiming to develop new models, or to validate and update existing ones.

This living systematic review and first update has been conducted in collaboration with the Cochrane Prognosis Methods Group. We will update this review and appraisal continuously to provide up-to-date information for healthcare decision makers and professionals as more international research emerges over time.

### Conclusion

Several diagnostic and prognostic models for covid-19 are currently available and they all report good to excellent discriminative performance. However, these models are all at high risk of bias, mainly because of non-representative selection of control patients, exclusion of patients who had not experienced the event of interest by the end of the study, and model overfitting. Therefore, their performance estimates are probably optimistic and misleading. We do not recommend any of the current prediction models to be used in practice. Future studies aimed at developing and validating diagnostic or prognostic models for covid-19 should explicitly address the concerns raised. Sharing data and expertise for development, validation, and updating of covid-19 related prediction models is urgently needed.

**What is already known on this topic**

The sharp recent increase in coronavirus disease 2019 (covid-19) incidence has put a strain on healthcare systems worldwide; an urgent need exists for efficient early detection of covid-19 in the general population, for diagnosis of covid-19 in patients with suspected disease, and for prognosis of covid-19 in patients with confirmed disease
Viral nucleic acid testing and chest computed tomography imaging are standard methods for diagnosing covid-19, but are time consuming
Earlier reports suggest that elderly patients, patients with comorbidities (chronic obstructive pulmonary disease, cardiovascular disease, hypertension), and patients presenting with dyspnoea are vulnerable to more severe morbidity and mortality after infection

**What this study adds**

Three models were identified that predict hospital admission from pneumonia and other events (as proxy outcomes for covid-19 pneumonia) in the general population
Forty seven diagnostic models were identified for detecting covid-19 (34 were based on medical images); and 16 prognostic models for predicting mortality risk, progression to severe disease, or length of hospital stay
Proposed models are poorly reported and at high risk of bias, raising concern that their predictions could be unreliable when applied in daily practice

## Web extra

Extra material supplied by authors

Web appendix: Supplementary material

## Notes

Contributors: LW conceived the study. LW and MvS designed the study. LW, MvS, and BVC screened titles and abstracts for inclusion. LW, BVC, GSC, TPAD, MCH, GH, KGMM, RDR, ES, LJMS, EWS, KIES, CW, JAAD, PD, MCH, NK, AL, KL, JM, CLAN, JBR, JCS, CS, NS, MS, RS, TT, SMJvK, FSvR, LH, and MvS extracted and analysed data. MDV helped interpret the findings on deep learning studies and MMJB, LH, and MCH assisted in the interpretation from a clinical viewpoint. RS and FSvR offered technical and administrative support. LW and MvS wrote the first draft, which all authors revised for critical content. All authors approved the final manuscript. LW and MvS are the guarantors. The corresponding author attests that all listed authors meet authorship criteria and that no others meeting the criteria have been omitted.

Ethical approval: Not required.

Data sharing: The study protocol is available online at https://osf.io/ehc47/. Most included studies are publicly available. Additional data are available upon reasonable request.

The lead authors affirm that the manuscript is an honest, accurate, and transparent account of the study being reported; that no important aspects of the study have been omitted; and that any discrepancies from the study as planned have been explained.

Dissemination to participants and related patient and public communities: The study protocol is available online at https://osf.io/ehc47/.

## References

[1] Dong E Du H Gardner L An interactive web-based dashboard to track COVID-19 in real time. Lancet Infect Dis 2020:S1473-3099(20)30120-1. 10.1016/S1473-3099(20)30120-1. 32087114 PMC7159018

[2] Arabi YM Murthy S Webb S COVID-19: a novel coronavirus and a novel challenge for critical care. Intensive Care Med 2020. 10.1007/s00134-020-05955-1. 32125458 PMC7080134

[3] Grasselli G Pesenti A Cecconi M Critical care utilization for the COVID-19 outbreak in Lombardy, Italy: early experience and forecast during an emergency response. JAMA 2020. 10.1001/jama.2020.4031. 32167538

[4] Xie J Tong Z Guan X Du B Qiu H Slutsky AS Critical care crisis and some recommendations during the COVID-19 epidemic in China. Intensive Care Med 2020. 10.1007/s00134-020-05979-7. 32123994 PMC7080165

[5] Wellcome Trust. Sharing research data and findings relevant to the novel coronavirus (COVID-19) outbreak 2020. https://wellcome.ac.uk/press-release/sharing-research-data-and-findings-relevant-novel-coronavirus-covid-19-outbreak.

[6] Institute of Social and Preventive Medicine. Living evidence on COVID-19 2020. https://ispmbern.github.io/covid-19/living-review/index.html.

[7] Xie J, Hungerford D, Chen H, et al. Development and external validation of a prognostic multivariable model on admission for hospitalized patients with COVID-19. medRxiv [Preprint] 2020. 10.1101/2020.03.28.20045997

[8] DeCaprio D, Gartner J, Burgess T, et al. Building a COVID-19 vulnerability index. arXiv e-prints [Preprint] 2020. https://ui.adsabs.harvard.edu/abs/2020arXiv200307347D.

[9] Bai X, Fang C, Zhou Y, et al. Predicting COVID-19 malignant progression with AI techniques. medRxiv [Preprint] 2020. 10.1101/2020.03.20.20037325

[10] Feng C, Huang Z, Wang L, et al. A novel triage tool of artificial intelligence assisted diagnosis aid system for suspected covid-19 pneumonia in fever clinics. medRxiv [Preprint] 2020. 10.1101/2020.03.19.20039099 PMC7940949 33708828

[11] Jin C, Chen W, Cao Y, et al. Development and evaluation of an AI system for covid-19 diagnosis. medRxiv [Preprint] 2020. 10.1101/2020.03.20.20039834 PMC7547659 33037212

[12] Meng Z, Wang M, Song H, et al. Development and utilization of an intelligent application for aiding COVID-19 diagnosis. medRxiv [Preprint] 2020. 10.1101/2020.03.18.20035816

[13] Moons KG de Groot JA Bouwmeester W Critical appraisal and data extraction for systematic reviews of prediction modelling studies: the CHARMS checklist. PLoS Med 2014;11:e1001744. 10.1371/journal.pmed.1001744. 25314315 PMC4196729

[14] Moons KGM Wolff RF Riley RD PROBAST: a tool to assess risk of bias and applicability of prediction model studies: explanation and elaboration. Ann Intern Med 2019;170:W1-33. 10.7326/M18-1377. 30596876

[15] Moons KGM Altman DG Reitsma JB Transparent Reporting of a multivariable prediction model for Individual Prognosis or Diagnosis (TRIPOD): explanation and elaboration. Ann Intern Med 2015;162:W1-73. 10.7326/M14-0698. 25560730

[16] Steyerberg EW Clinical prediction models: a practical approach to development, validation, and updating. Springer US, 2019 10.1007/978-3-030-16399-0.

[17] Liberati A Altman DG Tetzlaff J The PRISMA statement for reporting systematic reviews and meta-analyses of studies that evaluate health care interventions: explanation and elaboration. PLoS Med 2009;6:e1000100. 10.1371/journal.pmed.1000100. 19621070 PMC2707010

[18] Caramelo F, Ferreira N, Oliveiros B. Estimation of risk factors for COVID-19 mortality - preliminary results. medRxiv [Preprint] 2020. 10.1101/2020.02.24.20027268

[19] Lu J, Hu S, Fan R, et al. ACP risk grade: a simple mortality index for patients with confirmed or suspected severe acute respiratory syndrome coronavirus 2 disease (COVID-19) during the early stage of outbreak in Wuhan, China. medRxiv [Preprint] 2020. 10.1101/2020.02.20.20025510

[20] Qi X, Jiang Z, YU Q, et al. Machine learning-based CT radiomics model for predicting hospital stay in patients with pneumonia associated with SARS-CoV-2 infection: a multicenter study. medRxiv [Preprint] 2020. 10.1101/2020.02.29.20029603 PMC7396749 32793703

[21] Yan L, Zhang H-T, Xiao Y, et al. Prediction of criticality in patients with severe Covid-19 infection using three clinical features: a machine learning-based prognostic model with clinical data in Wuhan. medRxiv [Preprint] 2020. 10.1101/2020.02.27.20028027

[22] Yuan M Yin W Tao Z Tan W Hu Y Association of radiologic findings with mortality of patients infected with 2019 novel coronavirus in Wuhan, China. PLoS One 2020;15:e0230548. 10.1371/journal.pone.0230548. 32191764 PMC7082074

[23] Song Y, Zheng S, Li L, et al. Deep learning enables accurate diagnosis of novel coronavirus (covid-19) with CT images. medRxiv [Preprint] 2020. 10.1101/2020.02.23.20026930 PMC8851430 33705321

[24] Yu H, Shao J, Guo Y, et al. Data-driven discovery of clinical routes for severity detection in covid-19 pediatric cases. medRxiv [Preprint] 2020. 10.1101/2020.03.09.20032219

[25] Gozes O, Frid-Adar M, Greenspan H, et al. Rapid AI development cycle for the coronavirus (covid-19) pandemic: initial results for automated detection & patient monitoring using deep learning CT image analysis. arXiv e-prints [Preprint] 2020. https://ui.adsabs.harvard.edu/abs/2020arXiv200305037G

[26] Chen J, Wu L, Zhang J, et al. Deep learning-based model for detecting 2019 novel coronavirus pneumonia on high-resolution computed tomography: a prospective study. medRxiv [Preprint] 2020. 10.1101/2020.02.25.20021568 PMC7645624 33154542

[27] Xu X, Jiang X, Ma C, et al. Deep learning system to screen coronavirus disease 2019 pneumonia. arXiv e-prints [Preprint] 2020. https://ui.adsabs.harvard.edu/abs/2020arXiv200209334X

[28] Shan F, Gao Y, Wang J, et al. Lung infection quantification of covid-19 in CT images with deep learning. arXiv e-prints 2020. https://ui.adsabs.harvard.edu/abs/2020arXiv200304655S

[29] Wang S, Kang B, Ma J, et al. A deep learning algorithm using CT images to screen for corona virus disease (covid-19). medRxiv [Preprint] 2020. 10.1101/2020.02.14.20023028 PMC7904034 33629156

[30] Song C-Y, Xu J, He J-Q, et al. COVID-19 early warning score: a multi-parameter screening tool to identify highly suspected patients. medRxiv [Preprint] 2020. 10.1101/2020.03.05.20031906

[31] Barstugan M, Ozkaya U, Ozturk S. Coronavirus (COVID-19) classification using CT images by machine learning methods. arXiv e-prints [Preprint] 2020. https://ui.adsabs.harvard.edu/abs/2020arXiv200309424B

[32] Gong J, Ou J, Qiu X, et al. A tool to early predict severe 2019-novel coronavirus pneumonia (covid-19): a multicenter study using the risk nomogram in Wuhan and Guangdong, China. medRxiv [Preprint] 2020. 10.1101/2020.03.17.20037515

[33] Jin S, Wang B, Xu H, et al. AI-assisted CT imaging analysis for COVID-19 screening: building and deploying a medical AI system in four weeks. medRxiv [Preprint] 2020. 10.1101/2020.03.19.20039354 PMC7654325 33199977

[34] Li L Qin L Xu Z Artificial intelligence distinguishes covid-19 from community acquired pneumonia on chest CT. Radiology 2020:200905. 10.1148/radiol.2020200905. 32191588 PMC7233473

[35] Lopez-Rincon A, Tonda A, Mendoza-Maldonado L, et al. Accurate identification of SARS-CoV-2 from viral genome sequences using deep learning. bioRxiv [Preprint] 2020. 10.1101/2020.03.13.990242

[36] Shi F, Xia L, Shan F, et al. Large-scale screening of covid-19 from community acquired pneumonia using infection size-aware classification. arXiv e-prints [Preprint] 2020. https://ui.adsabs.harvard.edu/abs/2020arXiv200309860S 10.1088/1361-6560/abe838 33729998

[37] Shi Y Yu X Zhao H Wang H Zhao R Sheng J Host susceptibility to severe COVID-19 and establishment of a host risk score: findings of 487 cases outside Wuhan. Crit Care 2020;24:108. 10.1186/s13054-020-2833-7. 32188484 PMC7081524

[38] Zheng C, Deng X, Fu Q, et al. Deep learning-based detection for covid-19 from chest CT using weak label. medRxiv [Preprint] 2020. 10.1101/2020.03.12.20027185

[39] Chowdhury MEH, Rahman T, Khandakar A, et al. Can AI help in screening Viral and COVID-19 pneumonia? arXiv e-prints [Preprint] 2020. https://ui.adsabs.harvard.edu/abs/2020arXiv200313145C.

[40] Sun Y Koh V Marimuthu K Epidemiological and clinical predictors of covid-19. Clin Infect Dis 2020;ciaa322. 10.1093/cid/ciaa322. 32211755 PMC7542554

[41] Martin A, Nateqi J, Gruarin S, et al. An artificial intelligence-based first-line defence against COVID-19: digitally screening citizens for risks via a chatbot. bioRxiv [Preprint] 2020. 10.1101/2020.03.25.008805 PMC7643065 33149198

[42] Wang S, Zha Y, Li W, et al. A fully automatic deep learning system for covid-19 diagnostic and prognostic analysis. medRxiv [Preprint] 2020. 10.1101/2020.03.24.20042317 PMC7243395 32444412

[43] Wang Z, Weng J, Li Z, et al. Development and validation of a diagnostic nomogram to predict covid-19 pneumonia. medRxiv [Preprint] 2020. 10.1101/2020.04.03.20052068

[44] Sarkar J, Chakrabarti P. A machine learning model reveals older age and delayed hospitalization as predictors of mortality in patients with covid-19. medRxiv [Preprint] 2020. 10.1101/2020.03.25.20043331

[45] Wu J, Zhang P, Zhang L, et al. Rapid and accurate identification of COVID-19 infection through machine learning based on clinical available blood test results. medRxiv [Preprint] 2020. 10.1101/2020.04.02.20051136

[46] Zhou Y, Yang Z, Guo Y, et al. A new predictor of disease severity in patients with covid-19 in Wuhan, China. medRxiv [Preprint] 2020. 10.1101/2020.03.24.20042119

[47] Abbas A, Abdelsamea M, Gaber M. Classification of covid-19 in chest x-ray images using DeTraC deep convolutional neural network. medRxiv [Preprint] 2020. 10.1101/2020.03.30.20047456 PMC7474514 34764548

[48] Apostolopoulos ID Mpesiana TA Covid-19: automatic detection from X-ray images utilizing transfer learning with convolutional neural networks. Physical and Engineering Sciences in Medicine, 2020, 10.1007/s13246-020-00865-4. PMC7118364 32524445

[49] Bukhari SUK, Bukhari SSK, Syed A, et al. The diagnostic evaluation of Convolutional Neural Network (CNN) for the assessment of chest X-ray of patients infected with COVID-19. medRxiv [Preprint] 2020. 10.1101/2020.03.26.20044610

[50] Chaganti S, Balachandran A, Chabin G, et al. Quantification of tomographic patterns associated with covid-19 from chest CT. arXiv e-prints [Preprint] 2020. https://ui.adsabs.harvard.edu/abs/2020arXiv200401279C. 10.1148/ryai.2020200048 PMC7392373 33928255

[51] Fu M, Yi S-L, Zeng Y, et al. Deep learning-based recognizing covid-19 and other common infectious diseases of the lung by chest CT scan images. medRxiv [Preprint] 2020. 10.1101/2020.03.28.20046045

[52] Gozes O, Frid-Adar M, Sagie N, et al. Coronavirus detection and analysis on chest CT with deep learning. arXiv e-prints [Preprint] 2020. https://ui.adsabs.harvard.edu/abs/2020arXiv200402640G.

[53] Imran A, Posokhova I, Qureshi HN, et al. AI4COVID-19: AI enabled preliminary diagnosis for covid-19 from cough samples via an app. arXiv e-prints [Preprint] 2020. https://ui.adsabs.harvard.edu/abs/2020arXiv200401275I. 10.1016/j.imu.2020.100378 PMC7318970 32839734

[54] Li K Fang Y Li W CT image visual quantitative evaluation and clinical classification of coronavirus disease (COVID-19). Eur Radiol 2020; 10.1007/s00330-020-06817-6. 32215691 PMC7095246

[55] Li X, Li C, Zhu D. COVID-MobileXpert: on-device covid-19 screening using snapshots of chest x-ray. arXiv e-prints [Preprint] 2020. https://ui.adsabs.harvard.edu/abs/2020arXiv200403042L.

[56] Hassanien AE, Mahdy LN, Ezzat KA, et al. Automatic x-ray covid-19 lung image classification system based on multi-level thresholding and support vector machine. medRxiv [Preprint] 2020. 10.1101/2020.03.30.20047787

[57] Tang Z, Zhao W, Xie X, et al. Severity assessment of coronavirus disease 2019 (covid-19) using quantitative features from chest CT images. arXiv e-prints [Preprint] 2020. https://ui.adsabs.harvard.edu/abs/2020arXiv200311988T.

[58] Zhang J, Xie Y, Li Y, et al. COVID-19 Screening on Chest X-ray Images Using Deep Learning based Anomaly Detection. arXiv e-prints 2020. https://ui.adsabs.harvard.edu/abs/2020arXiv200312338Z.

[59] Zhou M, Chen Y, Wang D, et al. Improved deep learning model for differentiating novel coronavirus pneumonia and influenza pneumonia. medRxiv [Preprint] 2020. 10.1101/2020.03.24.20043117

[60] Huang H, Cai S, Li Y, et al. Prognostic factors for COVID-19 pneumonia progression to severe symptom based on the earlier clinical features: a retrospective analysis. medRxiv [Preprint] 2020. 10.1101/2020.03.28.20045989 PMC7571455 33123541

[61] Pourhomayoun M, Shakibi M. Predicting mortality risk in patients with covid-19 using artificial intelligence to help medical decision-making. medRxiv [Preprint] 2020. 10.1101/2020.03.30.20047308 PMC7832156 33521226

[62] Zeng L, Li J, Liao M, et al. Risk assessment of progression to severe conditions for patients with COVID-19 pneumonia: a single-center retrospective study. medRxiv [Preprint] 2020. 10.1101/2020.03.25.20043166

[63] Cohen JP. Covid chestxray dataset 2020. https://github.com/ieee8023/covid-chestxray-dataset.

[64] Kaggle. COVID-19 Kaggle community contributions 2020. https://www.kaggle.com/covid-19-contributions.

[65] ClosedLoop.ai. Covid-19 vulnerability index (CV19 index) 2020. https://closedloop.ai/cv19index/.

[66] Chinese PLA General Hospital. Suspected covid-19 pneumonia diagnosis aid system 2020. https://intensivecare.shinyapps.io/COVID19/.

[67] Renmin Hospital of Wuhan University & Wuhan EndoAngel Medical Technology Co. AI diagnostic system for 2019-nCoV 2020. http://121.40.75.149/znyx-ncov/index.

[68] National Supercomputing Center of Tianjin Peunomnia CT 2020 https://ai.nscc-tj.cn/thai/deploy/public/pneumonia_ct.

[69] Sun Yat-sen University. Discriminating covid-19 pneumonia from CT images 2020. http://biomed.nscc-gz.cn/server/Ncov2019.

[70] Riley RD Ensor J Snell KIE Calculating the sample size required for developing a clinical prediction model. BMJ 2020;368:m441. 10.1136/bmj.m441. 32188600

[71] Van Calster B McLernon DJ van Smeden M Wynants L Steyerberg EW Topic Group ‘Evaluating diagnostic tests and prediction models’ of the STRATOS initiative Calibration: the Achilles heel of predictive analytics. BMC Med 2019;17:230. 10.1186/s12916-019-1466-7 31842878 PMC6912996

[72] Austin PC Lee DS Fine JP Introduction to the analysis of survival data in the presence of competing risks. Circulation 2016;133:601-9. 10.1161/CIRCULATIONAHA.115.017719. 26858290 PMC4741409

[73] Riley RD Ensor J Snell KI External validation of clinical prediction models using big datasets from e-health records or IPD meta-analysis: opportunities and challenges [correction: *BMJ* 2019;365:l4379]. BMJ 2016;353:i3140. 10.1136/bmj.i3140. 27334381 PMC4916924

[74] Debray TP Riley RD Rovers MM Reitsma JB Moons KG Cochrane IPD Meta-analysis Methods group Individual participant data (IPD) meta-analyses of diagnostic and prognostic modeling studies: guidance on their use. PLoS Med 2015;12:e1001886. 10.1371/journal.pmed.1001886. 26461078 PMC4603958

[75] Steyerberg EW Harrell FE Jr Prediction models need appropriate internal, internal-external, and external validation. J Clin Epidemiol 2016;69:245-7. 10.1016/j.jclinepi.2015.04.005. 25981519 PMC5578404

[76] Wynants L Kent DM Timmerman D Lundquist CM Van Calster B Untapped potential of multicenter studies: a review of cardiovascular risk prediction models revealed inappropriate analyses and wide variation in reporting. Diagn Progn Res 2019;3:6. 10.1186/s41512-019-0046-9. 31093576 PMC6460661

[77] Wynants L Riley RD Timmerman D Van Calster B Random-effects meta-analysis of the clinical utility of tests and prediction models. Stat Med 2018;37:2034-52. 10.1002/sim.7653. 29575170

[78] Zhou F Yu T Du R Clinical course and risk factors for mortality of adult inpatients with COVID-19 in Wuhan, China: a retrospective cohort study. Lancet 2020;395:1054-62. 10.1016/S0140-6736(20)30566-3. 32171076 PMC7270627

[79] Li K Wu J Wu F The clinical and chest CT features associated with severe and critical covid-19 pneumonia. Invest Radiol 2020. 10.1097/RLI.0000000000000672. 32118615 PMC7147273

[80] Li B Yang J Zhao F Prevalence and impact of cardiovascular metabolic diseases on COVID-19 in China. Clin Res Cardiol 2020. 10.1007/s00392-020-01626-9. 32161990 PMC7087935

[81] Jain V, Yuan J-M. Systematic review and meta-analysis of predictive symptoms and comorbidities for severe COVID-19 infection. medRxiv [Preprint] 2020. 10.1101/2020.03.15.20035360 PMC7246302 32451563

[82] Rodriguez-Morales AJ Cardona-Ospina JA Gutiérrez-Ocampo E Latin American Network of Coronavirus Disease 2019-COVID-19 Research (LANCOVID-19). Electronic address: https://www.lancovid.org Clinical, laboratory and imaging features of COVID-19: A systematic review and meta-analysis. Travel Med Infect Dis 2020:101623. 10.1016/j.tmaid.2020.101623. 32179124 PMC7102608

[83] Lippi G Plebani M Henry BM Thrombocytopenia is associated with severe coronavirus disease 2019 (COVID-19) infections: a meta-analysis. Clin Chim Acta 2020;506:145-8. 10.1016/j.cca.2020.03.022. 32178975 PMC7102663

[84] Zhao X, Zhang B, Li P, et al. Incidence, clinical characteristics and prognostic factor of patients with covid-19: a systematic review and meta-analysis. medRxiv [Preprint] 2020. 10.1101/2020.03.17.20037572

[85] Johansson MA Saderi D Open peer-review platform for COVID-19 preprints. Nature 2020;579:29. 10.1038/d41586-020-00613-4 32127711

[86] Xu B Kraemer MU Gutierrez B Open access epidemiological data from the COVID-19 outbreak. Lancet Infect Dis 2020 10.1016/s1473-3099(20)30119-5 PMC7158984 32087115

[87] Società Italiana di Radiologia Medica e Interventistica. COVID-19 database 2020. https://www.sirm.org/category/senza-categoria/covid-19/.

[88] Dutch CardioVascular Alliance. European registry of patients with covid-19 including cardiovascular risk and complications 2020. https://capacity-covid.eu/.

[89] World Health Organization. Coronavirus disease (COVID-19) technical guidance: early investigations protocols 2020. https://www.who.int/emergencies/diseases/novel-coronavirus-2019/technical-guidance/early-investigations.

[90] Infervision. Infervision launches hashtag#AI-based hashtag#Covid-19 solution in Europe 2020. https://www.linkedin.com/posts/infervision_ai-covid-medicine-activity-6650772755031613440-TqLJ.

[91] Surgisphere Corporation. COVID-19 response center 2020. https://surgisphere.com/covid-19-response-center/.

[92] Enfield K Miller R Rice T Limited utility of SOFA and APACHE II prediction models for ICU triage in pandemic Influenza. Chest 2011;140:913A 10.1378/chest.1118087.

[93] Van Calster B Vickers AJ Calibration of risk prediction models: impact on decision-analytic performance. Med Decis Making 2015;35:162-9. 10.1177/0272989X14547233. 25155798

[94] van Smeden M Moons KG de Groot JA Sample size for binary logistic prediction models: beyond events per variable criteria. Stat Methods Med Res 2019;28:2455-74. 10.1177/0962280218784726. 29966490 PMC6710621

