# Supplementary material for: Prediction models for diagnosis and prognosis of covid-19: systematic review and critical appraisal
Source: BMJ. 2020 Apr 7;369:m1328. doi: 10.1136/bmj.m1328 (PMC7222643; doi:10.1136/bmj.m1328)
Supplement: Supplementary file 1 — Web appendix: Supplementary material [file wynl056013_1.ww.pdf]

Supplementary material

Systematic review and critical appraisal of prediction models for diagnosis and prognosis of COVID-19 infection

Contents:

Search strings

Data extraction

Supplementary Table 1. Overview of the primary datasets used in included studies

Supplementary Table 2. Overview of modelling techniques for diagnosis and prognosis of COVID-19 infection

Supplementary material: search strings

Search string bioRxiv, medRxiv, arXiv: ncov or corona or Wuhan or COVID
Search string PubMed: ("Wuhan coronavirus" [Supplementary Concept] OR "COVID-19" OR "2019 ncov"[tiab] OR (("novel coronavirus"[tiab] OR "new coronavirus"[tiab]) AND (Wuhan[tiab] OR 2019[tiab])) OR 2019-nCoV[All Fields] OR (Wuhan[tiab] AND coronavirus[tiab])))))
Search string EMBASE: ncov OR (Wuhan AND corona) OR COVID

Supplementary material: data extraction

Data were extracted on the following items:

- Population (China, other).
- Intended timing of model use (screening of patients, ICU admission, etc.).
- Setting (inpatients, outpatients, suspected cases).
- Participants: study design, recruitment method, number of centres, inclusion criteria, exclusion criteria, patient age (mean and standard deviation or median and interquartile range), patient sex (n males and percentage).
- Predictors: list of candidate predictors, number of candidate predictors, number of additional degrees of freedom for candidate predictors (e.g. for categorical variables with more than two categories or for modelling continuous variables non-linearly), predictors in the final model, number of predictors in the final model, number of additional degrees of freedom in the final model.
- Outcome: definition of outcome, timing of outcome.
- Analysis:
    - Total number of participants, total number of participants with the outcome.
    - Total number of participants with any missing predictor or outcome values.
    - Method for handling missing data.
    - Method for prediction model development (logistic regression, Cox regression, neural network, tree-based, etc.).
    - Method for selection of predictors during multivariable analysis and the criteria used (e.g., p-value used for selection).
    - Handling of categorical and continuous variables.
    - Method(s) for validation (e.g. apparent, internal or external) and for optimism adjustment.
    - Performance measures (calibration, discrimination, other) resulting from validation.
    - Model presentation (coefficients and confidence intervals, final model, alternative presentation formats of the model such as a web-based tool).

- Standard signalling questions and items to assess risk of bias according to PROBAST (Moons, Wolff, et al.), on four domains:
    - participants
    - predictors
    - outcome
    - analysis

If risk of bias was high in at least one of the subdomains, overall risk of bias was judged high, as per PROBAST guidance (Moons, Wolff, et al.).

Supplementary Table 1. Overview of the primary datasets used in included studies.

	Authors	Description of dataset	Study design	Age mean ± se, median (IQR), or range	Sex (% male)
**Hospital admission in general population**					
	DeCaprio, Gartner, et al.	Medicare claims data 2015 to 2016	Administrative records	≥18	Unclear
**Diagnosis**					
	Feng, Huang, et al.	First medical centre, Chinese People's Liberation Army General Hospital, January 14 to February 9 (development) and February 10 to February 26 (validation)	Retrospective cohort	34 (IQR 29 to 42)	56%
	Lopez-Rincon, et al.	International repository of genome sequences from the China National Centre for Bioinformation (bigd.big.ac.cn/ncov), using all available samples on March 15	Unclear	Unclear	Unclear
	Meng, Wang, et al.	Various regional medical institutions in China, between December 20 to February 10	Nonnested case-control	Dev.: control: 68.5 (IQR 77 to 81), case: 46 (55 to 73); val.: control: 66 (IQR 76 to 84), case: 48 (58 to 67)	Dev.: control: 73%, case: 69%; val.: control: 69%, case: 69%
	Song, Xu, et al.	Zhijiang District of the First Affiliated Hospital of Zhejiang University School of Medicine	Unclear	38 (IQR 30 to 55)	49%
	Yu, Shao, et al.	Wuhan's Children's Hospital, February 1 to March 3	Retrospective cohort	0 to 15 (range)	61%
	Update 1:				
	Li, Fang, et al.	Hospital data from Zhuhai, China, January 18 to February 7	Unclear	45 ± 18	49%
	Martin, Nateqi, et al.	Simulated data based on published case reports	Unclear	Unclear	Unclear
	Sun, Koh, et al.	National Centre for Infectious Diseases (NCIP), Singapore, January 26 to February 16	Cross-sectional data	34 (median)	49%
	Wang, Weng, et al.	Second Affiliated Hospital and Yuying Children's Hospital of Wenzhou Medical University, Wenzhou, China, January 25 to March 3	Retrospective cohort	50 (IQR 37 to 58)	52%
	Wu, Zhang, et al.	Data from multiple sources, including Lanzhou Pulmonary Hospital, the First Hospital of Lanzhou University, Lanzhou University Second Hospital, the First People's Hospital of Lanzhou City, and Gansu Provincial Hospital	Unclear	Case: 47 (IQR 33 to 64) Control, pneumonia: 63 (IQR 47 to 75); Control, tuberculosis: 54 (IQR 33 to 68); Control, lung cancer: 61 (54 to 69)	Case: 48% Control, pneumonia: 48%; Control, tuberculosis: 60%; Control, lung cancer: 76%
	Zhou, Yang, et al.	Hospital of Wuhan, China, January 1 to February 28	Retrospective cohort	Dev.: case: 65 ± 15, control: 49 ± 16; val.: case: 66 ± 12, control 47 ± 15	Dev.: Case: 45%, Control 66%; Val.: Case: 23%, Control: 36%
	**Diagnostic imaging**				
	Barstugan, Ozkaya, et al.	Italian open data repository from the Societa Italiana di Radiologia Medica e Interventistica (sirm.org)	Unclear	Unclear	Unclear

	Chen, Wu, et al.*	Renmin Hospital of Wuhan University	Unclear	Cases: 52 (IQR 38 to 69)<br>Controls: 48 (IQR 35 to 55)	Cases: 35%<br>Controls: 56%
	Gozes, Frid-Adar, et al.	COVID cases: Wenzhou hospital (China), controls: chainz.cn registry, El Camino Hospital, California, LIDC registry (7 USA hospitals)	Nonnested case-control	Unclear	Unclear
	Jin, Chen, et al.	Wuhan Union Hospital, Western Campus of Wuhan Union Hospital, Jianghan Mobile Cabin Hospital Wuhan, January 11 to February 29 2020, LICD-IDRI registry from NCI, USA; ILD-HUG registry from University Hospitals of Geneva, Switzerland	Unclear	Unclear	Unclear
	Jin, Wang, et al.	Beijing Tsinghua Changgung Hospital, Wuhan No.7 Hospital, Zhongnan Hospital of Wuhan University, Tianyou Hospital Affiliated to Wuhan University of Science & Technology, Wuhan's Leishenshan Hospital, February 07 to February 20	Unclear	Unclear	Unclear
	Li, Qin, et al.	6 Chinese hospitals, August 16 2016 to February 17 2020 (COVID-19 cases: December 31 2019 to February 17 2020)	Unclear	49 ± 15	55%
	Shan, Gao, et al.	Shanghai Public Health Clinical Centre and other centers in Shanghai	Unclear	Unclear	High
	Shi, Xia, et al.	Tongji Hospital of Huazhong University of Science and Technology, Shanghai Public Health Clinical Centre of Fudan University, and China-Japan Union Hospital of Jilin University	Nonnested case-control	Cases: 49 ± 14 years<br>Controls: 56±14	Cases: 52%<br>Controls: 46%
	Wang, Kang, et al.	Xi'an Jiaotong University First Affiliated Hospital, Nanchang University First Hospital and Xi'an No.8 Hospital of Xi'an Medical College	Unclear	Unclear	Unclear
	Xu, Jiang, et al.	First Affiliated Hospital of Zhejiang University, No. 6 People's Hospital of Wenzhou, No. 1 People's Hospital of Wenling, January 19 to February 14	Nonnested case control	Unclear	Unclear
	Ying, Zheng, et al.	Renmin Hospital of Wuhan University, Third Affiliated Hospital , Sun Yat-Sen Memorial Hospital	Unclear	Unclear	Unclear
	Zheng, Deng, et al.	Union Hospital, Tongji Medical College, Huazhong University of Science and Technology, December 13 to January 23	Retrospective cohort	Cases:<br>51 ± 15, control: 31 ± 10	Cases: 44%, Controls: 38%
	Update 1:				
	Abbas, Abdelsamea, et al.	Images from an international Github image repository, and controls from the Japanese Society of Radiological Technology, dates unclear.	Nonnested case-control	Unclear	Unclear
	Apostolopoulos, Mpesiana.	Images from Kaggle, including data from the Radiological Society of North America (RSNA), Radiopaedia, and the Italian Society of Medical and Interventional Radiology (SIRM), dates unclear. Controls are publically available images from China and the U.S (2013-2017).	Nonnested case-control	Unclear	Unclear
	Bukhari, Bukhari et al.	University of Montreal, Canada, and U.S.A. (locations unclear), dates unclear.	Unclear	Dev.: 63 (IQR 57 to 71); Val. 61 (IQR 56 to 64)	Dev.:51%; Val.: 53%
	Chaganti, Balachandran, et al.	Data from institutions in Europe, Canada, USA, dates unspecified.	Nonnested case-control	Unclear	Unclear

	Chowdhury, Rahman et al.	Various Italian centers, locations and dates not reported.	Other	Unclear	Unclear
	Fu, Yi et al.	Wuhan Jin Yin-Tan Hospital, Zhongshan Hospital Xiamen University, the fifth Hospital of Wuhan, January 1 2015 to February 29.	Unclear	Unclear	Unclear
	Gozes, Frid-Adar et al.	Covid-19 cases: chainz.cn registry (Chinese, January to February), a second dataset from Zhejiang Province, China with both cases and controls. Lung segmentation data from El Camino Hospital, California, and University Hospitals of Geneva (unclear whether these have covid-19 cases).	Unclear	Unclear	Unclear
	Imran, Posokhova, et al.	Cough samples of unspecified origin.	Unclear	Unclear	Unclear
	Li, Zhu et al.	The international Github image repository on covid-19, combined with controls from the Radiological Society of North America Kaggle data (2018).	Nonnested case-control	Unclear	Unclear
	Hassanien, Mahdy et al.	The international Github image repository on covid-19, combined with controls from the Montgomery County X-ray Set.	Unclear	Unclear	Unclear
	Tang, Zhao et al.	Seven hospitals with different types of CT scanners, presumably Chinese.	Unclear	45 ± 17	55%
	Wang, Zha et al.	Renmin Hospital of Wuhan University, Henan Provincial People’s Hospital, the First Affiliated Hospital of Anhui Medical University, the First Hospital of China Medical University).	Retrospective cohort	Dev.: 51 ± 19<br>Val.: 49 ± 18; 58 ± 16	Dev.: 48%<br>Val.: 58%; 67%
	Zhang, Xie, et al.	The international Github image repository on covid-19, combined with controls from the publically available Chestxray8 database (2017).	Nonnested case-control	Unclear	Unclear
	Zhou, Chen et al.	8 centers in China, May 2015 to Feb 2020.	Nonnested case-control	Dev.: control: 71 (IQR 60 to 81), case: 49 (38 to 57); Val.: control: 67 (IQR 60 to 79), case: 49 (35-63)	Dev.: control: 63%, case: 57%; Val.: controll: 66%, case: 54%
**Prognosis**					
	Bai, Fang, et al.	Wuhan Pulmonary Hospital, January 3 to February 13	Retrospective cohort	53 ± 13	50%
	Caramelo, Ferreira, et al.	Simulated based on data from Wuhan, China, December 8 to February 11	Simulation	Unclear	41%
	Gong, Ou, et al:	Guangzhou Eigth People's Hospital, Zhongnan Hospital of Wuhan University, Third Affiliated Hospital of Sun Yat-sen University, January 20 to March 2	Retrospective cohort	Dev.: 49<br>Val.1: 52<br>Val.2: 42	Dev.: 47%<br>Val.1: 44%<br>Val.2: 50%
	Lu, Hu, et al.	Wuhan Hankou Hospital, January 21 to February 5	Retrospective cohort	Unclear	44%
	Qi, Jiang, et al.	5 hospitals from Ankang, Lishui, Zhenjiang, Lanzhou, Linxia between January 23 to February 8; date of last follow-up February 20	Prospective cohort	38<br>(IQR 26 to 47)	55%
	Shi, Yu, et al.	Hangzhou, Zhejiang Province, study dates unspecified, follow-up until February 17	Retrospective cohort	46 ± 19	53%
	Xie, Hungerford, et al.	Tongji Hospital and Jinyintan Hospital, admitted between January and February	Retrospective cohort	Tongji: 65 (IQR 54 to 73)<br>Jinyintan: 56 (IQR 47 to 68)	Tongji: 52%<br>Jinyintan: 35%

	Yan, Zhang, et al.	Tongji Hospital Wuhan, January 10 to February 18	Retrospective cohort	59 ± 16	59%
	Yuan, Yin, et al.	Hubei Public Health Clinical Centre; central Hospital of Wuhan, January 1 to January 25	Retrospective cohort	60 (IQR 47 to 69)	44%
	Update 1:				
	Huang, Cai et al.	Guangzhou 8th People's Hospital, January 20 to February 29	Retrospective cohort	45 ± 19	50%
	Pourhomayoun, Shakibi et al.	Publicly available data on Github from nCov-2019 Data Working Group with confirmed cases worldwide (76 countries) in a variety of settings.	Unclear	57 (mean)	Unclear
	Sakar, Chakrabarti	Kaggle data with cases from 22 countries in Asia, Australia, Europe, North America, January 13 to February 28.	Unclear	Unclear	Unclear
	Wang, Zha et al.	Renmin Hospital of Wuhan University, Henan Provincial People’s Hospital, Beijing Youan Hospital of Capital Medical University, Huangshi Central Hospital)	Retrospective cohort	Dev. 51 ± 19; Val. 50 ± 19, 48 ± 14	Dev. 48%; Val. 47%, 51%
	Zeng, Li et al.	Third People’s hospital of Shenzhen (Shenzhen, China) from January 11 to February 29	Retrospective cohort	Severe: 58.7 (11), non-severe: 46.1 (14.1)	Severe: 62% non-severe: 44%

* The study also included a prospective validation cohort of 27 consecutive patients (11 with COVID-19 pneumonia)

Supplementary Table 2. Overview of modelling techniques for diagnosis and prognosis of COVID-19 infection.

	Study	Outcome	Predictors in final model	Modelling technique
**Hospital admission in general population**				
	Decaprio, Gartner, et al.	Hospital admission for COVID-19 pneumonia (proxy events)*1	Age; sex; number of previous hospital admissions; 11 diagnostic features; interactions between age and diagnostic features	Logistic regression
	Decaprio, Gartner, et al.	Hospital admission for COVID-19 pneumonia (proxy events)*1	Age and 500+ features related to diagnosis history	Tree-based (XGBoost)
	Decaprio, Gartner, et al.	Hospital admission for COVID-19 pneumonia (proxy events)*1	500+ undisclosed features, including age, diagnostic history, social determinants of health, Charlson comorbidity index	Tree-based (XGBoost)
**Diagnosis**				
	Feng, Huang, et al.	Suspected COVID-19 pneumonia	Age, temperature, heart rate, diastolic blood pressure, systolic blood pressure, basophil count, platelet count, mean corpuscular haemoglobin content, eosinophil count, monocyte count, fever, shiver, shortness of breath, headache, fatigue, sore throat, fever classification, interleukin-6	Logistic regression (LASSO) (vs. Logistic regression (ridge), decision tree, Adaboost)
	Lopez-Rincon, et al.	COVID-19 diagnosis	Specific sequences of base pairs	Neural net (deep convolutional neural network)
	Meng, Wang, et al.	COVID-19 diagnosis	Age; activated partial thromboplastin time; red blood cell distribution width-CD; uric acid; triglyceride; serum potassium; albumin/globulin; 3-hydroxybutyrate; serum calcium	LASSO followed by logistic regression
	Song, Xu, et al.	COVID-19 diagnosis	Fever; history of close contact; signs of pneumonia on CT; neutrophil-to-lymphocyte ratio; highest body temperature; sex; (age, meaningful respiratory syndromes)	Logistic regression
	Yu, Shao, et al.	Severe disease (yes/no) defined based on clinical symptoms	Direct Bilirubin; Alanine transaminase	Tree-based (decision tree)
	Update 1:			
	Martin, Nateqi, et al.	covid-19 diagnosis	Unknown	Unclear
	Sun, Koh, et al.	covid-19 diagnosis	Age; sex; temperature; heart rate; systolic blood pressure; diastolic blood pressure; sore throat	Logistic regression
	Sun, Koh, et al.	covid-19 diagnosis	Sex; temperature; heart rate; respiration rate; diastolic blood pressure; sore throat; sputum production; shortness of breath; gastrointestinal symptoms; lymphocytes; neutrophils; eosinophils; creatinine	Logistic regression
	Sun, Koh, et al.	covid-19 diagnosis	Sex; covid-19 case contact; travel to Wuhan; travel to China; temperature; heart rate; respiration rate; diastolic blood pressure; sore throat; sputum production; gastrointestinal symptoms; CXR/CT suggestive of pneumonia; neutrophils; eosinophils; creatinine; sodium	Logistic regression
	Sun, Koh, et al.	covid-19 diagnosis	Sex; covid-19 case contact; travel to Wuhan; travel to China; temperature; heart rate; respiration rate; diastolic blood pressure; sore throat; sputum production; gastrointestinal symptoms; CXR/CT	Logistic regression

			suggestive of pneumonia; neutrophils; eosinophils; creatinine; sodium	
	Wang, Weng, et al.	covid-19 pneumonia	epidemiological history, wedge-shaped or fan-shaped lesion parallel to or near the pleura, bilateral lower lobes, ground glass opacities, crazy paving pattern, WBC count	Logistic regression (LASSO)
	Wu, Zhang, et al.	covid-19 diagnosis	Lactate dehydrogenase, calcium, creatinine, total protein, total bilirubin, basophil, platelet distribution width, kalium; magnesium; creatine kinase isoenzyme; glucose	Tree-based (random forest)
	Zhou, Yang, et al.	severe covid-19 pneumonia	Age, sex, onset-admission time, high BP, diabetes, CHD, COPD, white blood cell counts, lymphocyte, neutrophils, alanine transaminase, aspartate aminotransferase, serum albumin, serum creatinine, blood urea nitrogen, CRP	Logistic regression
**Diagnostic imaging**				
	Barstugan, Ozkaya, et al	COVID-19 diagnosis	Not applicable	Support vector machine
	Chen, Wu, et al.	COVID-19 pneumonia	Not applicable	Neural net (Unet ++)
	Gozes, Frid-Adar, et al.	COVID-19 diagnosis	Not applicable	Neural net<br>(deep convolutional neural network)
	Jin, Chen, et al.	COVID-19 diagnosis	Not applicable	Neural net<br>(various segmentation and classification models)
	Jin, Wang, et al.	COVID-19 pneumonia	Not applicable	Neural net<br>(convolutional neural network)
	Li, Qin, et al.	COVID-19 diagnosis	Not applicable	Neural net<br>(COVNet)
	Shan, Gao, et al.	Segmentation and quantification of infection regions in lung from chest CT scans.	Not applicable	Neural net (VB-Net)
	Shi, Xia, et al.	COVID-19 pneumonia	5 categories of location features from imaging: volume, number, histogram, surface, radiomics	LASSO followed by tree-based<br>(infection size-aware random forest (iSARF) method vs. logistic regression, support vector machine, Neural net.
	Wang, Kang, et al.	COVID-19 diagnosis	Not applicable	Neural net<br>(convolutional neural network)
	Xu, Jiang, et al.	COVID-19 diagnosis	Not applicable	Neural net<br>(convolutional neural network)
	Ying, Zheng, et al.	diagnosis of COVID-19 vs healthy controls	Not applicable	Neural net<br>(DRENet vs. VGG16, DenseNet, ResNet for comparison)
	Ying, Zheng, et al.	diagnosis of COVID-19 vs bacterial pneumonia	Not applicable	Neural net<br>(DRENet vs. VGG16, DenseNet, ResNet for comparison)
	Zheng, Deng, et al.	COVID19 diagnosis	Not applicable	Neural net (DeCovNet)
	Update 1:			
	Abbas, Abdelsamea, et al.	covid-19 diagnosis	Not applicable	Neural net (convolutional neural network, DeTraC)
	Apostolopoulos, Mpesiana.	covid-19 diagnosis	Not applicable	Neural net (extension of pretrained deep convolutional neural network MobileNetv2)

	Bukhari, Bukhari et al.	covid-19 diagnosis	Not applicable	Neural net (convolutional neural network ResNet-50)
	Chaganti, Balachandran, et al.	percentage lung opacity	Not applicable	Neural net (deep reinforcement learning followed by DI2N; DenseUNet)
	Chaganti, Balachandran, et al.	percentage high lung opacity	Not applicable	Neural net (deep reinforcement learning followed by DI2N; DenseUNet)
	Chaganti, Balachandran, et al.	Severity score	Not applicable	Neural net (deep reinforcement learning followed by DI2N; DenseUNet)
	Chaganti, Balachandran, et al.	Long opacity score	Not applicable	Neural net (deep reinforcement learning followed by DI2N; DenseUNet)
	Chowdhury, Rahman et al.	covid-19 vs 'normal' and viral pneumonia	Not applicable	Neural net (convolutional neural net SqueezeNet)
	Chowdhury, Rahman et al.	covid-19 vs 'normal' and viral pneumonia	Not applicable	Neural net (convolutional neural net SqueezeNet)
	Fu, Yi et al.	covid-19 diagnosis	Not applicable	Neural net (ResNet-50)
	Gozes, Frid-Adar et al.	covid-19 diagnosis	Not applicable	Neural net (U-net; ResNet-50)
	Imran, Posokhova, et al.	covid-19 diagnosis	Not applicable	Ensemble of three methods (Deep Learning-based Multi Class classifier, Classical Machine Learning-based Multi Class classifier, Deep Learning-based Binary Class classifier)
	Li, Fang, et al.	severe and critical covid-19 disease	Severity score based on CT scans	NA (external validation)
	Li, Zhu et al.	covid-19 disease	Not applicable	Neural net (convolutional neural network DenseNet-121)
	Hassanien, Mahdy et al.	covid-19 disease	Not applicable	Support vector machine
	Tang, Zhao et al.	covid-19 severe vs non-severe	Not applicable	Tree-based (Random forest)
	Wang, Zha et al.	covid-19 disease	Not applicable	Neural net
	Zhang, Xie, et al.	covid-19 disease	Not applicable	Neural net (extension of pretrained residual convolutional neural network)
	Zhou, Chen et al.	covid-19 diagnosis	Not applicable	Neural net (DenseNet121-FPN, COVID-19Net)
**Prognosis**				
	Bai, Fang, et al.	Deterioration into severe/critical disease (period unspecified)	Combination of demographics, signs and symptoms, laboratory results and features derived from CT images	Neural net (Multilayer perceptron + long short term memory vs. logistic regression, linear discriminant analysis, support vector machine, multilayer perceptron)
	Caramelo, Ferreira, et al.	Mortality (period unspecified) *2	Age; sex; presence of any comorbidity (hypertension, diabetes, cardiovascular disease, chronic respiratory disease, cancer) *3	Logistic regression

	Gong, Ou, et al.	Severe COVID-19 infection (minimum 15 day)	Age, serum LDH, CRP, variation of red blood cell distribution width, blood urea nitrogen, albumin, direct bilirubin	LASSO followed by logistic regression (vs. LASSO followed by decision tree, random forest or support vector machine)
	Lu, Hu, et al.	Mortality (12 day)	Age; C-reactive protein	Cox regression
	Qi, Jiang, et al	Hospital stay >10 days	6 features derived from CT images *3	Logistic regression
	Qi, Jiang, et al	Hospital stay >10 days	6 features derived from CT images *3	Tree-based (random forest)
	Shi, Yu, et al.	Death or severe COVID-19 (period unspecified)	Age (dichotomized); sex; hypertension	Multivariate model (not specified)
	Xie, Hungerford, et al.	Mortality (in hospital)	Age, LDH, lymphocyte count, SPO2	Logistic regression
	Yan, Zhang, et al.	Mortality (period unspecified)	Lactic dehydrogenase; lymphocyte count; high-sensitivity C-reactive protein	Tree-based (XGBoost)
	Yuan, Yin, et al.	Mortality (period unspecified)	Clinical scorings of CT images (zone, left/right, location, attenuation, distribution of affected parenchyma)	NA (external validation)
	Update 1:			
	Huang, Cai et al.	severe symptoms 3 days after admission	Underlying diseases; fast respiratory rate >24/min; elevated CRP-level (> 10mg/dL); elevated lactate dehydrogenase level (> 250U/L)	Logistic regression
	Pourhomayoun, Shakibi et al.	in-hospital mortality (period unspecified)	Unknown	Neural net (Neural Networks vs. Support Vector Machine (SVM), Random Forest, Decision Tree, Logistic Regression, and K-Nearest Neighbour (KNN))
	Sakar, Chakrabarti	death vs recovery (period unspecified)	Age, days from symptom onset to hospitalisation, from Wuhan, sex, visit to Wuhan	Tree-based (Random forest)
	Wang, Zha et al.	length of hospital stay	Age and CT features	Cox regression
	Zeng, Li et al.	severe disease progression (period unspecified)	CT features	LASSO followed by Fine and Gray
	Zeng, Li et al.	severe disease progression (period unspecified)	CT features and laboratory markers	LASSO followed by Fine and Gray

*1 Proxy events used: pneumonia (except from TB), influenza, acute bronchitis, or other specified upper respiratory infections (no COVID-19 pneumonia cases in data).
*2 Outcome and predictor data were simulated.
*3 Wavelet-HLH_gldm_SmallDependenceLowGrayLevelEmphasis, wavelet-LHH_glcm_Correlation, wavelet-LHL_glszm_GrayLevelVariance, wavelet-LLH_glszm_SizeZoneNonUniformityNormalized, wavelet-LLH_glszm_SmallAreaEmphasis, wavelet-LLH_glcm_Correlation.
