# Supplementary material for: Prediction models for diagnosis and prognosis of covid-19: systematic review and critical appraisal
Source: BMJ. 2020 Apr 7;369:m1328. doi: 10.1136/bmj.m1328 (PMC7222643; doi:10.1136/bmj.m1328)
Supplement: Supplementary file 1 [file wynl056013_4.ww.pdf]

Supplementary material

Systematic review and critical appraisal of prediction models for diagnosis and prognosis of covid-19 infection

Contents:

Search and screening

Data extraction

Supplementary Table 1. Overview of search dates, hits and inclusions

Supplementary material: search and screening

Search strings

arXiv: ncov or corona or Wuhan or COVID

Search strings for bioRxiv, medRxiv, PubMed, EMBASE are given on ispmbern.github.io/covid-19/living-review/collectingdata.html

Validation

We validated the publicly available systematic review list to examine whether it is fit for purpose by comparing it to relevant hits from bioRxiv and medRxiv when combining covid-19 search terms (covid-19, sars-cov-2, novel corona, 2019-ncov) with methodological search terms (diagnostic, prognostic, prediction model, machine learning, artificial intelligence, algorithm, score, deep learning, regression) in March 2020. All relevant hits were found on the living systematic review list. We supplemented this list with hits from PubMed by searching for "covid-19" because when we performed our initial search this term was not included in the reported living systematic review search terms for PubMed.

AI for initial screening

As the amount of covid-19 literature increased exponentially over time, a new approach was needed to keep up with the literature. The results from manual screening of title and abstracts followed by full text review for final inclusion for the records retrieved up to 7 April were used to build a classification model for covid-19 prediction model studies, using the model building function within Eppi reviewer. To this end we used the final included studies within the review as relevant studies and the rest of the covid-19 retrieved studies as irrelevant documents. Eppi reviewer uses the standard SGCClassifier in Scikit-learn on word tri-grams. As output, new documents get a percentage (from the predict_proba function) where scores close to 100 indicate a high probability of belonging to the class 'relevant document' and scores close to 0 indicate a low probability of belonging to the class 'relevant document'. The classifier was trained on the first sets of screened articles (from searches up to March 24), and tested and retrained on every following set of screened articles (search dates April 7 and May 5).

Testing on the second set (search date April 7) revealed poor positive predictive value but 100% sensitivity at a cut-off of 20%. The poor positive predictive value may be due to the broad scope of our topic (all prediction models in covid-19), poor reporting in abstracts, and

small set of included documents. The model was retrained after adding the screening data of the second set (search date April 7), which added a considerable amount of additional documents. This led to a large increase in (apparent) positive predictive value, at the cost of a lower (apparent) sensitivity, which led us to reduce the cut-off to 10%. The largest proportion of documents had a score between 0%-9%. This set did not contain any of the relevant documents. This version of the classifier with cut-off 10% was used for the initial screening of the results from the search on May 5th, and saved around 80% of the screening burden. Subsequent updating after manual screening of the results identified by the AI algorithm and testing of the classifier on the third set (search date May 5th) led to a sensitivity of 100% at cut-off 10% and reasonable positive predictive value (60%). This version of the classifier was used for the initial screening of the results from the search on June 1st 2020 and February 17th 2021 and saved around 96% of the screening burden after each of the searches.

Supplementary material: data extraction

Data were extracted on the following items:

- Population (China, other).
- Intended timing of model use (screening of patients, ICU admission, etc.).
- Setting (inpatients, outpatients, suspected cases).
- Participants: study design, recruitment method, number of centres, inclusion criteria, exclusion criteria, participant age (mean and standard deviation or median and interquartile range), participant sex (n males and percentage).
- Predictors: list of candidate predictors, number of candidate predictors, number of additional degrees of freedom for candidate predictors (e.g. for categorical variables with more than two categories or for modelling continuous variables non-linearly), predictors in the final model, number of predictors in the final model, number of additional degrees of freedom in the final model.
- Outcome: definition of outcome, timing of outcome.
- Analysis:
  - Total number of participants, total number of participants with the outcome.
  - Total number of participants with any missing predictor or outcome values.
  - Method for handling missing data.
  - Method for prediction model development (logistic regression, Cox regression, neural network, tree-based, etc.).
  - Method for selection of predictors during multivariable analysis and the criteria used (e.g., p-value used for selection).
  - Handling of categorical and continuous variables.
  - Method(s) for validation (e.g. apparent, internal or external) and for optimism adjustment.
  - Performance measures (calibration, discrimination, other) resulting from validation.
  - Model presentation (coefficients and confidence intervals, final model, alternative presentation formats of the model such as a web-based tool).

- Standard signalling questions and items to assess risk of bias according to PROBAST (Moons, Wolff, et al.), on four domains:
    - participants
    - predictors
    - outcome
    - analysis

If risk of bias was high in at least one of the subdomains, overall risk of bias was judged high, as per PROBAST guidance (Moons, Wolff, et al.).

Supplementary Table 1. Overview of Search dates, hits and inclusions.

**search date**	**publication version**	**titles retrieved**	**full text screening**	**included papers**	**included models**
13 March 2020	first pre-print (medRxiv) version	1916	/	/	/
24 March 2020	first published version (BMJ)	774	85	27	31
7 April 2020	first update (BMJ)	2213	114	24	35
5 May 2020	second update (BMJ)	9306	76	56	79
1 July 2020	third update (BMJ)	23203	169	62	87
17 February 2021	fourth update (BMJ)	89566	384	243	499
